# Gene expression profiling analysis of CRTC1-MAML2 fusion oncogene-induced transcriptional program in human mucoepidermoid carcinoma cells

**DOI:** 10.1186/s12885-015-1827-3

**Published:** 2015-10-26

**Authors:** Jie Chen, Jian-Liang Li, Zirong Chen, James D. Griffin, Lizi Wu

**Affiliations:** 1 Department of Medical Oncology, Dana-Farber Cancer Institute, Boston, MA 02115 USA; 2 Sanford Burnham Prebys Medical Discovery Institute at Lake Nona, Orlando, FL 32827 USA; 3 Deparment of Molecular Genetics and Microbiology, UF Health Cancer Center, University of Florida, Gainesville, FL 32610 USA

**Keywords:** Oncogene, CRTC1-MAML2 fusion, CREB, Gene expression profiling, Mucoepidermoid carcinoma

## Abstract

**Background:**

Mucoepidermoid carcinoma (MEC) arises from multiple organs and accounts for the most common types of salivary gland malignancies. Currently, patients with unresectable and metastatic MEC have poor long-term clinical outcomes and no targeted therapies are available. The majority of MEC tumors contain a t(11;19) chromosomal translocation that fuses two genes, *CRTC1* and *MAML2*, to generate the chimeric protein CRTC1-MAML2. CRTC1-MAML2 displays transforming activity *in vitro* and is required for human MEC cell growth and survival, partially due to its ability to constitutively activate CREB-mediated transcription. Consequently, CRTC1-MAML2 is implicated as a major etiologic molecular event and a therapeutic target for MEC. However, the molecular mechanisms underlying CRTC1-MAML2 oncogenic action in MEC have not yet been systematically analyzed. Elucidation of the CRTC1-MAML2-regulated transcriptional program and its underlying mechanisms will provide important insights into MEC pathogenesis that are essential for the development of targeted therapeutics.

**Methods:**

Transcriptional profiling was performed on human MEC cells with the depletion of endogenous CRTC1-MAML2 fusion or its interacting partner CREB via shRNA-mediated gene knockdown. A subset of target genes was validated via real-time RT-PCR assays. CRTC1-MAML2-perturbed molecular pathways in MEC were identified through pathway analyses. Finally, comparative analysis of CRTC1-MAML2-regulated and CREB-regulated transcriptional profiles was carried out to assess the contribution of CREB in mediating CRTC1-MAML2-induced transcription.

**Results:**

A total of 808 differentially expressed genes were identified in human MEC cells after CRTC1-MAML2 knockdown and a subset of known and novel fusion target genes was confirmed by real-time RT-PCR. Pathway Analysis revealed that CRTC1-MAML2-regulated genes were associated with network functions that are important for cell growth, proliferation, survival, migration, and metabolism. Comparison of CRTC1-MAML2-regulated and CREB-regulated transcriptional profiles revealed common and distinct genes regulated by CRTC1-MAML2 and CREB, respectively.

**Conclusion:**

This study identified a specific CRTC1-MAML2-induced transcriptional program in human MEC cells and demonstrated that CRTC1-MAML2 regulates gene expression in CREB-dependent and independent manners. Our data provide the molecular basis underlying CRTC1-MAML2 oncogenic functions and lay a foundation for further functional investigation of CRTC1-MAML2-induced signaling in MEC initiation and maintenance.

**Electronic supplementary material:**

The online version of this article (doi:10.1186/s12885-015-1827-3) contains supplementary material, which is available to authorized users.

## Background

Mucoepidermoid carcinoma (MEC) is the most common malignant form of salivary gland tumors. MEC also develops in various sites such as lung, thyroid, breast, skin, pancreas, esophagus, and cervix [1–3]. Patients with unresectable and metastatic MEC have poor long-term clinical outcomes, and no targeted therapy is currently available. A majority of MEC cases are associated with a specific chromosomal t(11;19)(q14-21;p12-13) translocation that joins exon 1 of the *CRTC1* gene to exons 2–5 of the *MAML2* gene, resulting in the expression of a new *CRTC1-MAML2* fusion gene [4–7]. CRTC1 belongs to the three-member CRTC (CREB-regulated transcription co-activator) family that co-activates CREB-mediated transcription [8, 9]. CRTC co-activators have critical roles in regulating metabolism, aging, memory, and cancer [10–12]. MAML2 belongs to the three-member MAML (mastermind-like) family that co-activates Notch receptor-induced transcription. MAML co-activators are critical in development and diseases including cancer [13, 14]. In human MEC, the CRTC1-MAML2 fusion protein consists of the 42-aa amino terminal CREB binding domain (CBD) of CRTC1 and the 981-aa carboxyl terminal transcriptional activation domain (TAD) of MAML2 [15]. Current evidence implicates CRTC1-MAML2 fusion as a major etiologic molecular event and a therapeutic target in human MEC. First, the CRTC1-MAML2 fusion induced colony formation of cultured epithelial RK3E cells and the resulting fusion-transformed RK3E cells were capable of forming subcutaneous tumors in immune-compromised mice [15–17], indicating a role of the CRTC1-MAML2 fusion in epithelial cell transformation. Second, depletion of endogenous CRTC1-MAML2 fusion significantly reduced MEC cell growth and survival *in vitro* and the growth of human MEC xenografts *in vivo* [18], demonstrating a critical role of the CRTC1-MAML2 fusion oncogene in the maintenance of MEC cancerous phenotypes. Therefore, these studies strongly suggest that CRTC1-MAML2 has an essential role in MEC initiation and maintenance.

The CRTC1-MAML2 fusion oncoprotein is a nuclear protein and functions as a transcriptional co-activator [15]. CRTC1-MAML2 fusion interacts with the transcription factor CREB through the CRTC1 CBD domain and activates CREB-mediated transcription through the MAML2 TAD domain [16, 19], thereby constitutively activating CREB-mediated transcription. Aberrant CREB activity contributes at least partially to CRTC1-MAML2’s transforming activity [16]. More recent studies showed that CRTC1-MAML2 had CREB independent activities through the interaction of other nuclear factors such as AP-1 [20] and MYC [21]. These data support that CRTC1-MAML2 drives oncogenic transformation by impinging on multiple gene regulatory pathways.

However, the molecular mechanisms that account for the CRTC1-MAML2 fusion oncogene in tumorigenesis have not been characterized systematically. The CRTC1-MAML2 fusion has transcriptional co-activation activity and its functions are mediated in large part by changes in gene expression. Therefore, in this study we performed global gene expression profiling and examined the transcriptional program induced by the CRTC1-MAML2 fusion oncoprotein that contributes to MEC development and maintenance. Specifically, we interrogated changes in gene expression patterns in MEC cells caused by the knockdown of CRTC1-MAML2 fusion expression. We also determined the extent of CRTC1-MAML2/CREB interaction in target gene regulation through comparative analysis of transcriptional profiles of MEC cells with CREB knockdown or CRTC1-MAML2 knockdown. Our study revealed target genes and mechanisms of CRTC1-MAML2 that potentially contribute to MEC pathogenesis.

## Methods

### Plasmids

The pSuperRetro-GFP/Neo vector-based shRNAs targeting the MAML2 TAD domain of CRTC1-MAML2 (shMAML2) or control shRNA targeting luciferase gene (shLuc) were previously described [18]. The pLKO.1-based lentiviral constructs targeting the MAML2 portion of CRTC1-MAML2 (RHS4533-NM_032427) were purchased from Open Biosystems. Two good shMAML2 were identified: shM2-1 (TRCN0000118837) targeting the 3’ UTR with target sequence 5’-CCCTGTCTAAACTCCAGGATA-3’; and shM2-3 (TRCN0000118839) targeting the exon 5 of MAML2 5’-CCCAAAGCAATTGTTAGCAAA-3’. Two pLKO.1-based shRNAs targeting the exon 1 of MAML2 were generated with the following shRNA targeting sequences: 5’-GGACGATATGAACGAGGTA-3’ (shM2-B1) and 5’-TCGTTCATATCGTCCTTCA-3’ (shM2-C1). The pLKO.1-based lentiviral shRNA constructs targeting CREB (RHS4533-NM_004379) were obtained from Open Biosystems and two good shCREB includes shCREB-B9 (TRCN0000011085) with a target sequence 5’-AATCAGTTACACTATCCACTG-3’ and shCREB-G9 (TRCN0000007308) with a target sequence 5’-TAACTGTTAGATTTATCGAGC-3’. The pKLO.1-scramble shRNA control vector (shCtl) was obtained from Addgene.

### Cell culture

HSY (fusion-negative cell), H3118 (fusion-positive MEC of the parotid gland), H292 (fusion-positive MEC of the lung), 293 T (human 293 cells expressing SV40 large T antigen), and 293FT (derived from human 293 T) were cultured in Dulbecco's modified Eagle's medium (DMEM; Sigma) supplemented with 10 % inactivated fetal bovine serum (Atlanta Biologicals) and 1 % penicillin/streptomycin (Mediatech). Cells were grown at 37 °C under 5 % CO_2_.

### Retroviral and lentiviral transduction

For retroviral production, 293 T cells were first plated at 3 × 10^6^ cells in 10-cm culture dishes and transfected next day with 8 μg of retroviral constructs and 2 μg of each packaging plasmids pMD.MLV and pMD2-VSV-G. For lentiviral production, 293FT cells were transfected with lentiviral vectors with pSPAX2 and pMD2.G packaging plasmids. Superfect transfection reagents (QIAGEN) were used. Viruses were collected at 48 and 72 h post-transfection. Target cells were subsequently infected with the viruses in fresh complete medium containing 8 μg/ml polybrene (Sigma) for 6 to 8 h twice on two consecutive dates. For cells infected with pSuperRetro-GFP/Neo plasmids, GFP-positive cells were FACS-sorted at 72 h after viral infection. For cells infected with pLKO.1 shRNA viruses, cells were harvested at 72 h after viral infection for analysis.

### Microarray experiments

Total RNA was extracted using TRIzol reagent (Invitrogen) and purified by RNeasy Mini kit (QIAGEN). The yield and quality of RNA were assessed using spectrophotometry and the Agilent 2100 Bioanalyzer (Agilent Technologies). Microarray experiments including cDNA preparation, hybridization, scanning, and image analysis of Affymetrix GeneChip HG-U133 plus 2.0 microarrays were performed in the Microarray Core facility at Dana-Farber Cancer institute according to the manufacturer’s protocol (Affymetrix). Experiments were performed either in duplicate or triplicate.

### Microarray analysis

Statistical tests were carried out using R/BioConductor software [22]. Data pre-processing and normalization were performed using the affy package [23]. Raw data were normalized using the Robust Multichip Analysis (RMA) approach. The detection of a present or absent call for a gene in a sample was determined using the Affymetrix GCOS software. Probe-sets defined as “absent” calls across all the samples were removed from data analysis to reduce the false positives. To identify differentially expressed genes, the linear modeling approach and empirical Bayes statistics as implemented in the limma package [24] were employed. The p-values were adjusted using the Benjamini and Hochberg method [25]. Genes with an absolute fold change of > =2 and a p-value < 0.05 were considered as significantly differentially expressed. Hierarchical clustering of the differentially expressed gene list was computed on log-transformed normalized data. The microarray data were deposited in NCBI Gene Expression Omnibus (GEO Series GSE59795).

### Functional enrichment analysis

Differentially expressed genes were analyzed in the context of biological functions, pathways, and diseases using the Ingenuity Pathway Analysis software (IPA; Ingenuity Systems Inc) [26]. The p-value was calculated using Fisher’s exact test to determine a potential significant association between differentially expressed genes and specific functional categories. A p-value < 0.05 was considered to be statistically significant. For upstream regulator analysis, z-scores were calculated to predict upstream regulators such as transcription factors, kinases, compounds or drugs. The z-score is dependent on gene expression in the input dataset and the knowledge of expected effects between regulators and their known target genes in the Ingenuity Knowledge Base. The statistically significant overlap between the dataset genes and the known target genes was also calculated by Fisher’s Exact test. An upstream regulator with a z-score of >2 (or < −2) and *p* <0.01 was considered as significantly “activated” or “inhibited”.

The fusion-regulated and CREB-regulated genes generated from our microarray data were also subjected for Gene Set Enrichment Analysis (GSEA) analysis to identify potential functional enrichment. GSEA consists of three major steps: calculation of an enrichment score (ES), estimation of the significance level of the ES, and adjustment for multiple hypothesis testing [27]. For this study, the curated motif gene sets (C3:TFT, version 3.1) in Broad Molecular Signature Database (MSigDB) [28] were used to determine enriched transcription factors in the fusion or CREB knockdown datasets. This motif gene set included genes annotated as transcription factor (TF) targets from TRANSFAC database. Genes were ranked according to the correlation of gene expression with fusion or CREB knockdown using the signal-to-noise ratio, and gene set permutations were used for assessments of significance. Gene sets with FDR p-value <0.25 were considered as significantly enriched.

### Real-time RT-PCR

Real-time RT-PCR was performed as previously described [18]. RNA was reverse transcribed into cDNA using a GeneAmp RNA PCR kit (Applied Biosystems). Real-time PCR was performed with the StepOne Real-Time PCR System (Applied Biosystems) using the SYBR Green PCR Core Reagents Kit (Applied Biosystems). GAPDH was used as an internal control for the normalization of gene expression. The primers used are as follows: CRTC1-MAML2 primers (Forward, 5’-TTCGAGGAGGTCATGAAGGA-3’; Reverse, 5’-TTGCTGTTGGCAGGAGATAG-3’); MAML2 exon 1 primers (Forward, 5’-CTCCCCCACAACTTCTCCAC-3’; Reverse, 5’-CTCAGTGTTCAGGGCCACAT-3’); AREG primers (Forward, 5'-GCCGCTGCGAAGGACCAATG-3'; Reverse, 5’-CCAGCAGCATAATGGCCTGAGCC-3’); LINC00473 primers (Forward, 5'-AAACGCGAACGTGAGCCCCG-3'; Reverse, 5’-CGCCATGCTCTGGCGCAGTT-3’); DMBT1 primers (Forward, 5'- TGTCCTGGATGACGTGCGCTG-3'’; reverse, 5'-GGTCGGCAACGTGTCTGAGCA-3'); STC1 primers (Forward, 5'-TGATCAGTGCTTCTGCAACC-3'; Reverse, 5’-GTTGAGGCAACGAACCACTT-3’); PDE4B primers (Forward, 5'-CCGATCGCATTCAGGTCCTTCGC-3'; Reverse, 5’- TGCGGTCTGTCCATTGCCGA-3’); RUNX3 primers (Forward, 5'- CAGAAGCTGGAGGACCAGAC-3'; Reverse, 5’-TCGGAGAATGGGTTCAGTTC-3’); PTGS1 primers (Forward, 5’-GCTCTGGTTCTTGCTGTTCC-3’; Reverse, 5’-TGGTGCTGGCATGGATAGTA-3’); TGFB2 primers (Forward, 5'-CCGCCCTTCTTCCCCTCCGAA-3'; Reverse, 5’- CGGGATGGCATCAAGGTACCCAC-3’); ODC1 primers (Forward, 5'-TGTTGAGCGCTGTGACCTGCC-3'; Reverse, 5’-ATGAGTTGCCACGCAGGCCC-3’); CDK6 primers (Forward, 5'-CCTGCAGGGAAAGAAAAGTGCAATG-3'; Reverse, 5’- AGCGAGCCGATCCCTCCTCT-3’); and GAPDH primers (Forward, 5’-CAATGACCCCTTCATTGACC-3’; Reverse, 5’-GACAAGCTTCCCGTTCTCAG-3’). Significant differences between two groups were analyzed using Student’s *t*-test. A p-value < 0.05 was considered statistically significant.

### Western blotting

Cellular protein extracts were prepared as previously described [18]. For immunoblot analysis, protein extracts were fractionated in SDS-polyacrylamide gels and then electro-transferred to nitrocellulose membranes. The membranes were blocked for 1 h in a buffer containing 10 mM Tris, pH 8.0, 150 mM NaCl, 0.05 % Tween 20, and 5 % nonfat dry milk. The membranes were then incubated with the antibodies (anti-MAML2 TAD, Cell signaling CST-4618; or anti-β-actin, Santa Cruz sc-47778) overnight at 4 °C, washed, and then incubated with a horseradish peroxidase-conjugated secondary antibody for 1 h at RT. The protein bands were detected using enhanced chemiluminescence (Pierce).

## Results

### Microarray analysis identified downstream target genes specifically regulated by the CRTC1-MAML2 fusion oncoprotein

The *CRTC1-MAML2* fusion oncogene was implicated in tumor initiation and maintenance of human MEC [16–18]. Previously, a number of target genes were found differentially expressed in cervical cancer Hela cells after CRTC1-MAML2 was over-expressed [16, 19], which uncovered an important activity of CRTC1-MAML2 in constitutive activation of CREB-mediated gene expression. However, the relevant physiological targets of the CRTC1-MAML2 fusion oncoprotein in human MEC cells remained to be systematically identified. The identification of the fusion target genes and pathways will be important to explain specific and frequent association of CRTC1-MAML2 and MEC. We hypothesized that CRTC1-MAML2 fusion oncogene induced a specific transcriptional program that contributes to MEC initiation and maintenance. To identify the CRTC1-MAML2-induced transcriptional program, we examined the effect of CRTC1-MAML2 depletion on gene expression profile changes in human fusion-positive MEC cells by microarray analysis.

**Fig. 1:**
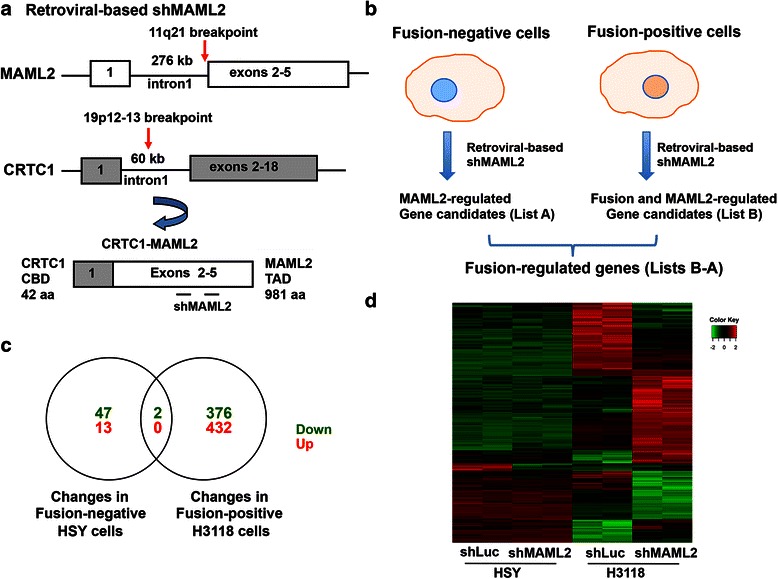
Transcriptional profiling analysis revealed target gene candidates regulated by the CRTC1-MAML2 fusion oncogene in human MEC cells. **a** The t(11;19) translocation fuses exon 1 of the *CRTC1* gene to exons 2–5 of the *MAML2* gene and generates the CRTC1-MAML2 fusion consisting of CRTC1 CREB binding domain (CBD) and MAML2 transcriptional activation domain (TAD). **b** The strategy in identifying CRTC1-MAML2-regulated genes by microarray analysis was shown. Fusion-negative cells (HSY) and fusion-positive cells (H3118) were infected with retroviral-mediated MAML2 shRNA-IRES-GFP (co-expressing MAML2 shRNA and GFP) or luc shRNA-IRES-GFP (co-expressing luciferase shRNA and GFP that serves as a control) for 72 h. GFP-positive cells were then FACS-sorted, and RNA samples were collected for microarray analysis using HG-U133 Plus 2.0 arrays. Comparison of MAML2-knockdown HSY cells and the control cells resulted in MAML2-regulated gene candidates (list A), while comparison of fusion/MAML2-knockdown H3118 cells and the controls led to fusion/MAML2-regulated genes (list B). Based on these two lists of target genes, fusion-regulated genes were identified by filtering out the list A from the list B (List: B-A). **c**, **d** Venn diagram (**c**) and heatmap (**d**) show differentially expressed genes in both fusion-negative HSY and fusion-positive cells after shMAML2 transduction. Two biological replicates were analyzed. This analysis led to the identification of a total of 808 fusion-regulated candidate genes, with 376 down-regulated and 432 up-regulated genes

The *CRTC1-MAML2* fusion gene consists of exon 1 of the *CRTC1* gene fused to exons 2–5 of the *MAML2* gene (Fig. 1a). Ideally, shRNA that specifically targets the fusion junction should be used to investigate the specific effect of loss-of-function of CRTC1-MAML2. However, we failed to obtain any shRNA that could cause specific, effective knockdown of CRTC1-MAML2 expression. Therefore, we took an approach that allowed us to investigate CRTC1-MAML2-regulated genes in human MEC cells (Fig. 1b). We utilized pSuperRetro-based retroviruses co-expressing shRNA targeting MAML2-TAD region (shMAML2) and GFP from a bicistronic transcript. shMAML2 led to MAML2 knockdown in fusion-negative HSY cells and knockdown of both MAML2 and CRTC1-MAML2 in fusion-positive H3118 cells [18]. The pSuperRetro-based shRNA retroviruses co-expressing luciferase shRNA (shLuc) and GFP were used as controls. Here, we first performed retroviral infection of fusion-negative HSY cells and fusion-positive H3118 cells using control shLuc or shMAML2 retroviruses in duplicate. At 72 h after infection, transduced cells were FACS-sorted for GFP expression to enrich cell populations containing shRNAs, as shRNAs were co-expressed with GFP in the transduced cells. The sorted GFP-expressing cells were processed for the isolation of RNA. Subsequently, gene expression profiling was performed using Affymetrix human genome U133 plus 2.0 arrays that covered approximately 38,500 well-characterized human genes.

We adopted a criteria with an absolute fold-change > = 2.0 and a p-value < 0.05 to define the differentially regulated genes. The number of differentially expressed genes and overall gene expression patterns were shown in Venn diagram, heatmap (Fig. 1c-d) and volcano plots (Additional file 1: Figure S1). Differentially expressed genes in fusion-negative cells after the transduction of shMAML2 viruses in comparison with shLuc control represented MAML2-regulated gene candidates (Fig. 1b), which contained 13 up-regulated and 49 down-regulated genes (Fig. 1c). On the other hand, differentially expressed genes between shMAML2-expressing and shLuc-expressing fusion-positive cells represented fusion/MAML2-regulated genes (Fig. 1b), which consisted of a total of 432 up-regulated and 378 down-regulated genes (Fig. 1c). To determine genes that were specifically regulated by CRTC1-MAML2 fusion, we filtered out 2 genes from fusion/MAML2-regulated gene list that showed the same regulated direction as in MAML2-regulated genes (Fig. 1b-c, Additional file 2: Table S1). Finally, our analysis identified a total of 808 differentially expressed genes after CRTC1-MAML2 knockdown in human fusion-positive MEC H3118 cells, including 376 down-regulated genes and 432 up-regulated genes (Additional file 2: Table S1).

### Validation of the CRTC1-MAML2 target genes from microarray analysis

**Fig. 2:**
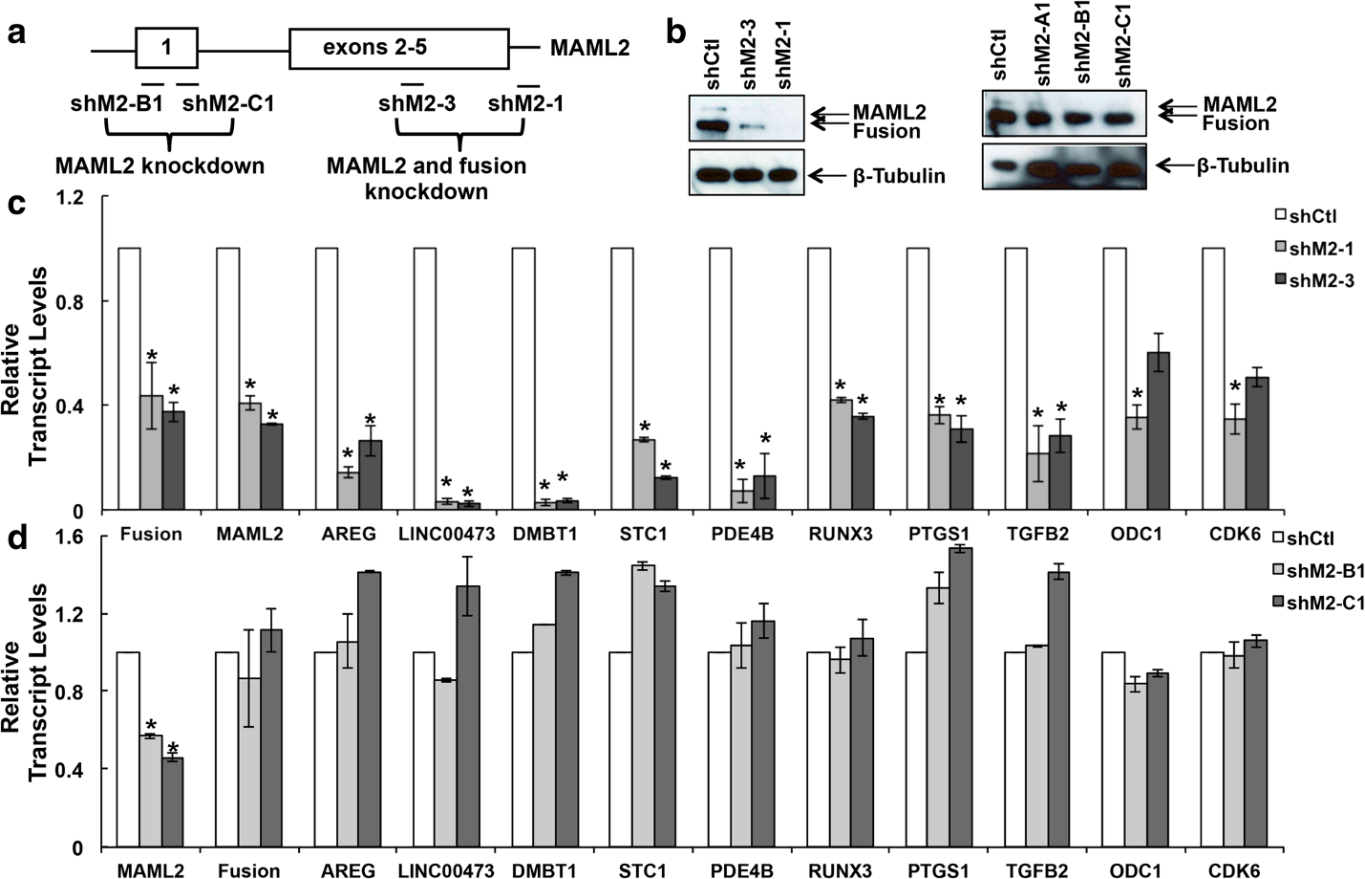
Real-time RT-PCR assays validated a subset of CRTC1-MAML2 fusion-regulated genes identified from microarray analysis. **a** Lentiviral pLKO.1-based shRNAs targeting various regions of the *MAML2* gene were indicated. These shRNAs and scramble control shRNA (shCtl) lentiviruses were used to infect the CRTC1-MAML2 fusion-expressing H3118 MEC cells and the infected cells were processed to isolate protein lysates for Western blotting analysis and RNA for real-time RT-PCR assays. **b** Western blot analysis showed shM2-1 or shM2-3 led to the knockdown of MAML2 and fusion, whereas that shM2-B1 or shM2-C1 caused MAML2 knockdown only. It is noted that another shRNA, shM2-A1 targeting the exon 1 of MAML2 did not cause MAML2 knockdown. **c**, **d** Real-time RT-PCR analyses showed that knockdown of both CRTC1-MAML2 fusion and MAML2 in H3118 MEC cells led to reduced transcripts levels of a known target AREG and a subset of novel fusion target genes, including LINC00473, DMBT1, STC1, PDE4B, RUNX1, PTGS1, TGFB2, ODC1, and CDK6 (**c**), whereas MAML2 knockdown in H3118 MEC cells did not significantly affect their expression (**d**). The level of CRTC1-MAML2 fusion transcript was determined using a primer set that spans the chromosomal translocation breakpoint. The level of MAML2 knockdown was determined using the primers that amplify the exon 1 of MAML2. Data are presented as mean ± S.E. (*n* = 3, *p < 0.05)

The CRTC1-MAML2 fusion oncoprotein is a transcriptional co-activator [15, 16], so we next focused on genes whose transcription was down regulated in response to CRTC1-MAML2 knockdown in human MEC cells for the validation of the microarray results. From the CRTC1-MAML2 regulated gene list (Additional file 2: Table S1), we have selected several genes that were known to be critical players in tumor development and progression, or therapeutic targets with clinical or preclinical inhibitors available, or potential diagnostic or prognosis markers (Table 1). A total of 10 CRTC1-MAML2-regulated target gene candidates were tested in human MEC by RT-qPCR, including a recently identified target AREG [16] and 9 novel targets including LINC00473, DMBT1, STC1, PDE4B, RUNX3, PTGS1, TGFB2, ODC1, and CDK6. Specifically, we used an independent set of lentiviral-based shRNAs, including 2 shRNAs targeting respective sequences in the exon 5 and 3’ UTR of the *MAML2* gene (shM2-3, shM2-1) and 2 shRNAs targeting the exon 1 of *MAML2* (shM2-B1, shM2-C1) (Fig. 2a). Scramble shRNA was used as a control (shCtl). Fusion-positive H3118 MEC cells were infected twice with these lentiviruses on two consecutive days. RNA and protein samples were then isolated at 72 h after the first infection. As expected, both shM2-3 and shM2-1 lentiviruses caused knockdown of MAML2 and CRTC1-MAML2 fusion in fusion-positive MEC cells, while shM2-B1 and shM2-C1 led to only MAML2 knockdown at both the transcript and protein levels (Fig. 2c-d). We found that knockdown of both CRTC1-MAML2 and MAML2 via shM2-1 or shM2-3 caused down-regulation of the known AREG gene as well as 9 novel targets including LINC00473, DMBT1, STC1, PDE4B, RUNX3, PTGS1, TGFB2, ODC1, and CDK6 in fusion-positive MEC H3118 cells (Fig. 2c). However, expression levels of these genes were not significantly affected when only MAML2 was knocked down using shRNAs that target the exon 1 of the MAML2 gene (shM2-B1 and shM2-C1) (Fig. 2d). Therefore, our data indicate that expression of these 10 target genes was regulated by the CRTC1-MAML2 fusion in human MEC H3118 cells, which was consistent with the gene expression changes shown by our microarray data. Moreover, using similar strategies, we subsequently performed gene validation in a second MEC cell line, a human lung MEC H292 cell line. We found that 6 out of these 10 genes were regulated by CRTC1-MAML2, while 4 of them including RUNX3, PTGS1, TGFB2 and CDK6 were not (Additional file 1: Figure S2). These data suggest that CRTC1-MAML2 regulates common target genes as well as specific cell context dependent targets in different MEC cells.

**Table 1:** A subset of CRTC1-MAML2 target gene candidates is shown

Gene symbol	Gene title	H3118 Fusion & MAML2 KD/Control	HSY MAML2 KD/Control	Subcelluar localization	Type(s)	Drug(s)	Biomarker application(s)
LINC00473^a^	long Intergenlc non-protein coding RNA 473	−37.12	−1.03	Unknown	other		
				Plasma	transmembrane		
DMBT1^a^	deleted in malignant brain tumors 1	−35.73	1.16	Membrane	receptor		
					secreted		
STC1^a^	stanniocalcin 1	−21.74	1.03	Extracellular	glycoprotein		Diagnosis
PDE4B	phosphodiesterase 4B, cAMP-specific	−8.41	1.09	Cytoplasm	enzyme	dyphylline, nitroglycerin	
					transcription		
RUNX3	runt-related transcription factor 3	−4.88	1.03	Nucleus	regulator		Diagnosis
							Prognosis
PTGS1^a^	prostaglandin-endoperoxide synthase 1	−3.89	1.31	Cytoplasm	enzyme	acetaminophen, aspirin	Progression
							Prognosis
PDE4D	phosphodiesterase 4D, cAMP-specific	−2.93	1.05	Cytoplasm	enzyme	dyphylline, nitroglycerin	Prognosis
							Diagnosis
							Efficacy
CA9^a^	carbonic anhydrase IX	−2.77	1.2	Nucleus	enzyme	CG250, Methazolamide Tazarotene	Prognosis
							Diagnosis
ODC1	ornithine decarboxylase 1	−2.54	−1.06	Cytoplasm	enzyme	Eflornithine	Efficacy
						AP-12009	
TGFB2^a^	transforming growth factor, beta 2	−2.48	1.21	Extracellular	growth factor	Lerdelimumab	Efficacy
							Diagnosis
AREG^a^	Amphiregulin	−2.23	1.11	Extracellular	growth factor	Cetuximab, gefitinib	Efficacy
							Prognosis
TYMS^a^	thymidylate synthetase	−2.1	−1.03	Nucleus	enzyme	Flucytosine, 5-fluorouracil	Diagnosis
							Efficacy
							Prognosis
CDK6	cvclin-deDendent kinase 6	−2.07	1.02	Nucleus	kinase	PD-0332991	Proanosis
						flavopiridol	

The fold changes of gene expression were shown for fusion-positive MEC H3118 cells with fusion/MAML2 knockdown (KD), and fusion-negative HSY cells with MAML2 KD

^a^ Genes down-regulated in H3118 cells with CREB KD

### Pathway analysis of CRTC1-MAML2-regulated genes revealed a role of CRTC1-MAML2 in regulating multiple signaling pathways that are important in tumorigenesis

**Fig. 3:**
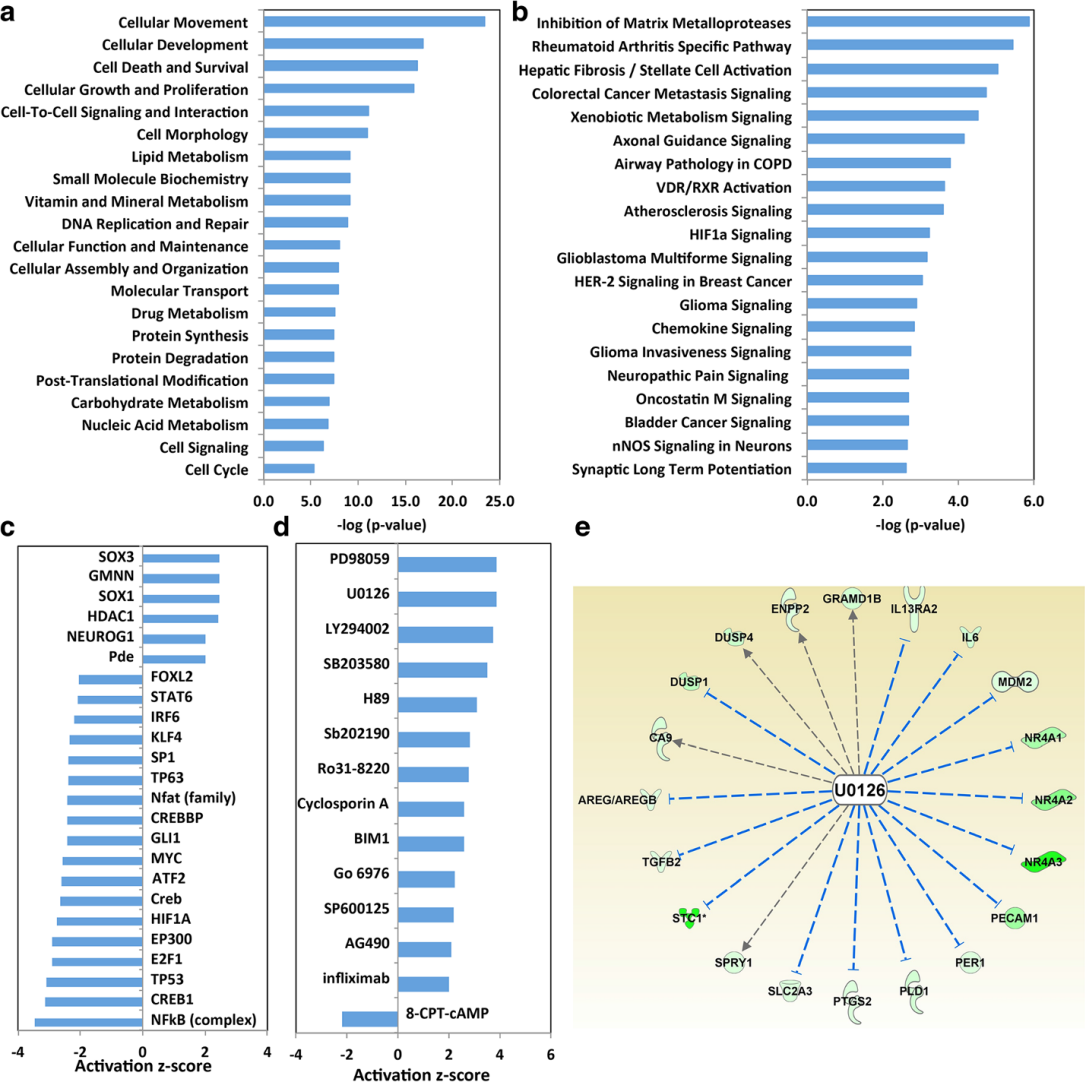
Pathway analysis revealed that CRTC1-MAML2 fusion induces critical cancer cell signaling. **a** Functional classification of fusion-regulated genes (Additional file 2: Table S1) was performed using Ingenuity Pathway Analysis (IPA). The top 20 Molecular and Cellular Functions were ranked based on p-value, and the bars represent inverse log of the p-value (x-axis). **b** Top 20 Canonical Signaling Pathways that were enriched in fusion-regulated genes were shown. These pathways were ranked based on p-value, and the bars represent inverse log of the p-value (x-axis). **c** Upstream regulators analysis identified several transcription regulators regulated CRTC1-MAML2 fusion targeted genes. Changes in the activation status of transcription regulators were plotted based on their activation z-score from IPA. Positive z-score indicates activation and negative z-score indicates inhibition. **d** Upstream regulators analysis identified several drugs or kinase inhibitors regulated CRTC1-MAML2 fusion targeted gene expression. **e** MAPK inhibitor (U0126) regulated several CRTC1-MAML2 fusion down-regulated genes (Activation z-score: 3.89; p-value of overlap: 1.65E-5), suggesting that fusion function can be blocked by the inhibition of the MAPK pathway

To examine CRTC1-MAML2-regulated genes from the microarray data in a biologically relevant manner, we next performed functional enrichment analysis. Here, the CRTC1-MAML2 fusion-regulated gene set (Additional file 2: Table S1) was subjected to Ingenuity Pathway Analysis (IPA) in identifying over-represented biological functions and pathways. The top 20 enriched molecular and cellular functions in the CRTC1-MAML-regulated genes were shown in Fig. 3a, revealing that CRTC1-MAML2 fusion had associated functions in cellular processes such as cellular movement, development, death and survival, growth and proliferation, cell-to-cell signaling and interaction, and metabolism. The top over-represented canonical signaling pathways included modulation in commonly observed “Cancer” signatures, such as the regulation of matrix metalloproteases, cancer metastasis signaling, HIF1α signaling, and HER-2 signaling (Fig. 3b).

IPA upstream regulator analysis allows the identification of the “activation or inhibition state” of specific upstream regulators in a gene set. CRTC1-MAML2 fusion is a transcriptional co-activator, so we focused on those genes that were activated by CRTC1-MAML2. Therefore, we used the fusion depletion-induced down-regulated gene set in Additional file 2: Table S1, and predicted the altered states of transcription regulator families. Using a criteria with a z-score of > 2.0 or < −2.0 and a p-value < 0.01, our analysis predicted the inhibition of several transcription regulators in fusion-knockdown gene set including known CRTC1-MAML2 interactors such as CREB, EP300, CREBBP, and MYC [16, 21], and transcription regulators that were not previously linked with CRTC1-MAML2 such as NF-κB complex, TP53, E2F1, HIF1A, ATF2, GLI1, NF-AT, TP63, KLF4, IRF6, STAT6, and FOXL2 (Fig. 3c). These data strongly suggest that CRTC1-MAML2 interacts functionally with multiple signaling pathways associated with these regulators, which could contribute to CRTC1-MAML2 fusion oncogenic functions. It should be noted that JUN was also identified outside a cutoff score but with a z-score of −1.7, supporting a reported interaction of CRTC1-MAML1 and AP-1 (FOS/JUN) [20].

Moreover, this analysis predicted that biologic drugs such as cyclosporine A (an immunosuppressant) and infliximab (a monoclonal antibody against TNF-α), and small-molecule kinase inhibitors such as MAPK inhibitors (PD98059 and U0216), PI-3 K inhibitor (LY294002), p38 MAPK inhibitors (SB203580 and SB202190), PKA inhibitor (H89), PKC inhibitors (Ro31-8220, BMI1, and Go6976), JNK inhibitor (SP600125), and tyrosine kinase inhibitor for JAK2 and EGFR (AG490), affected gene expression in the same direction as the knockdown of CRTC1-MAML2 fusion (Fig. 3d). For instance, the MAPK inhibitor U0126 was identified to cause down-regulation of multiple fusion target genes (Fig. 3e), an effect similar to CRTC1-MAML2 knockdown. Therefore, our data strongly suggest that these biologic drugs and small molecule inhibitors might be effective in inhibiting CRTC1-MAML2 fusion functional activity and blocking MEC growth.

### Identification of CREB-dependent CRTC1-MAML2-regulated genes

We previously showed that the CRTC1-MAML2 fusion interacts with the transcription factor CREB through the CRTC1 CREB binding domain (CBD), and constitutively activates CREB-mediated transcription via the MAML2 transcription activation domain (TAD) [16]. Moreover, CRTC1-MAML2 was able to interact with AP1 [20] and MYC [21]. To determine the extent to which CRTC1-MAML2 induces the specific transcriptional program in human MEC cells through the CREB transcription factor, we evaluated the contribution of CREB in CRTC1-MAML2 regulation of target gene expression.

**Fig. 4:**
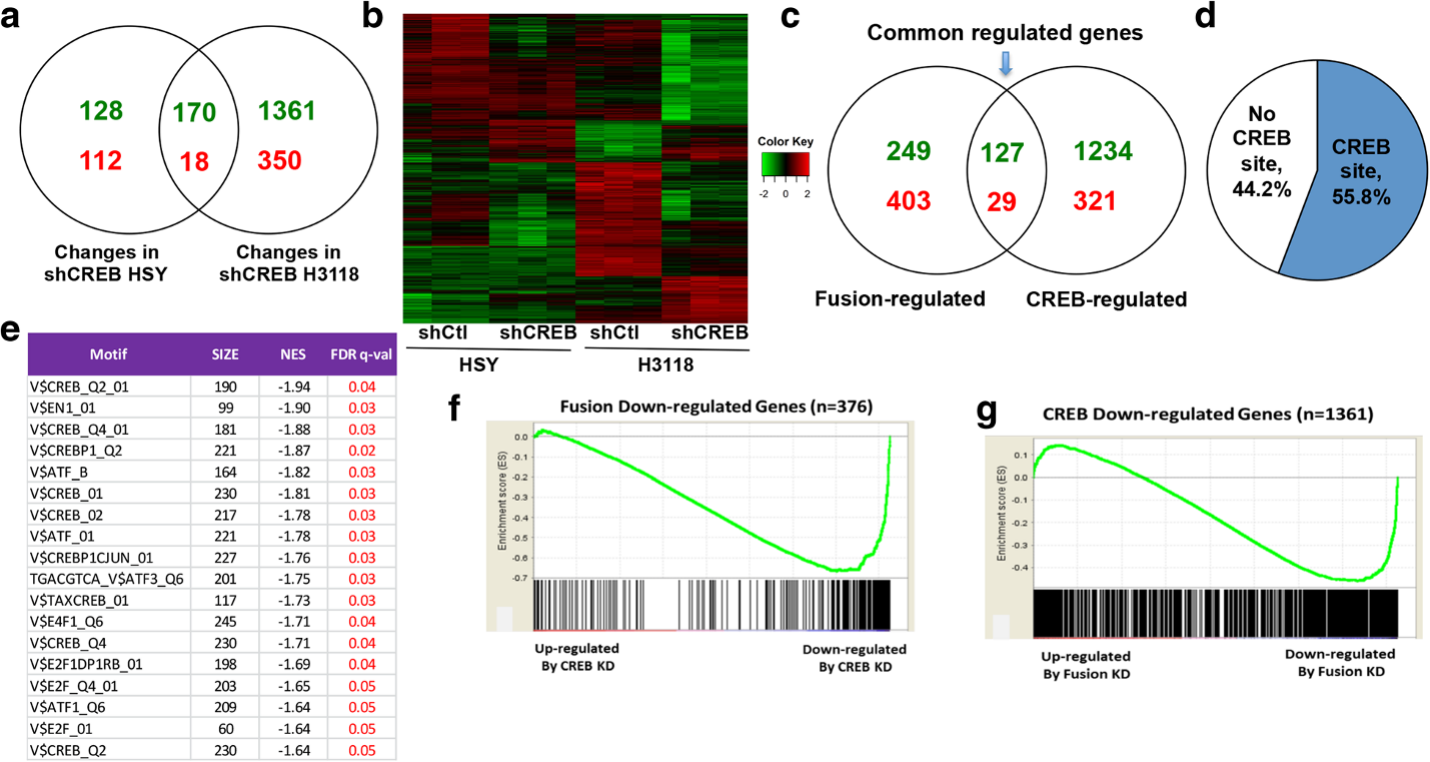
Transcriptional profiling analysis revealed a major action of CRTC1-MAML2 fusion in co-activating CREB target genes in human MEC cells. **a** Venn Diagram indicated that CREB knockdown in fusion-negative HSY and fusion-positive H3118 cells caused gene expression changes in distinct and common genes. Down-regulated and up-regulated genes were shown in green and red. **b** A heatmap of differential expressed genes in HSY and H3118 cells after shCREB transduction. Three biological replicates for each group were included in the analysis. **c** Venn Diagram indicated distinct and overlapping genes between fusion knockdown and CREB knockdown in H3118 cells. **d** A significant subset of CRTC1-MAML2 and CREB common-regulated genes showed CREB binding sites on their promoters. Comparison of the fusion/CREB-regulated genes and the gene set with CREB binding site in the promoter revealed 55.8 % of the fusion/CREB target genes contain CREB binding site. **e** Top 20 transcription factors whose target gene sets were enriched in down-regulated fusion knockdown H3118 array. Each gene set contains genes that shared a transcription factor-binding site defined in the TRANSFAC (version 7.4) database. “Size” represents the number of genes in each data set, “NES” the normalized enrichment score calculated by the GSEA, and the “FDR q-val” is error adjusted false discover rate. CREB-related transcription binding motifs were dominated. **f** GSEA plot indicates that genes down-regulated by fusion knockdown were over-represented at the right of the entire ranked list, which represent the down-regulated genes caused by CREB knockdown (NES −2.548 and FDR q-value < 0.0001). The solid bars represent each individual gene in fusion down-regulated gene set. **g** GSEA plot indicates that CREB down-regulated genes were over-represented at the right (down-regulated by Fusion KD) of the entire ranked list (NES −2.307 and FDR q-value < 0.0001). The solid bars represent each individual gene in fusion down-regulated gene set

We hypothesized that genes controlled by functional interaction of CRTC1-MAML2 fusion and CREB would be down regulated in response to either CRTC1-MAML2 or CREB depletion. Therefore, we determined target genes specifically regulated by CREB in fusion-positive MEC by comparing the impact of CREB knockdown on the gene expression patterns of fusion-positive H3118 MEC cells as well as fusion-negative HSY cells. Here, lentiviruses expressing CREB shRNA (shCREB) and scramble shRNA control (shCtl) were used to infect fusion-negative HSY cells and fusion-positive H3118 cells. RNA samples were prepared from 3 biological replicates at 72 h after viral infection. Gene expression profiling analyses were performed with Affymetrix GeneChip HG-U133 plus 2.0 arrays. Using an absolute fold change of gene expression of > = 2.0 and a p value of < 0.05 as a cutoff, we identified 298 down-regulated and 130 up-regulated genes in fusion-negative HSY cells and 1531 down-regulated and 368 up-regulated genes in fusion-positive H3118 cells after CREB depletion (Fig. 4a, Additional file 1: Figure S3). Comparison of CREB-regulated targets in HSY and H3118 cells showed common and distinct CREB targets in both cell lines. The list of differentially regulated genes affected by CREB knockdown in fusion-positive H3118 cells but not in fusion-negative cells was shown in Additional file 2: Table S2. The heatmap and volcano plots showing changes in both HSY and H3118 cells before and after CREB knockdown were shown in Fig. 4b and Additional file 1: Figure S3.

We next compared CRTC1-MAML2-regulated and CREB-regulated gene lists in H3118 cells, and found a significant overlapping group of genes in fusion-positive MEC H3118 cells with 127 down-regulated and 29 up-regulated genes (Fig. 4c; Additional file 2: Table S3), which suggested the co-regulation of these genes by CRTC1-MAML2 and CREB interaction. To determine whether they were potential direct CREB targets, we compared this group of CRTC1-MAML2/CREB target genes with a dataset that collected a total of 15,784 RefSeq genes with predicted CREs (cAMP-responsive elements; CREB binding site) and 3,666 of them with conserved full and half CREB sites within −3 kb to 300 bp from TSS [29]. We found that 55.8 % of these genes contain the predicted CRE binding sites and 34.5 % of them contain conserved CRE sites (Fig. 4d), supporting that they are potential direct CREB targets. These data strongly support that a major action of CRTC1-MAML2 in activating gene expression is through CREB.

### Gene set enrichment analysis (GSEA) analyses further demonstrate the interaction of CRTC1-MAML2 fusion and CREB in human MEC cells

To further evaluate our hypothesis that a significant set of genes are regulated by the CRTC1-MAML2/CREB interaction, we performed GSEA to determine any correlation of CRTC1-MAML2-regulated or CREB-regulated genes in H3118 cells. GSEA is computational approach that evaluates the distribution of genes in the pre-defined gene sets in the fold change ordered list. The enrichment score (ES) and the weighted Kolmogorov-Smirnov-like statistics indicate whether genes in the pre-defined gene sets are randomly distributed or statistically significantly correlated with phenotypic states (i.e. knockdown vs. control). We first analyzed the C3 TF motif gene set collection from the MsigDB (version 3.1) that contains gene sets annotated as transcription factor targets using the TRANFAC database. We found that CREB-related transcription binding motifs dominated the top 20 transcription factor target gene sets which were enriched in down-regulated genes in fusion-knockdown H3118 cells (Fig. 4e). This data further suggest a major mode of action of CRTC1-MAML2 in transcriptional activation is mediated by CREB. We then used up- or down-regulated genes in fusion knockdown cells (Additional file 2: Table S1) and up- or down-regulated genes in CREB knockdown cells (Additional file 2: Table S2) as pre-defined gene sets for GSEA analysis. We observed concordant enrichment between fusion knockdown-induced down-regulated genes and CREB knockdown-induced down-regulated genes (Fig. 4f, g). Therefore, genes down-regulated after CREB depletion is generally down regulated after fusion depletion, and vice versa, strongly supporting that the CRTC1-MAML2 fusion interacts with CREB to positively regulate a significant percentage of its direct target genes in MEC. It should be noted that there was another significant portion of fusion target genes that were not overlapping with CREB target genes, suggesting that CRTC1-MAML2 also acts through other CREB-independent transcription factors in mediating its oncogenic functions.

## Discussion

Currently diagnostic markers and new effective therapeutic targets for human MEC tumors remain to be identified. The CRTC1-MAML2 fusion gene is highly associated with human MEC and is implicated in MEC tumorigenesis and maintenance. Therefore, clinical improvements for patients with MEC would require a better understanding of CRTC1-MAML2 regarding its altered signaling and molecular actions. In this study, we identified CRTC1-MAML2-regulated transcriptional program in human MEC cells through expressional profiling. Our data suggest that CRTC1-MAML2 mediates its oncogenic functions in CREB-dependent and –independent manners.

To identify CRTC1-MAML2-induced transcriptional profiles, we initially took a subtractive approach that compared gene expression profiles of fusion–positive H3118 cells depleted with both CRTC1-MAML2 fusion and MAML2 and of fusion-negative HSY cells depleted with MAML2 only (Fig. 1b). It should be noted that there were limitations with this approach, as MAML2-regulated target genes in MEC H3118 cells might be different from those in HSY cells. The optimal approach will be to profile and compare the same MEC cells that are depleted of both the CRTC1-MAML2 fusion and MAML2, and MAML2 only. Therefore, the list of CRTC1-MAML2-regulated target candidates should be further validated in the future to ensure that they are indeed targets for CRTC1-MAML2, but not MAML2. However, we think our approach is valuable in providing a list of relevant target genes for further analysis. First, MAML2 expression is very low as compared to the CRTC1-MAML2 fusion in human MEC cells (Fig. 2b). Therefore, MAML2 depletion might not cause profound expression changes and cellular functions. Consistent with this, MAML2 depletion alone did not significantly affect cell proliferation and survival in human MEC cells [18]. Also, there were fewer MAML2 regulated target genes (Fig. 1c) and only two MAML2-regulated genes were found in the fusion/MAML2 gene list (Fig. 1c). Second, when we performed gene validation, we were able to obtain shRNA lentiviruses that specifically targeted both fusion and MAML2, or MAML2 only in the same fusion-positive MEC cells (Fig. 2). We validated all of the 10 target genes in MEC H3118 cells, which is consistent with the microarray data from H3118 cells. Therefore, we think the approach we took is feasible to identify relevant fusion target genes in H3118 cells. Using a similar validation strategy through gene expression analysis of isogenic cells, we confirmed 6 out of these 10 CRTC1-MAML2 regulated genes in a second MEC cell line, human lung MEC H292. These data suggest that there is a common CRTC1-MAML2-regulated transcriptional program in various MEC cancer cells. However, MEC tumors arising from different organs might have different target genes beside the common core transcriptional targets due to the potential interaction of CRTC1-MAML2 fusion with cell type-specific factors, and subsequently could manifest different biological behaviors. Therefore, the CRTC1-MAML2 fusion-regulated transcriptional program in human MEC H3118 that we identified will serve as a foundation for future in-depth analyses of bona-fide genes and pathways regulated by CRTC1-MAML2 fusion. Currently fusion-associated gene signatures have not been evaluated in human MEC tumors. Our fusion-regulated target candidates in H3118 cells will provide important information for the identification of gene signatures associated with fusion-positive tumors, which will be useful for accurate diagnosis of tumors with CRTC1-MAML2 activity as well as evaluation and assignment of specific therapeutic intervention.

To gain biological insights into the CRTC1-MAML2 oncogenic activity, we subsequently performed canonical pathway enrichment analysis and the derivation of mechanistic networks using The Ingenuity Pathways Analysis (IPA) tool. The data strongly suggest that CRTC1-MAML2 fusion have critical functions associated with cell proliferation, growth, survival, movement, metabolism and cell signaling, which can be further investigated. Moreover, IPA upstream regulator analysis identified small molecule inhibitors and biologic drugs that were predicted to cause gene signature changes that were similar to CRTC1-MAML2 fusion gene knockdown, suggesting that those small molecular inhibitors or biologic drugs likely block the fusion oncogenic functions. We found that the MAPK inhibitor U0126, similar to CRTC1-MAML2 knockdown, caused down-regulation of multiple fusion target genes (Fig. 3e). This data is consistent with our recent study demonstrating that CRTC1-MAML2 fusion induced expression of an EGFR ligand AREG, which acted in an autocrine fashion and bound to functional EGFR on human MEC cells to initiate EGFR signaling and activate MAPK signaling. Activated MAPK signaling was demonstrated in human MEC cells by Western blotting analysis for p-Erk in human MEC xenografts, and primary human MEC tumors [18, 30]. The blockade of this pathway via EGFR blocking antibodies inhibited MAPK signaling and inhibited MEC growth [18]. Therefore, MAPK inhibitors could block fusion-induced MAPK signaling and might be an effective way to block MEC tumors. Future studies include determining whether blockade of the critical downstream pathways, individually or in combination, with the predicted drugs or biologics will affect MEC tumor growth. These studies will help to identify effective approaches to treat MEC.

Mechanistically, how the CRTC1-MAML2 fusion oncoprotein induces its transcriptional program is not fully understood. Previous evidence supports a model that the CRTC1 CBD of CRTC1-MAML2 fusion interacts with the transcription factor CREB and its MAML2 TAD co-activates CREB-mediated transcription [8, 16]. CREB activity was required for the transformation of the CRTC1-MAML2 fusion *in vitro* and MEC cell growth, strongly indicating an important role for CREB in mediating CRTC1-MAML2 oncogenic functions. In this study, we further evaluated the contribution of CREB transcription factor in mediating CRTC1-MAML2 transcriptional response in human MEC H3118 cells, and identified a significant overlap between CRTC1-MAML2 and CREB target genes. These data support that CRTC1-MAML2 co-activation of CREB-mediated transcription is a major mechanism for CRTC1-MAML2 oncogenic function. We also found that more than half of these genes contain predicted CRE binding sites. Whether these genes are indeed directly regulated by CRTC1-MAML2 and CREB interaction remain to be addressed. One approach would be sequential chromatin immunoprecipitation for CRTC1-MAML2 and CREB followed by sequencing analysis.

We also identified CRTC1-MAML2 fusion target candidates that appear not to be regulated by CREB, strongly suggesting that CRTC1-MAML2 also induces its transcriptional program in a CREB-independent manner. CRTC1-MAML2 was previously found to display CREB-independent activities via interacting and co-activating AP-1 [20] and MYC [21], both important for CRTC1-MAML2 fusion transformation. Our analysis of the fusion-regulated transcriptional program supports the reported interactions of CRTC1-MAML2 fusion with MYC and AP-1 but also suggest that fusion interacts with other transcription regulators such as NF-κB complex, TP53, E2F1, HIF1A, ATF2, GLI1, NF-AT, TP63, KLF4, IRF6, STAT6, and FOXL2. Therefore, our analyses suggest novel mechanisms of action of CRTC1-MAML2 fusion to interact with transcription factors besides CREB in mediating its oncogenic functions. Future proteomic analysis of endogenous CRTC1-MAML2 fusion protein complexes and functional characterization of the protein complex components in human MEC cancer cells will provide insights into the mechanism of CRTC1-MAML2 fusion oncoprotein and reveal the novel regulators.

## Conclusions

This study is the first to identify specific transcriptional program associated with CRTC1-MAML2, a major oncogene driver in human MEC. Our data provided critical downstream cellular factors/pathways and potential molecular mechanisms of the CRTC1-MAML2 oncoprotein. This study will provide important information for accurate diagnosis for fusion-positive MEC and effective targeted therapeutic treatment.

## Additional files

Additional file 1: Figure S1. Volcano plots showed differential expression between MAML2 TAD shRNA and control groups in fusion-negative HSY cells (A) and in fusion-positive H3118 cells (B). The vertical lines corresponded to 2.0-fold up and down, respectively, and the horizontal line represented a p-value of 0.05. NC denoted no change. “Down” or “Up” referred to significantly down-regulated or upregulated genes. Several fusion-regulated genes in H3118 cells were indicated. **Figure S2.** Validation of a subset of CRTC1-MAML2 fusion-regulated genes in human MEC H292 cell line. Real-time RT-PCR assays were performed in H292 cells that were depleted of fusion/MAML2 or MAML2 only for a subset of CRTC1-MAML2 fusion candidate genes identified in human MEC H3118 cells. **Figure S3.** Volcano plots showed differential expression between CREB shRNA and control groups in fusion-negative HSY cells (A) and in fusion-positive H3118 cells (B). The vertical lines corresponded to 2.0-fold up and down, respectively, and the horizontal line represented a p-value of 0.05. NC denoted no change. “Down” or “Up” referred to significantly down-regulated or up-regulated genes. (PDF 4339 kb)
Additional file 2: Table S1. A list of CRTC1-MAML2 fusion-regulated genes in fusion-positive H3118 MEC cells. Gene expression profiling analyses were performed on fusion/MAML2-knockdown H3118 cells and MAML2-knockdown HSY cells in comparison with their corresponding control cells. Differentially expressed genes with absolute fold change > = 2 and *p* < 0.05 were identified. The differentially expressed genes in fusion/MAML2 knockdown H3118 cells showing the same regulated direction in MAML2 knockdown HSY cells were filtered out. The “positive” and “negative” signs denote upregulated or down-regulated genes in KD compared to control groups, respectively. The asterisk indicates the common gene in different regulatory direction between HSY and H3118. **Table S2.** A list of differentially regulated genes affected by CREB depletion in human fusion-positive MEC, but not fusion-negative cells. The differentially expressed genes after CREB was depleted were identified in both fusion-positive MEC H3118 cells and fusion-negative HSY cells. Those CREB-regulated genes in H3118 cells showing the same regulated direction in fusion-negative HSY were then filtered out. The “positive” and “negative” signs denote up-regulated or down-regulated genes in KD compared to control groups, respectively. The asterisk indicates the common gene in different regulatory direction between HSY and H3118 cells. **Table S3.** A list of common differentially expressed genes affected by CRTC1-MAML2- or CREB-depletion in human MEC H3118 cells. This gene list represents candidate genes regulated by fusion/CREB interaction. The “positive” and “negative” signs denote up-regulated or down-regulated genes in KD compared to control groups, respectively. The asterisk indicates the common gene in different regulatory direction between fusion and CREB. (PDF 707 kb)
