# Supplementary material for: Gene expression profiling analysis of CRTC1-MAML2 fusion oncogene-induced transcriptional program in human mucoepidermoid carcinoma cells
Source: BMC Cancer. 2015 Oct 26;15:803. doi: 10.1186/s12885-015-1827-3 (PMC4624166; doi:10.1186/s12885-015-1827-3)
Supplement: Additional file 1: Figure S1. — Volcano plots showed differential expression between MAML2 TAD shRNA and control groups in fusion-negative HSY cells (A) and in fusion-positive H3118 cells (B). The vertical lines corresponded to 2.0-fold up and down, respectively, and the horizontal line represented a p-value of 0.05. NC denoted no change. “Down” or “Up” referred to significantly down-regulated or upregulated genes. Several fusion-regulated genes in H3118 cells were indicated. Figure S2. Validation of a subset of CRTC1-MAML2 fusion-regulated genes in human MEC H292 cell line. Real-time RT-PCR assays were performed in H292 cells that were depleted of fusion/MAML2 or MAML2 only for a subset of CRTC1-MAML2 fusion candidate genes identified in human MEC H3118 cells. Figure S3. Volcano plots showed differential expression between CREB shRNA and control groups in fusion-negative HSY cells (A) and in fusion-positive H3118 cells (B). The vertical lines corresponded to 2.0-fold up and down, respectively, and the horizontal line represented a p-value of 0.05. NC denoted no change. “Down” or “Up” referred to significantly down-regulated or up-regulated genes. (PDF 4339 kb) [file 12885_2015_1827_MOESM1_ESM.pdf]

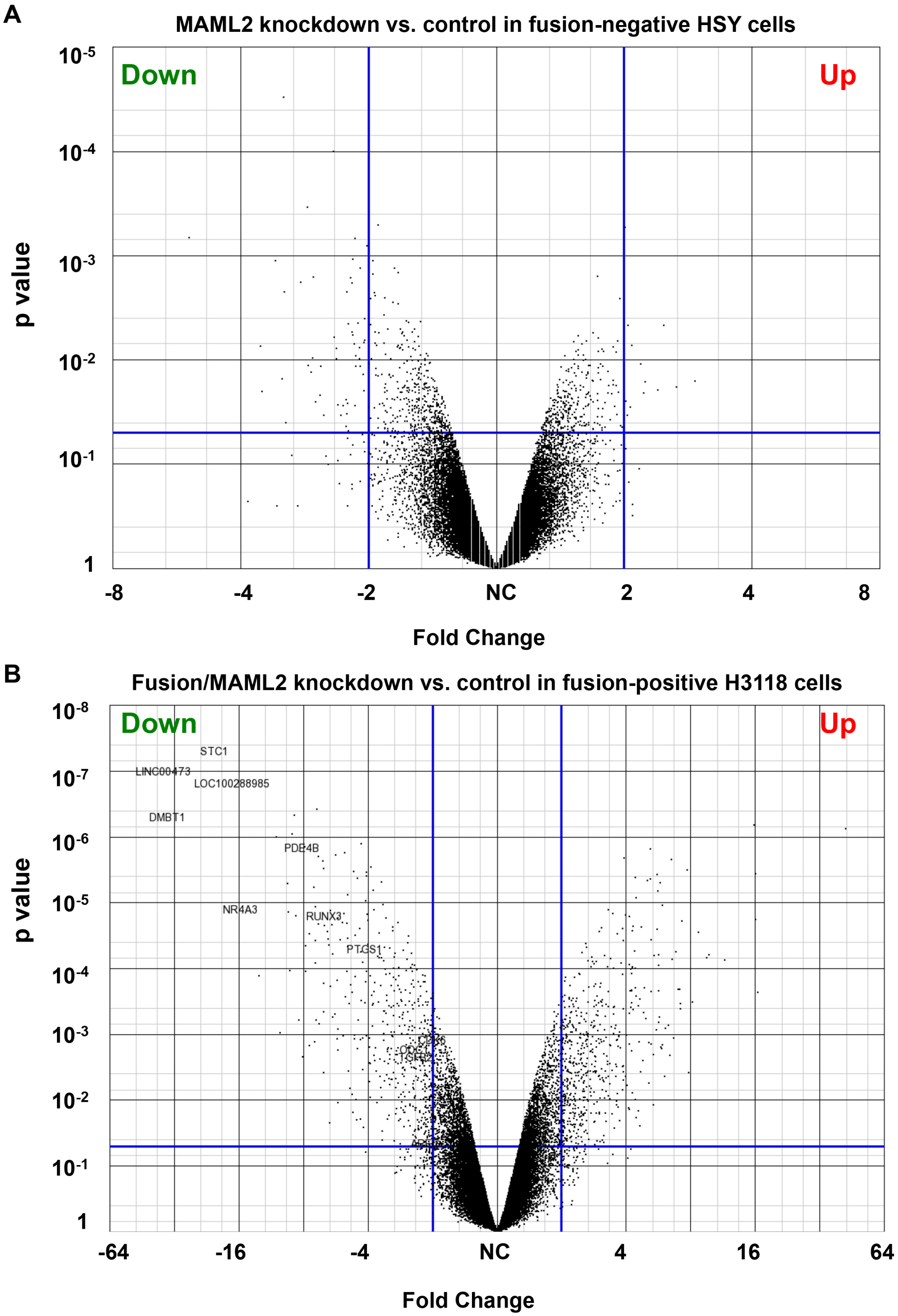

Figure S1. Volcano plots showed differential expression between MAML2 TAD shRNA and control groups in fusion-negative HSY cells (A) and in fusion-positive H3118 cells (B). The vertical lines corresponded to 2.0-fold up and down, respectively, and the horizontal line represented a p-value of 0.05. NC denoted no change. “Down” or “ Up” referred to significantly down-regulated or up-regulated genes. Several fusion-regulated genes in H3118 cells were indicated.

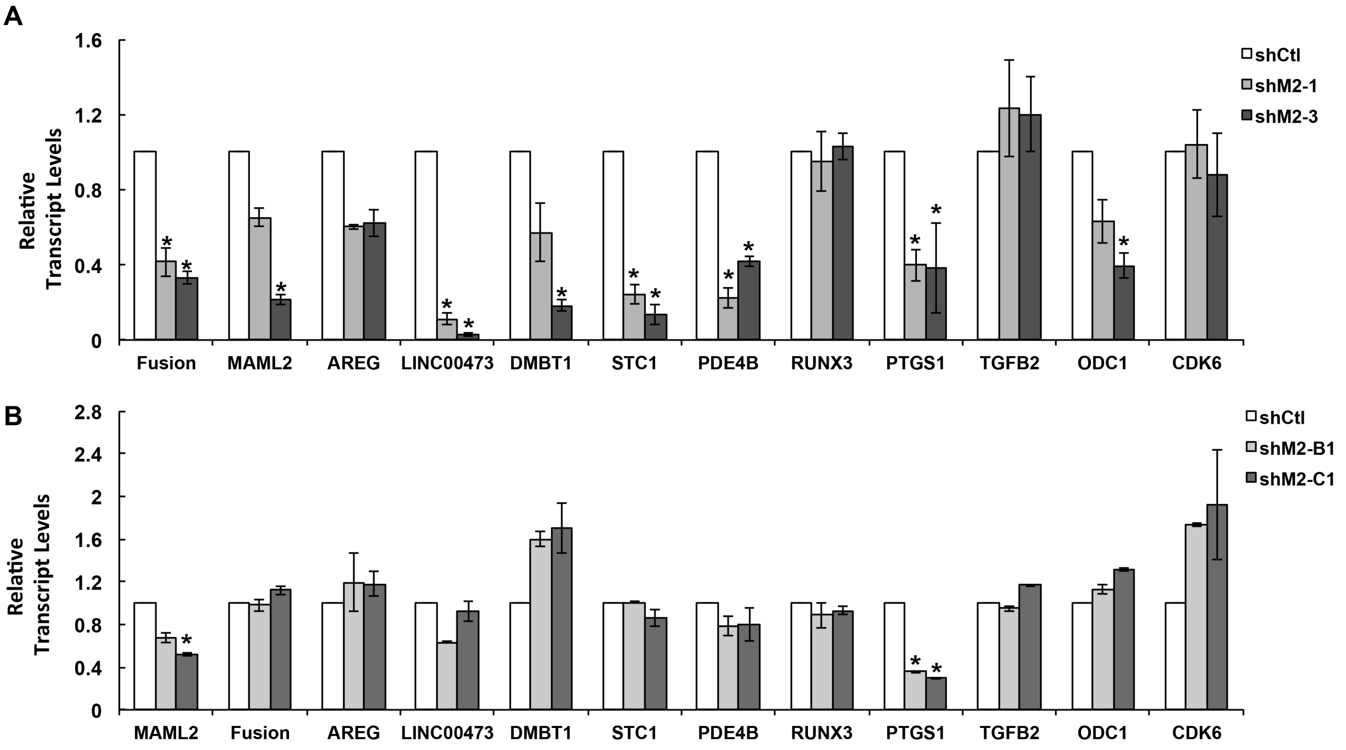

Figure S2. Validation of a subset of CRTC1-MAML2 fusion-regulated genes in human MEC H292 cell line. Real-time RT-PCR assays were performed in H292 cells that were depleted of fusion/MAML2 or MAML2 only for a subset of CRTC1-MAML2 fusion candidate genes identified in human MEC H3118 cells.

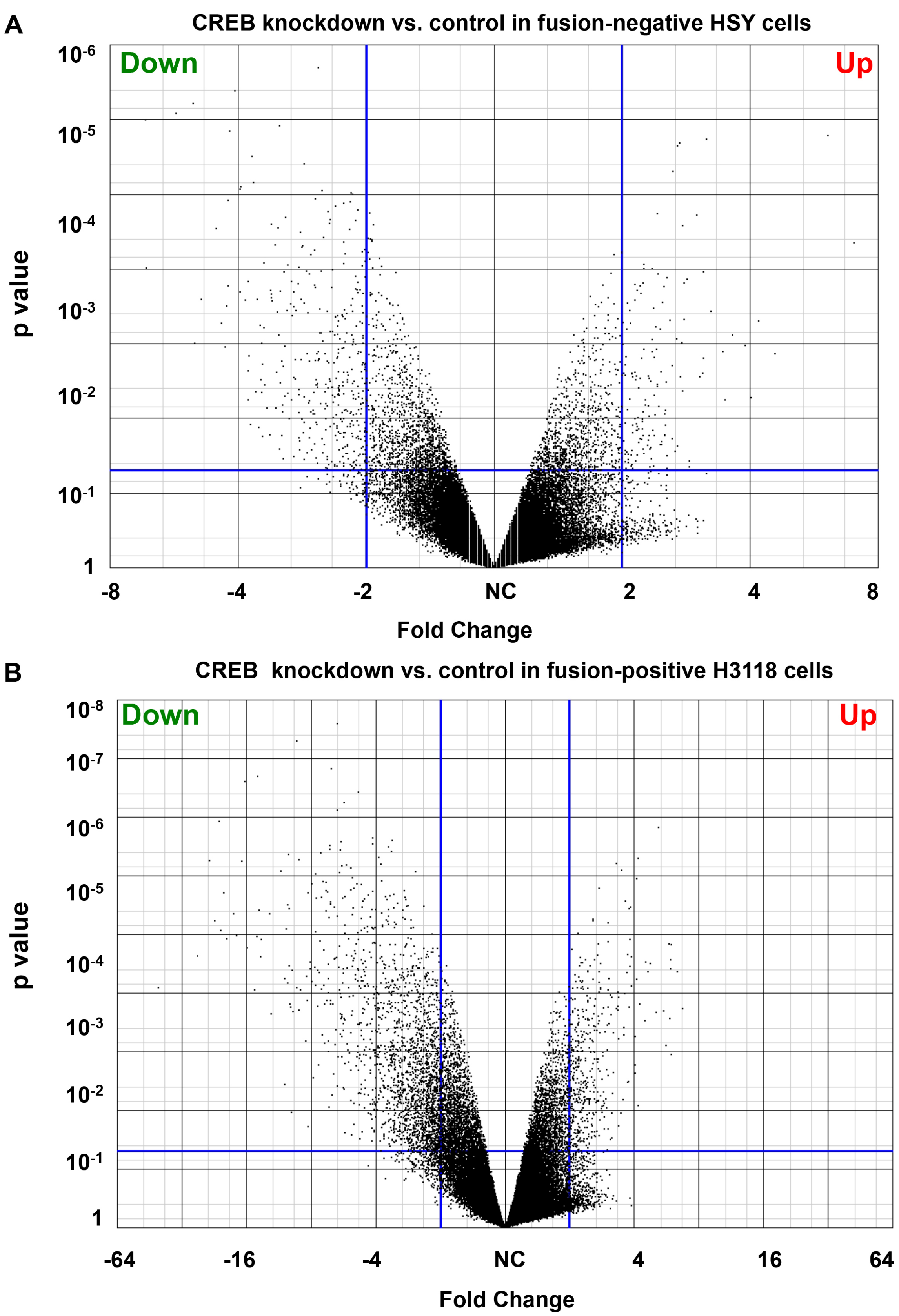

Figure S3. **Volcano plots showed differential expression between CREB shRNA and control groups in fusion-negative HSY cells (A) and in fusion-positive H3118 cells (B).** The vertical lines corresponded to 2.0-fold up and down, respectively, and the horizontal line represented a p-value of 0.05. NC denoted no change. " Down" or " Up" referred to significantly down-regulated or up-regulated genes.
