# Supplementary material for: Gene expression profiling analysis of CRTC1-MAML2 fusion oncogene-induced transcriptional program in human mucoepidermoid carcinoma cells
Source: BMC Cancer. 2015 Oct 26;15:803. doi: 10.1186/s12885-015-1827-3 (PMC4624166; doi:10.1186/s12885-015-1827-3)
Supplement: Additional file 2: Table S1. — A list of CRTC1-MAML2 fusion-regulated genes in fusion-positive H3118 MEC cells. Gene expression profiling analyses were performed on fusion/MAML2-knockdown H3118 cells and MAML2-knockdown HSY cells in comparison with their corresponding control cells. Differentially expressed genes with absolute fold change > = 2 and p < 0.05 were identified. The differentially expressed genes in fusion/MAML2 knockdown H3118 cells showing the same regulated direction in MAML2 knockdown HSY cells were filtered out. The “positive” and “negative” signs denote upregulated or down-regulated genes in KD compared to control groups, respectively. The asterisk indicates the common gene in different regulatory direction between HSY and H3118. Table S2. A list of differentially regulated genes affected by CREB depletion in human fusion-positive MEC, but not fusion-negative cells. The differentially expressed genes after CREB was depleted were identified in both fusion-positive MEC H3118 cells and fusion-negative HSY cells. Those CREB-regulated genes in H3118 cells showing the same regulated direction in fusion-negative HSY were then filtered out. The “positive” and “negative” signs denote up-regulated or down-regulated genes in KD compared to control groups, respectively. The asterisk indicates the common gene in different regulatory direction between HSY and H3118 cells. Table S3. A list of common differentially expressed genes affected by CRTC1-MAML2- or CREB-depletion in human MEC H3118 cells. This gene list represents candidate genes regulated by fusion/CREB interaction. The “positive” and “negative” signs denote up-regulated or down-regulated genes in KD compared to control groups, respectively. The asterisk indicates the common gene in different regulatory direction between fusion and CREB. (PDF 707 kb) [file 12885_2015_1827_MOESM2_ESM.pdf]

Table S1: A list of CRTC1-MAML2 fusion-regulated genes in fusion-positive H3118 MEC cells. Gene expression profiling analyses were performed on fusion/MAML2-knockdown H3118 cells and MAML2-knockdown HSY cells in comparison with their corresponding control cells. Differentially expressed genes with absolute fold change >= 2 and p<0.05 were identified. The differentially expressed genes in fusion/MAML2 knockdown H3118 cells showing the same regulated direction in MAML2 knockdown HSY cells were filtered out. The "positive" and "negative" signs denote up-regulated or down-regulated genes in KD compared to control groups, respectively. The asterisk indicates the common gene in different regulatory direction between HSY and H3118.

Gene Symbol	Gene Title	Fold change Fusion&MAML2 KD/Control (H3118)	p-value (H3118)	Fold change MAML2 KD/Control (HSY)	p-value (HSY)
**LINC00473**	chromosome 6 open reading frame 176	-37.12	0	-1.03	0.87
**DMBT1**	deleted in malignant brain tumors 1	-35.73	0	1.16	0.52
**STC1**	stanniocalcin 1	-21.74	0	1.03	0.83
**LOC100288985**	Hypothetical protein LOC100288985	-17.73	0	1.01	0.96
**NR4A3**	nuclear receptor subfamily 4, group A, member 3	-16.29	1.00E-05	1.02	0.95
**AW302848**	---	-12.93	0.00013	-1.33	0.43432
**ATP2B2**	ATPase, Ca++ transporting, plasma membrane 2	-10.68	0	-1.1	0.56
**KIAA1199**	KIAA1199	-10.29	0.00094	-1.09	0.85
**OSBP2**	oxysterol binding protein 2	-9.52	1.00E-05	1.04	0.84
**SLC8A1**	solute carrier family 8 (sodium/calcium exchanger), member 1	-9.25	0.00011	-1.29	0.41
**CAPN14**	calpain 14	-9.04	0	-1.13	0.43
**PLEKHH1**	pleckstrin homology domain containing, family H (with MyTH4 domain) member 1	-8.80	0	-1.15	0.30
**NR4A2**	nuclear receptor subfamily 4, group A, member 2	-8.67	2.00E-05	-1.05	0.82
**SORBS2**	sorbin and SH3 domain containing 2	-8.42	6.00E-04	-1.22	0.60
**PDE4B**	phosphodiesterase 4B, cAMP-specific	-8.41	0	1.09	0.58
**C2CD4A**	C2 calcium-dependent domain containing 4A	-7.84	0.00011	-1.03	0.91
**ARG2**	arginase, type II	-7.79	5.00E-05	1.1	0.70
**AL355689**	---	-7.04	8.00E-05	-1.18	0.51225
**FAM176C**	family with sequence similarity 176, member (	-7.01	1.00E-05	-1	1.00
**LUM**	lumican	-6.98	0.00061	1.19	0.63
**PECAM1**	platelet/endothelial cell adhesion molecule	-6.92	0	2.01	0.00
**UBE2QL1**	ubiquitin-conjugating enzyme E2Q family-like 1	-6.81	0	-1.04	0.79
**HAL**	histidine ammonia-lyase	-6.79	2.00E-05	1.28	0.25
**AGXT2L1**	alanine-glyoxylate aminotransferase 2-like 1	-6.76	0.00068	1.17	0.65
**SMOC1**	SPARC related modular calcium binding 1	-6.47	0	-1.01	0.93
**BC027448**	---	-6.45	0.00045	-1.15	0.65648
**BC020911**	---	-6.44	0.00377	1.5	0.38979
**PPP2R1B**	protein phosphatase 2, regulatory subunit A, beta	-6.44	0	-1.22	0.20
**NR4A1**	nuclear receptor subfamily 4, group A, member 1	-6.14	4.00E-05	1.05	0.80
**T79639**	---	-6.06	0.02185	-1.15	0.8231
**AA523939**	---	-6.00	1.00E-05	-1.01	0.9645

**NPR3**	natriuretic peptide receptor C/guanylate cycla:	-6.00	0.00019	1.02	0.93
**CREG2**	cellular repressor of E1A-stimulated genes 2	-5.91	2.00E-05	-1.08	0.69
**LSAMP**	limbic system-associated membrane protein	-5.83	6.00E-05	-1.34	0.21
**PCSK1**	proprotein convertase subtilisin/kexin type 1	-5.70	0.00048	1.22	0.52
**RHOBTB2**	Rho-related BTB domain containing 2	-5.68	0	1.06	0.66
**N29850**	---	-5.55	0.00118	-1.09	0.801
**CHL1-AS2**	CHL1 antisense RNA 2	-5.54	0.00083	-1.15	0.66
**AW960100**	---	-5.52	0.01095	1.06	0.90602
**LGSN**	lengsin, lens protein with glutamine synthetase domain	-5.27	2.00E-05	-1.38	0.10
**GREM1**	gremlin 1	-5.21	0.00105	-1.3	0.43
**AA700206**	---	-5.02	2.00E-05	-1.19	0.31209
**H12214**	---	-4.97	3.00E-05	-1	0.99957
**MFSD2A**	major facilitator superfamily domain containing 2A	-4.96	5.00E-05	-1.01	0.96
**RUNX3**	runt-related transcription factor 3	-4.88	0.00034	1.03	0.92
**AI476341**	---	-4.87	0.00659	-1.03	0.9506
**DUSP1**	dual specificity phosphatase 1	-4.81	0.00017	-1.03	0.89
**AI141861**	---	-4.75	0.00023	1.1	0.69989
**SLC8A1-AS1**	SLC8A1 antisense RNA 1	-4.75	0.00808	1.15	0.75
**AK024800**	---	-4.72	0.01546	-1.02	0.96439
**M31157**	---	-4.64	0.00116	-1.12	0.70413
**A2M**	alpha-2-macroglobulin	-4.62	0.00102	1.53	0.18
**C19orf77**	chromosome 19 open reading frame 77	-4.59	3.00E-05	1.09	0.63
**GFRA1**	GDNF family receptor alpha 1	-4.56	0.00966	-1.37	0.49
**AK022474**	---	-4.51	0.01964	-1.09	0.86726
**CHL1**	Cell adhesion molecule with homology to L1CAM	-4.47	0.00163	-1.43	0.28
**EHD3**	EH-domain containing 3	-4.45	0.00061	1.18	0.54
**ZNF331**	zinc finger protein 331	-4.24	8.00E-05	1.01	0.95
**IL20**	interleukin 20	-4.23	0.00345	-1.19	0.61
**AI452799**	---	-4.20	0.02557	-1.12	0.83286
**ENO3**	enolase 3 (beta, muscle)	-4.14	0.00033	-1.1	0.69
**EOMES**	eomesodermin	-4.10	1.00E-05	1.07	0.62
**AI806183**	---	-4.10	0.00016	1.01	0.96464
**SLAIN1**	SLAIN motif family, member 1	-4.08	0	1.07	0.60
**QRFPR**	pyroglutamylated RFamide peptide receptor	-4.07	0.00011	1	0.99
**SIK2**	salt-inducible kinase 2	-4.06	0	-1.01	0.94
**PNLDC1**	poly(A)-specific ribonuclease (PARN)-like domain containing 1	-4.00	4.00E-05	1.1	0.56
**GREM2**	gremlin 2	-3.95	0.00271	-1.04	0.91
**SRGN**	serglycin	-3.95	0.00518	1.22	0.59
**DUSP4**	dual specificity phosphatase 4	-3.93	0.00155	-1	0.99
**NM_024853**	---	-3.92	0.00013	-1.09	0.6543
**PTGS1**	prostaglandin-endoperoxide synthase 1 (prostaglandin G/H synthase and cyclooxygenase)	-3.89	0	1.31	0.04
**PTHLH**	parathyroid hormone-like hormone	-3.82	0.00035	1.35	0.20
**AV702787**	---	-3.81	0.00075	1.03	0.90859
**FZD10**	frizzled homolog 10 (Drosophila)	-3.79	0.00034	1.1	0.67
**LINC00323**	long intergenic non-protein coding RNA 323	-3.72	0.00249	-1.06	0.84
**WDR66**	WD repeat domain 66	-3.65	0.0036	1.2	0.57
**CREB5**	cAMP responsive element binding protein 5	-3.63	0.01072	-1.18	0.68
**ENOX1**	ecto-NOX disulfide-thiol exchanger 1	-3.59	6.00E-05	1.08	0.65

**BG112263**	---	-3.55	3.00E-05	1.06	0.69217
**TMCO7**	transmembrane and coiled-coil domains 7	-3.54	7.00E-05	1.03	0.84
**AK026914**	---	-3.52	0.00237	-1.14	0.64894
**AK021804**	---	-3.52	0.0043	1.03	0.92584
**TMEM229B**	transmembrane protein 229B	-3.51	6.00E-05	1.05	0.75
**CALCA**	calcitonin-related polypeptide alpha	-3.49	0.00643	1.13	0.72
**AL831884**	---	-3.47	0.00902	1.12	0.75966
**SLCO4A1**	solute carrier organic anion transporter family, member 4A1	-3.46	0	1.02	0.87
**MCAM**	melanoma cell adhesion molecule	-3.45	7.00E-05	1.07	0.68789
**RPS2P45**	Ribosomal protein S2 pseudogene 45	-3.44	0.04081	-1.13	0.81
**MIR100HG**	mir-100-let-7a-2 cluster host gene (non-protei	-3.42	0.00064	1.02	0.92
**AIG1**	androgen-induced 1	-3.42	1.00E-05	1.23	0.11
**N90870**	---	-3.39	0.00122	-1.11	0.66954
**FAM184A**	family with sequence similarity 184, member A	-3.38	8.00E-05	1.45	0.04
**AI343000**	---	-3.38	0.00097	-1.25	0.35792
**CDH11**	cadherin 11, type 2, OB-cadherin (osteoblast)	-3.38	0.00044	-1.07	0.74
**AA699346**	---	-3.36	0.00511	-1.1	0.76413
**IL6**	interleukin 6 (interferon, beta 2)	-3.35	0.00259	1.03	0.92
**MCF2L-AS1**	MCF2L antisense RNA 1	-3.35	0.00045	-1.14	0.53
**NCALD**	neurocalcin delta	-3.34	5.00E-05	1.22	0.19
**PDE10A**	phosphodiesterase 10A	-3.32	1.00E-04	-1.08	0.63
**TECPR2**	tectonin beta-propeller repeat containing 2	-3.31	0.00012	-1.09	0.62
**AL832253**	---	-3.28	0.00031	-1.19	0.38218
**AW138143**	---	-3.26	0.00025	-1.04	0.84646
**AW979271**	---	-3.25	0.01939	-1.12	0.78505
**TNFRSF19**	tumor necrosis factor receptor superfamily, member 19	-3.24	0.00183	1.05	0.84
**GRAMD1B**	GRAM domain containing 1B	-3.24	0.00167	-1.03	0.91
**LOC100506870**	hypothetical LOC100506870	-3.24	0.00071	1.13	0.58
**AL832681**	---	-3.22	0.00278	1.01	0.98174
**SLC2A14**	solute carrier family 2 (facilitated glucose transporter), member 14	-3.20	0.00037	1.03	0.87
**BF508839**	---	-3.19	0.00463	-1.13	0.68957
**LOC283713**	hypothetical protein LOC283713	-3.19	0.01709	-1.32	0.49
**FAM83A**	family with sequence similarity 83, member A	-3.18	0.00238	1.01	0.97
**MSI2**	musashi homolog 2 (Drosophila)	-3.17	0.00012	1.17	0.34
**AA416756**	---	-3.16	0.00578	1.09	0.77465
**HAVCR2**	hepatitis A virus cellular receptor 2	-3.16	5.00E-05	1.06	0.66
**SFRP1**	secreted frizzled-related protein 1	-3.12	0.00018	1.42	0.06
**F2RL2**	coagulation factor II (thrombin) receptor-like 2	-3.11	0.00039	1.28	0.22
**PLD1**	phospholipase D1, phosphatidylcholine-specific	-3.11	0.00164	-1.05	0.82
**FUCA1**	fucosidase, alpha-L- 1, tissue	-3.09	0.0048	1.12	0.71
**MGC39372**	serpin peptidase inhibitor, clade B (ovalbumin), member 9 pseudogene	-3.09	0.00106	1.14	0.55
**AI075770**	---	-3.07	0.00108	-1.17	0.48147
**TPRG1**	tumor protein p63 regulated 1	-3.06	3.00E-05	1.48	0.01
**RAB3A**	RAB3A, member RAS oncogene family	-3.05	0.00377	1.13	0.65

**RASD1**	RAS, dexamethasone-induced 1	-3.05	1.00E-04	1.02	0.89
**LPPR4**	lipid phosphate phosphatase-related protein type 4	-3.04	0.00051	-1.28	0.23
**E2F2**	E2F transcription factor 2	-3.03	0.0024	-1.03	0.90
**STEAP4**	STEAP family member 4	-3.03	0.00022	1.11	0.55
**SERPINB9**	serpin peptidase inhibitor, clade B (ovalbumin), member 9	-2.98	0.00472	1.53	0.16
**FAM111B**	family with sequence similarity 111, member B	-2.97	0.01626	-1.12	0.76
**SYT12**	synaptotagmin XII	-2.97	0.00015	-1.06	0.73
**SMOX**	spermine oxidase	-2.95	0.01324	-1.02	0.95
**AW291714**	---	-2.95	0.00177	-1.05	0.84341
**PDE4D**	phosphodiesterase 4D, cAMP-specific	-2.93	0.00034	1.05	0.79
**MDS2**	myelodysplastic syndrome 2 translocation associated	-2.93	0.002	-1.05	0.83
**ATP6V0A4**	ATPase, H+ transporting, lysosomal V0 subunit a4	-2.90	0.00544	-1.42	0.23
**AI082004**	---	-2.90	0.00275	-1.19	0.48172
**AW138815**	---	-2.90	0.00162	1.13	0.58513
**TNFRSF10B**	tumor necrosis factor receptor superfamily, member 10b	-2.89	0.00018	-1.11	0.50
**XDH**	xanthine dehydrogenase	-2.87	0.02018	-1.33	0.45
**BC037339**	---	-2.86	0.00021	1.09	0.59679
**NFASC**	neurofascin	-2.85	0.00054	1.16	0.43
**AGT**	angiotensinogen (serpin peptidase inhibitor, clade A, member 8)	-2.84	0.01948	1.3	0.48
**LOC338651**	hypothetical LOC338651	-2.82	0.04575	-1.11	0.81
**PTGES**	prostaglandin E synthase	-2.81	0.0132	1.07	0.83
**C1QTNF6**	C1q and tumor necrosis factor related protein 6	-2.81	0.00023	1.02	0.89
**USP13**	ubiquitin specific peptidase 13 (isopeptidase T-3)	-2.80	0.00813	1.09	0.76
**FSIP1**	fibrous sheath interacting protein 1	-2.80	0.00272	-1.06	0.81
**TESC**	tescalcin	-2.80	0.00029	-1.07	0.67
**BICD2**	bicaudal D homolog 2 (Drosophila)	-2.79	0.00096	1.28	0.24
**COL4A3**	collagen, type IV, alpha 3 (Goodpasture antigen)	-2.79	0.00211	-1.12	0.63
**FLJ31485**	hypothetical LOC440119	-2.79	0.01063	-1.09	0.79
**SEMA3B**	sema domain, immunoglobulin domain (Ig), short basic domain, secreted, (semaphorin) 3B	-2.78	0.00031	-1.13	0.45
**CA9**	carbonic anhydrase IX	-2.77	0.00402	1.2	0.47
**XPR1**	xenotropic and polytropic retrovirus receptor 1	-2.77	0.00322	-1.24	0.3881
**AI973099**	---	-2.76	5.00E-04	1.06	0.75676
**CLDN18**	claudin 18	-2.76	0.00058	1.19	0.34
**AA927995**	---	-2.74	0.00047	-1.26	0.20483
**AL833742**	---	-2.74	0.01416	1.12	0.72257
**PON3**	paraoxonase 3	-2.74	0.00314	-1.07	0.78
**RAET1E**	retinoic acid early transcript 1E	-2.72	0.02536	1.21	0.61
**MAGIX**	MAGI family member, X-linked	-2.71	0.00432	1.23	0.43
**SPRY1**	sprouty homolog 1, antagonist of FGF signaling (Drosophila)	-2.70	0.01542	1.17	0.64
**N55072**	---	-2.70	0.00341	-1.16	0.53791
**AFF3**	AF4/FMR2 family, member 3	-2.69	0.00072	-1.07	0.72

**KSR1**	kinase suppressor of ras 1	-2.69	0.00355	1.16	0.53
**ZNF462**	zinc finger protein 462	-2.69	0.00885	1.21	0.51
**AI078033**	---	-2.68	0.00037	-1.17	0.35915
**ULBP2**	UL16 binding protein 2	-2.67	0.00021	-1.04	0.80
**IRX4**	iroquois homeobox 4	-2.67	0.00233	-1.36	0.19
**LOC401317**	hypothetical LOC401317	-2.67	0.01249	-1.03	0.92
**CGA**	glycoprotein hormones, alpha polypeptide	-2.66	0.00011	1.48	0.02
**SLC2A3**	solute carrier family 2 (facilitated glucose transporter), member 3	-2.65	0.00144	1.03	0.88
**MPPED2**	metallophosphoesterase domain containing 2	-2.65	0.00261	-1.35	0.21
**CLMP**	CXADR-like membrane protein	-2.63	7.00E-05	-1.03	0.80
**KYNU**	kynureninase (L-kynurenine hydrolase)	-2.63	0.00098	-1.08	0.70
**BF433037**	---	-2.62	0.00046	-1.12	0.49457
**BF590850**	---	-2.60	4.00E-05	1.13	0.30427
**IGFN1**	immunoglobulin-like and fibronectin type III domain containing 1	-2.60	0.00162	-1.2	0.38
**C20orf132**	chromosome 20 open reading frame 132	-2.58	0.00086	1.14	0.48
**SLC35G2**	solute carrier family 35, member G2	-2.57	0.00077	-1.04	0.81
**DUSP2**	dual specificity phosphatase 2	-2.57	0.00032	1.26	0.16
**BC043169**	---	-2.56	0.00745	1.05	0.85744
**IL13RA2**	interleukin 13 receptor, alpha 2	-2.55	0.01144	1.09	0.76
**GAS1**	growth arrest-specific 1	-2.55	0.00158	1.03	0.90
**AW843302**	---	-2.54	0.02473	-1.15	0.68565
**MPRIP**	myosin phosphatase Rho interacting protein	-2.54	0.00113	1.05	0.80
**ODC1**	ornithine decarboxylase 1	-2.54	0.0019	-1.06	0.79
**PPARGC1A**	peroxisome proliferator-activated receptor gamma, coactivator 1 alpha	-2.54	0.00187	1	0.98
**OTUD7A**	OTU domain containing 7A	-2.54	0.00062	-1.05	0.77
**ANKRD9**	ankyrin repeat domain 9	-2.52	0.00135	-1.01	0.96
**GPR133**	G protein-coupled receptor 133	-2.52	0.00279	-1.11	0.63
**LOC283140**	hypothetical protein LOC283140	-2.52	1.00E-04	-1.09	0.49
**ST6GALNAC3**	ST6 (alpha-N-acetyl-neuraminyl-2,3-beta-galactosyl-1,3)-N-acetylgalactosaminide alpha-2,6-sialyltransferase 3	-2.51	6.00E-05	-1.13	0.29
**ANO3**	anoctamin 3	-2.50	0.00317	1.05	0.83
**PIGH**	phosphatidylinositol glycan anchor biosynthesis, class H	-2.50	0.00017	1.02	0.91
**BF590769**	---	-2.49	0.00036	1.05	0.73378
**NID2**	nidogen 2 (osteonidogen)	-2.49	0.00011	1.18	0.21
**TGFB2**	transforming growth factor, beta 2	-2.48	0.00244	1.21	0.37
**ALDH3B1**	aldehyde dehydrogenase 3 family, member B1	-2.48	9.00E-05	1.11	0.40
**EXT1**	exostosin 1	-2.48	0.00074	1.03	0.88
**ONECUT2**	one cut homeobox 2	-2.47	0.00124	-1.09	0.63
**PSD3**	pleckstrin and Sec7 domain containing 3	-2.47	0.03327	1.26	0.53
**AVPI1**	arginine vasopressin-induced 1	-2.47	0.00015	-1.01	0.95
**FAM43A**	family with sequence similarity 43, member A	-2.47	0.00646	1.01	0.97
**NM_024912**	---	-2.46	0.0057	1.21	0.43952
**EYA1**	eyes absent homolog 1 (Drosophila)	-2.46	0.00041	1.27	0.14
**AI289774**	---	-2.46	0.01229	-1.08	0.78157
**RASSF10**	Ras association (RalGDS/AF-6) domain family (N-terminal) member 10	-2.45	0.00684	-1.34	0.26

**AW500340**	---	-2.45	0.00511	1.43	0.15117
**LOC100506834**	hypothetical LOC100506834	-2.45	0.00317	-1.14	0.55
**LOC100128653**	hypothetical LOC100128653	-2.44	0.00019	1.06	0.64
**TSPAN8**	tetraspanin 8	-2.43	0.0296	-1	1.00
**CPVL**	carboxypeptidase, vitellogenic-like	-2.43	0.00902	1.09	0.74
**FEZ1**	fasciculation and elongation protein zeta 1 (zygin I)	-2.42	9.00E-05	1.03	0.77
**DZIP1L**	DAZ interacting protein 1-like	-2.42	0.00033	1.16	0.32
**SSH1**	slingshot homolog 1 (Drosophila)	-2.42	0.00079	-1	0.98
**BF751607**	---	-2.41	0.01425	1.17	0.57802
**PTGS2**	prostaglandin-endoperoxide synthase 2 (prostaglandin G/H synthase and cyclooxygenase)	-2.41	0.02817	-1.04	0.90
**ATP8B4**	ATPase, class I, type 8B, member 4	-2.41	0.00865	1.3	0.32
**CENPV**	centromere protein V	-2.40	0.00211	-1.03	0.89
**KLF9**	Kruppel-like factor 9	-2.40	0.00169	1.24	0.26
**PDK3**	pyruvate dehydrogenase kinase, isozyme 3	-2.40	0.03499	1.06	0.86
**RNF157-AS1**	RNF157 antisense RNA 1	-2.40	0.01288	1.04	0.89863
**AI761207**	---	-2.39	0.00236	1.05	0.79811
**SOBP**	sine oculis binding protein homolog (Drosophila)	-2.38	0.03683	1.1	0.78
**AL833050**	---	-2.38	0.04945	-1.07	0.86593
**BMP8B**	bone morphogenetic protein 8b	-2.38	0.00438	1.1	0.66
**H79303**	---	-2.38	0.04488	-1.06	0.87769
**CD55**	CD55 molecule, decay accelerating factor for complement (Cromer blood group)	-2.37	1.00E-04	-1	0.97
**BE219187**	---	-2.36	0.04447	-1.08	0.82833
**SNX25**	sorting nexin 25	-2.36	0.01993	-1.08	0.79
**WDR76**	WD repeat domain 76	-2.35	0.00499	1.03	0.89
**MKX**	mohawk homeobox	-2.35	0.00244	-1.03	0.89
**BE271180**	---	-2.34	0.00459	1.1	0.6514
**NFIA**	Nuclear factor I/A	-2.34	0.02628	1.04	0.90
**AL038725**	---	-2.34	0.028	-1.09	0.79988
**BCAR3**	breast cancer anti-estrogen resistance 3	-2.34	0.00029	1.01	0.92
**PCK1**	phosphoenolpyruvate carboxykinase 1 (soluble)	-2.34	0.02471	1.71	0.12
**CASP9**	caspase 9, apoptosis-related cysteine peptidase	-2.33	0.00016	1	0.99
**RND2**	Rho family GTPase 2	-2.32	0.00173	1.37	0.11
**EPYC**	epiphycan	-2.31	0.00142	1.16	0.39
**C11orf86**	chromosome 11 open reading frame 86	-2.31	0.00125	1.07	0.68
**ZNF367**	zinc finger protein 367	-2.31	0.00111	1.02	0.92
**HDAC5**	histone deacetylase 5	-2.30	0.00216	1	0.99
**TRIM2**	tripartite motif-containing 2	-2.30	0.00157	1.02	0.89
**LOC728431**	hypothetical LOC728431	-2.30	0.00174	-1	0.98
**SLC25A45**	solute carrier family 25, member 45	-2.30	0.01443	-1.09	0.75
**PCDH20**	protocadherin 20	-2.30	0.00226	-1.12	0.55
**BTBD16**	BTB (POZ) domain containing 16	-2.29	0.00114	1.1	0.58
**RHOB**	ras homolog gene family, member B	-2.29	0.00081	1.1	0.56
**PER1**	period homolog 1 (Drosophila)	-2.28	0.00018	1.03	0.82
**W57613**	---	-2.28	0.00354	1.21	0.35353

**MYCL1**	v-myc myelocytomatosis viral oncogene homolog 1, lung carcinoma derived (avian)	-2.28	0.00965	1.09	0.71
**IL24**	interleukin 24	-2.27	0.04771	1.25	0.54
**RNF150**	ring finger protein 150	-2.27	0.00026	1.25	0.11
**AW611560**	---	-2.26	0.00519	-1.13	0.56794
**AW207701**	---	-2.26	0.02039	-1.21	0.50162
**AA702409**	---	-2.25	0.02683	1.17	0.6053
**AI686793**	---	-2.25	0.00066	1.1	0.50697
**BDH1**	3-hydroxybutyrate dehydrogenase, type 1	-2.25	0.00152	1.07	0.71
**NEK4**	NIMA (never in mitosis gene a)-related kinase 4	-2.25	0.02606	-1.03	0.93
**MAP3K13**	Mitogen-activated protein kinase kinase kinase 13	-2.24	0.01821	-1.59	0.12
**NHS**	Nance-Horan syndrome (congenital cataracts and dental anomalies)	-2.23	0.00261	-1.04	0.82
**ARPC5L**	actin related protein 2/3 complex, subunit 5-like	-2.23	0.00244	-1.4	0.09
**GYG2**	glycogenin 2	-2.23	0.00261	-1.04	0.81
**ZC3H12B**	zinc finger CCCH-type containing 12B	-2.23	0.00143	-1	0.98
**AREG**	amphiregulin	-2.23	0.04495	1.11	0.84172
**BF445387**	---	-2.23	0.0027	-1.15	0.45183
**EDNRB**	endothelin receptor type B	-2.23	0.00037	-1.15	0.32
**MBD5**	methyl-CpG binding domain protein 5	-2.23	0.00097	-1.13	0.42
**RSPO3**	R-spondin 3 homolog (Xenopus laevis)	-2.23	0.04989	-1.42	0.34
**KIAA1549**	flavin containing monooxygenase 4	-2.22	0.00013	-1.3	0.04
**B4GALT1**	UDP-Gal:betaGlcNAc beta 1,4-galactosyltransferase, polypeptide 1	-2.21	0.0161	1.07	0.79
**CLCNKB**	chloride channel Kb	-2.21	0.02339	-1.27	0.41
**AL109691**	---	-2.20	0.00964	-1.11	0.64838
**STAMBPL1**	STAM binding protein-like 1	-2.20	0.0049	-1.06	0.79
**ZBTB38**	zinc finger and BTB domain containing 38	-2.19	0.04405	-1.04	0.90
**MCM10**	minichromosome maintenance complex component 10	-2.19	0.04848	1.01	0.97
**SLC35F3**	solute carrier family 35, member F3	-2.19	0.02181	1.05	0.86
**ARID5B**	AT rich interactive domain 5B (MRF1-like)	-2.18	0.00052	1.05	0.72
**DNASE1L3**	deoxyribonuclease I-like 3	-2.18	0.00419	-1.13	0.52
**AU157716**	---	-2.18	0.00082	1.03	0.85413
**AI274981**	---	-2.18	0.03251	-1	0.99629
**AU155091**	---	-2.17	0.01259	-1.02	0.94603
**FAM87A**	family with sequence similarity 87, member A	-2.17	0.00349	-1.02	0.90
**KBTBD11**	kelch repeat and BTB (POZ) domain containing 11	-2.17	0.0063	1.11	0.63
**LOC100507455**	hypothetical LOC100507455	-2.17	0.00013	1.23	0.09
**KIAA1430**	KIAA1430	-2.17	0.0074	1.09	0.68
**PC**	pyruvate carboxylase	-2.17	0.00064	1.04	0.78
**PHF17**	PHD finger protein 17	-2.17	0.00385	1.08	0.70
**FAM216A**	family with sequence similarity 216, member A	-2.16	0.00016	-1.03	0.79
**AW969265**	---	-2.16	0.01838	-1	0.98722
**LOC100505498**	uncharacterized LOC100505498	-2.16	0.00894	-1.48	0.11
**PRKCH**	protein kinase C, eta	-2.16	0.00061	1.56	0.01
**SAMD13**	sterile alpha motif domain containing 13	-2.16	0.03066	-1.44	0.25
**AW263539**	---	-2.16	0.04293	1.09	0.79281

**PXDC1**	PX domain containing 1	-2.16	0.00584	-1.16	0.47
**BM664532**	---	-2.15	0.01996	1	0.99844
**ALDH6A1**	aldehyde dehydrogenase 6 family, member A1	-2.15	0.01516	1.3	0.32
**AI434789**	---	-2.15	0.0293	1.09	0.77744
**EPHX4**	epoxide hydrolase 4	-2.15	0.00106	-1.22	0.21
**AA778565**	---	-2.15	0.01204	1.01	0.9611
**SMIM2**	small integral membrane protein 2	-2.15	0.02342	1.05	0.85
**SH3GL2**	SH3-domain GRB2-like 2	-2.15	0.00032	1.03	0.81
**AI300077**	---	-2.15	0.00412	1.42	0.09748
**BRI3BP**	BRI3 binding protein	-2.15	0.0253	1.13	0.67
**LOC100506021**	hypothetical LOC100506021	-2.15	0.01054	-1.05	0.84
**DLGAP1-AS1**	DLGAP1 antisense RNA 1	-2.14	0.04809	-1.28	0.46
**GLS2**	glutaminase 2 (liver, mitochondrial)	-2.14	0.01315	1.05	0.83
**ITPR1**	inositol 1,4,5-triphosphate receptor, type 1	-2.14	0.00162	-1.01	0.95
**ZNF141**	zinc finger protein 141	-2.13	0.00661	-1.05	0.80
**FKBP7**	FK506 binding protein 7	-2.13	0.00893	1.11	0.63
**ZNF280D**	zinc finger protein 280D	-2.13	0.00421	-1.4	0.11
**MCF2L**	MCF.2 cell line derived transforming sequence-like	-2.13	0.00022	-1.08	0.50
**WIPF1**	WAS/WASL interacting protein family, member 1	-2.13	0.00149	-1.03	0.86
**CMIP**	c-Maf inducing protein	-2.12	0.00036	1.04	0.72796
**PTP4A1**	protein tyrosine phosphatase type IVA, member 1	-2.12	0.00068	1.02	0.91
**DST**	dystonin	-2.11	0.03694	-1.38	0.30
**LOC158696**	hypothetical LOC158696	-2.11	0.00213	-1.02	0.88
**TYMS**	thymidylate synthetase	-2.10	0.02143	-1.03	0.90
**SLC7A1**	solute carrier family 7 (cationic amino acid transporter, y+ system), member 1	-2.10	0.00061	-1.19	0.21
**BCL7A**	B-cell CLL/lymphoma 7A	-2.10	0.00085	1.12	0.42
**MSX2**	msh homeobox 2	-2.10	0.01319	1.71	0.05
**BC042893**	---	-2.09	0.0284	-1.06	0.84067
**EN2**	engrailed homeobox 2	-2.09	0.01431	1.03	0.91
**AW293012**	---	-2.09	0.00093	-1.16	0.30585
**MCTP2**	multiple C2 domains, transmembrane 2	-2.09	0.00189	1.03	0.84
**FNDC3A**	Fibronectin type III domain containing 3A	-2.09	0.04589	-1.48	0.24
**ENPP2**	ectonucleotide pyrophosphatase/phosphodiesterase 2	-2.08	0.00621	1.38	0.14
**LOC100287676**	Hypothetical protein LOC100287676	-2.08	0.00125	1.03	0.86
**AA088388**	---	-2.08	0.04262	-1.32	0.37818
**AA057437**	---	-2.08	0.00985	-1.26	0.31258
**AI935541**	---	-2.07	0.00942	-1.14	0.55583
**CDK6**	cyclin-dependent kinase 6	-2.07	0.00134	1.02	0.88
**GPR98**	G protein-coupled receptor 98	-2.07	0.01515	1.16	0.53
**ORF1**	hypothetical protein, clone pT-Adv JuaX22	-2.07	0.01191	1.15	0.53
**CYFIP2**	cytoplasmic FMR1 interacting protein 2	-2.07	0.0021	1.43	0.05
**ZNF275**	zinc finger protein 275	-2.07	0.04737	1.25	0.48
**DPY19L2P2**	dpy-19-like 2 pseudogene 2 (C. elegans)	-2.07	0.01003	-1.16	0.51
**MDM2**	Mdm2 p53 binding protein homolog (mouse)	-2.07	0.00477	1.1	0.63
**AL049245**	---	-2.06	0.00369	-1.07	0.70243
**PRTG**	protogenin	-2.06	0.0091	1	1.00

**TOB1**	transducer of ERBB2, 1	-2.06	0.03573	-1.03	0.92
**SSFA2**	sperm specific antigen 2	-2.05	0.00073	-1.21	0.17
**LINC00521**	long intergenic non-protein coding RNA 521	-2.05	0.00885	-1.1	0.65
**ANKH**	ankylosis, progressive homolog (mouse)	-2.05	0.01833	-1.35	0.24
**ATPAF1**	ATP synthase mitochondrial F1 complex assembly factor 1	-2.05	0.00097	-1.33	0.07
**HCG11**	HLA complex group 11	-2.04	0.00878	1.08	0.72
**LOC100505974**	uncharacterized LOC100505974	-2.04	0.00592	-1.05	0.81
**PLLP**	plasmolipin	-2.04	0.00578	-1.07	0.73
**NCAM1**	Neural cell adhesion molecule 1	-2.04	0.00845	-1.49	0.08
**REEP1**	receptor accessory protein 1	-2.04	0.00111	-1	1.00
**NAV2**	neuron navigator 2	-2.04	0.00061	-1.41	0.03
**SMAD2**	SMAD family member 2	-2.04	0.00215	-1	0.98
**TMEM231**	transmembrane protein 231	-2.03	0.02144	-1.01	0.98
**VEPH1**	ventricular zone expressed PH domain homolog 1 (zebrafish)	-2.03	0.01998	1.05	0.84
**CAMK2B**	calcium/calmodulin-dependent protein kinase II beta	-2.03	0.00028	1	0.99
**KRT23**	keratin 23 (histone deacetylase inducible)	-2.03	0.0272	-1.29	0.35
**C11orf96**	chromosome 11 open reading frame 96	-2.03	0.01409	-1.42	0.15
**RGCC**	regulator of cell cycle	-2.03	0.03419	1.08	0.79
**ADAMTS8**	ADAM metallopeptidase with thrombospondin type 1 motif, 8	-2.02	0.00352	-1.12	0.52
**BC041941**	---	-2.02	0.04639	-1.11	0.73358
**ATOH8**	Atonal homolog 8 (Drosophila)	-2.02	0.03864	1.16	0.61
**RORA**	RAR-related orphan receptor A	-2.02	0.00052	-1.16	0.25
**LYAR**	Ly1 antibody reactive homolog (mouse)	-2.01	0.00091	-1.17	0.25
**SNAPC1**	small nuclear RNA activating complex, polypeptide 1, 43kDa	-2.01	0.00323	1.11	0.53
**TRAF3IP3**	TRAF3 interacting protein 3	-2.01	0.00273	1.28	0.16
**UQCRB**	ubiquinol-cytochrome c reductase binding protein	-2.01	0.00074	-1.19	0.21
**ACSBG1**	acyl-CoA synthetase bubblegum family member 1	-2.01	0.0213	-1.29	0.31
**ERGIC1**	endoplasmic reticulum-golgi intermediate compartment (ERGIC) 1	-2.01	0.00621	-1.04	0.84
**PRKAG2-AS1**	PRKAG2 antisense RNA 1	-2.01	0.01612	1.15	0.54
**DHX30**	DEAH (Asp-Glu-Ala-His) box polypeptide 30	-2.01	0.0169	-1.03	0.91
**NOS1**	nitric oxide synthase 1 (neuronal)	-2.01	0.01753	-1.04	0.87
**DLX6-AS1**	DLX6 antisense RNA 1	-2.00	0.02707	-1.28	0.36
**AXL**	AXL receptor tyrosine kinase	-2.00	0.00608	1.01	0.98
**LINC00313**	long intergenic non-protein coding RNA 313	-2.00	0.01814	1.13	0.60
**BU789637**	---	2.00	0.01924	-1.1	0.68819
**CDH8**	cadherin 8, type 2	2.00	0.03039	1.53	0.14
**G6PD**	glucose-6-phosphate dehydrogenase	2.00	0.01192	1.08	0.71
**ITGB3**	integrin, beta 3 (platelet glycoprotein IIIa, antigen CD61)	2.00	0.01198	-1.1	0.65
**W87626**	---	2.00	0.01801	-1.18	0.48782
**MEX3B**	mex-3 homolog B (C. elegans)	2.01	0.00195	1.26	0.16
**NREP**	neuronal regeneration related protein homolo	2.01	0.04319	-1.37	0.30
**IL17RD**	interleukin 17 receptor D	2.01	0.00123	1.1	0.52
**HOXA10**	homeobox A10	2.01	0.00161	1.37	0.06
**ITPKC**	inositol 1,4,5-trisphosphate 3-kinase C	2.01	0.01452	1.17	0.50
**TPRXL**	tetra-peptide repeat homeobox-like	2.01	0.01069	-1.24	0.32

**GNG2**	guanine nucleotide binding protein (G protein), gamma 2	2.01	0.00163	-1.07	0.64
**FAT2**	FAT tumor suppressor homolog 2 (Drosophila)	2.01	0.02442	1.14	0.62
**PDZK1IP1**	PDZK1 interacting protein 1	2.02	0.01489	1.24	0.35
**PDGFRA**	platelet-derived growth factor receptor, alpha polypeptide	2.02	0.01614	1.01	0.96
**CMTM7**	CKLF-like MARVEL transmembrane domain containing 7	2.02	0.04259	1.33	0.35
**RRAS**	related RAS viral (r-ras) oncogene homolog	2.02	6.00E-04	-1.01	0.93
**BF724621**	---	2.02	0.00546	-1.25	0.25831
**DGKH**	diacylglycerol kinase, eta	2.02	0.00133	-1.17	0.30
**GAL3ST4**	galactose-3-O-sulfotransferase 4	2.02	0.00027	1.09	0.45
**APOL6**	apolipoprotein L, 6	2.02	0.02016	1.27	0.34
**DYSF**	dysferlin, limb girdle muscular dystrophy 2B (autosomal recessive)	2.03	0.00116	-1.12	0.43
**HS3ST1**	heparan sulfate (glucosamine) 3-O-sulfotransferase 1	2.03	0.02889	-1.34	0.30
**FLJ42709**	hypothetical LOC441094	2.03	0.01393	1.05	0.82
**PADI3**	peptidyl arginine deiminase, type III	2.03	0.00887	-1.2	0.39
**PHLDB1**	pleckstrin homology-like domain, family B, member 1	2.03	0.00347	1.03	0.88
**RARRES3**	retinoic acid receptor responder (tazarotene induced) 3	2.03	0.04827	1.06	0.84
**LOC100126784**	hypothetical LOC100126784	2.03	0.00581	-1.04	0.82
**ROR1**	receptor tyrosine kinase-like orphan receptor 1	2.03	0.0211	1.7	0.06
**PRKCA**	protein kinase C, alpha	2.04	0.00066	1.02	0.88
**MIR205HG**	MIR205 host gene (non-protein coding)	2.04	0.03495	-1.15	0.63
**LOC100506621**	hypothetical LOC100506621	2.04	0.03095	1.14	0.63
**ENDOD1**	endonuclease domain containing 1	2.04	0.00857	-1.16	0.47
**AA644178**	---	2.04	0.00168	1.01	0.95939
**DFNB31**	deafness, autosomal recessive 31	2.04	0.01215	-1.05	0.83
**GPR161**	G protein-coupled receptor 161	2.04	0.03426	-1.01	0.98
**IRS1**	insulin receptor substrate 1	2.05	0.04193	1.13	0.69
**JAKMIP2**	janus kinase and microtubule interacting protein 2	2.05	0.01565	-1.09	0.72
**GRIK2**	glutamate receptor, ionotropic, kainate 2	2.05	0.00074	1.03	0.83
**AKR1C2**	aldo-keto reductase family 1, member C2 (dihydrodiol dehydrogenase 2; bile acid binding protein; 3-alpha hydroxysteroid dehydrogenase, type III)	2.05	0.04602	-1.21	0.54
**FOXL1**	forkhead box L1	2.05	0.0219	-1.15	0.59
**PLCB4**	phospholipase C, beta 4	2.05	0.02032	1.03	0.91
**CRABP2**	cellular retinoic acid binding protein 2	2.05	0.00825	-1.03	0.88
**GPR37**	G protein-coupled receptor 37 (endothelin receptor type B-like)	2.05	0.02429	-1.03	0.92
**LOC100507164**	hypothetical LOC100507164	2.06	0.01102	1.12	0.60
**CXCL1**	chemokine (C-X-C motif) ligand 1 (melanoma growth stimulating activity, alpha)	2.06	0.00038	-1.27	0.08
**NOX4**	NADPH oxidase 4	2.06	0.01202	-1.42	0.15

**CCDC80**	coiled-coil domain containing 80	2.06	0.00254	1.34	0.11
**GRIA2**	glutamate receptor, ionotropic, AMPA 2	2.06	0.00059	-1.24	0.12
**PDE5A**	phosphodiesterase 5A, cGMP-specific	2.06	0.02609	-1	1.00
**STON2**	Stonin 2	2.06	0.0371	1	0.99
**AA809487**	---	2.06	0.00884	1.16	0.49235
**ACPP**	acid phosphatase, prostate	2.06	0.00135	1.51	0.02
**GBP4**	guanylate binding protein 4	2.07	0.00809	-1.13	0.57
**IVNS1ABP**	influenza virus NS1A binding protein	2.07	0.00016	1.1	0.36
**AW953794**	---	2.07	0.01007	1.02	0.94143
**WNT9A**	wingless-type MMTV integration site family, member 9A	2.07	0.00489	-1.08	0.68
**AK021505**	---	2.08	0.002	-1.06	0.71778
**PI3**	peptidase inhibitor 3, skin-derived	2.08	0.03815	1.02	0.94
**SYNPO**	synaptopodin	2.08	0.00921	-1.05	0.83
**GREB1**	growth regulation by estrogen in breast cancer 1	2.08	0.00014	1.13	0.26
**NGEF**	neuronal guanine nucleotide exchange factor	2.08	0.00323	1.24	0.25
**RASL11B**	RAS-like, family 11, member B	2.08	0.00127	-1.07	0.63
**TP53I3**	tumor protein p53 inducible protein 3	2.09	0.00058	1.04	0.74
**ATP1B1**	ATPase, Na+/K+ transporting, beta 1 polypeptide	2.09	0.00895	1.01	0.95
**CIITA**	class II, major histocompatibility complex, transactivator	2.09	0.01009	-1	0.98
**SYNPO2**	synaptopodin 2	2.09	0.00858	-1.12	0.59
**DAPP1**	dual adaptor of phosphotyrosine and 3-phosphoinositides	2.09	0.00312	-1.04	0.80
**IRAK2**	interleukin-1 receptor-associated kinase 2	2.10	0.00105	1.45	0.03
**CAMK2D**	calcium/calmodulin-dependent protein kinase II delta	2.10	0.00099	1.11	0.46
**CYBASC3**	cytochrome b, ascorbate dependent 3	2.10	0.00236	1.03	0.88
**PDE1C**	phosphodiesterase 1C, calmodulin-dependent 70kDa	2.11	0.00514	1.11	0.59
**GBP3**	guanylate binding protein 3	2.11	0.00464	1.17	0.41
**FRMD4B**	FERM domain containing 4B	2.11	0.01174	1.3	0.27
**ALOX5AP**	arachidonate 5-lipoxygenase-activating protein	2.11	0.00802	-1.09	0.70
**REPS2**	RALBP1 associated Eps domain containing 2	2.12	0.00074	-1.08	0.59
**PITPNM3**	PITPNM family member 3	2.12	0.00107	1.01	0.93
**CLSTN2**	calsyntenin 2	2.13	0.00077	1.01	0.96
**GADD45B**	Growth arrest and DNA-damage-inducible, beta	2.13	0.0233	1.35	0.29
**KIAA0226L**	KIAA0226-like	2.13	0.01072	1.04	0.88
**LINC00626**	long intergenic non-protein coding RNA 626	2.13	0.00133	-1.3	0.12
**NGB**	neuroglobin	2.13	0.00131	1.02	0.88
**TMCC2**	transmembrane and coiled-coil domain family 2	2.13	0.0035	1.08	0.69
**RDH16**	retinol dehydrogenase 16 (all-trans)	2.14	0.00598	-1.16	0.48
**BEAN1**	brain expressed, associated with NEDD4, 1	2.14	0.00478	1.12	0.56
**CYP1B1**	cytochrome P450, family 1, subfamily B, polypeptide 1	2.14	0.00679	1.07	0.76
**AW292830**	---	2.14	0.00133	1.02	0.91966
**DDX60L**	DEAD (Asp-Glu-Ala-Asp) box polypeptide 60-like	2.14	0.02071	1.09	0.75

**FBLIM1**	filamin binding LIM protein 1	2.14	0.01025	-1.01	0.95
**WFDC2**	WAP four-disulfide core domain 2	2.14	0.00652	1.15	0.50
**KIAA1841**	KIAA1841	2.15	0.00068	-1.06	0.66
**IGSF9**	immunoglobulin superfamily, member 9	2.15	0.00934	1.04	0.88
**AU151465**	---	2.15	0.00373	-1.22	0.31827
**ETS1**	v-ets erythroblastosis virus E26 oncogene homolog 1 (avian)	2.17	0.00029	-1.02	0.86
**AW044663**	---	2.17	0.01897	1.12	0.67908
**GFPT2**	glutamine-fructose-6-phosphate transaminase 2	2.17	0.02835	-1.08	0.80
**SERTAD4**	SERTA domain containing 4	2.17	0.00122	-1.1	0.54
**MMP2**	matrix metallopeptidase 2 (gelatinase A, 72kDa gelatinase, 72kDa type IV collagenase)	2.17	0.01207	-1.06	0.81
**WNT10A**	Wingless-type MMTV integration site family, member 10A	2.17	0.00265	-1.2	0.33
**LOC728978**	uncharacterized LOC728978	2.18	0.00056	1	0.99
**MARCKS**	myristoylated alanine-rich protein kinase C substrate	2.18	0.00228	1.13	0.50
**HRH1**	histamine receptor H1	2.18	0.00533	1.2	0.39
**TFEB**	transcription factor EB	2.18	0.00165	-1.16	0.39
**ATP6V0D2**	ATPase, H+ transporting, lysosomal 38kDa, V0 subunit d2	2.18	0.02069	-1.39	0.25
**SGK223**	homolog of rat pragma of Rnd2	2.19	0.00839	1.17	0.48
**ATP2B4**	ATPase, Ca++ transporting, plasma membrane 4	2.19	0.01555	1.12	0.67
**KIF3C**	kinesin family member 3C	2.19	0.00127	1.13	0.46
**PDGFB**	platelet-derived growth factor beta polypeptide (simian sarcoma viral (v-sis) oncogene homolog)	2.19	0.00056	1.04	0.76
**LOC100292680**	hypothetical LOC100292680	2.19	0.00376	-1.16	0.45
**AI935710**	---	2.20	0.00784	-1.31	0.24662
**CDK15**	cyclin-dependent kinase 15	2.20	0.01575	-1.24	0.42
**CYP4F3**	cytochrome P450, family 4, subfamily F, polypeptide 3	2.21	0.0361	1.12	0.73
**LYST**	lysosomal trafficking regulator	2.21	0.00093	1.19	0.26
**NLRC5**	NLR family, CARD domain containing 5	2.21	0.00966	1.13	0.60
**FN1**	fibronectin 1	2.23	0.00031	-1.29	0.08
**LOX**	lysyl oxidase	2.23	0.00388	1.02	0.91
**PRR5L**	proline rich 5 like	2.23	0.00145	-1	0.99
**RPRM**	reprimo, TP53 dependent G2 arrest mediator candidate	2.23	0.00472	-1.13	0.55
**PTGER4**	prostaglandin E receptor 4 (subtype EP4)	2.23	0.04292	-1.07	0.85
**GLIPR1**	GLI pathogenesis-related 1	2.23	0.03537	1.08	0.80
**LOC643792**	contactin associated protein-like 3 pseudogene	2.23	0.00128	1.17	0.36
**RUNDC3B**	RUN domain containing 3B	2.23	0.03495	1.04	0.91
**PLEKHG1**	pleckstrin homology domain containing, family G (with RhoGef domain) member 1	2.24	0.00093	1.08	0.62
**NAV3**	neuron navigator 3	2.24	0.01017	-1.03	0.89
**CA430162**	---	2.25	0.00075	1.15	0.36079
**HOMER2**	homer homolog 2 (Drosophila)	2.25	0.00028	1.04	0.74
**COL6A3**	collagen, type VI, alpha 3	2.25	0.00034	-1.11	0.45
**R20640**	---	2.25	0.00275	-1.42	0.09416

**GBP6**	guanylate binding protein family, member 6	2.25	0.00273	1.15	0.47
**KALRN**	kalirin, RhoGEF kinase	2.26	0.01337	1.01	0.96
**C8orf48**	chromosome 8 open reading frame 48	2.27	0.01136	-1	1.00
**SERPINE2**	serpin peptidase inhibitor, clade E (nexin, plasminogen activator inhibitor type 1), member 2	2.27	0.02918	1.14	0.68
**CDKN1C**	cyclin-dependent kinase inhibitor 1C (p57, Kip2)	2.27	0.00873	1.01	0.98
**SERPINA1**	serpin peptidase inhibitor, clade A (alpha-1 antiproteinase, antitrypsin), member 1	2.27	0.00614	-1	1.00
**AI936724**	---	2.27	0.00302	-1.29	0.21124
**FRMD6**	FERM domain containing 6	2.28	0.00078	1.19	0.28
**ST8SIA1**	ST8 alpha-N-acetyl-neuraminide alpha-2,8-sialyltransferase 1	2.28	0.00262	-1.03	0.90
**FMO4**	flavin containing monooxygenase 4	2.28	0.00582	1.7	0.04
**HMGA2**	high mobility group AT-hook 2	2.28	0.0189	-1.17	0.58
**SHB**	Src homology 2 domain containing adaptor protein B	2.28	0.02295	-1	1.00
**ROS1**	c-ros oncogene 1 , receptor tyrosine kinase	2.28	0.00038	-1	0.99
**CYP1A1**	cytochrome P450, family 1, subfamily A, polypeptide 1	2.29	0.0261	1.1	0.77
**MLPH**	melanophilin	2.29	0.0174	1.16	0.59
**SAA4**	serum amyloid A4, constitutive	2.30	0.00614	1.17	0.49
**ALPK2**	alpha-kinase 2	2.30	0.00231	-1.17	0.41
**RASD2**	RASD family, member 2	2.30	0.00523	-1.18	0.47
**CNTNAP3**	contactin associated protein-like 3	2.30	0.00013	1.26	0.08
**AW189097**	---	2.30	0.01593	-1.07	0.80029
**RUNX1**	runt-related transcription factor 1	2.30	0.00957	-1.47	0.15
**TLR2**	toll-like receptor 2	2.30	0.02504	-1.04	0.90
**MAMDC2**	MAM domain containing 2	2.31	0.01338	1.15	0.61
**MAF**	v-maf musculoaponeurotic fibrosarcoma oncogene homolog (avian)	2.31	0.01038	1.02	0.93
**N73742**	---	2.31	0.00379	1.13	0.54978
**LRRC7**	leucine rich repeat containing 7	2.31	0.02334	1.26	0.46
**LOXL2**	lysyl oxidase-like 2	2.32	0.0121	1.19	0.52
**ADM**	adrenomedullin	2.32	0.00796	1.19	0.48
**ELF3**	E74-like factor 3 (ets domain transcription factor, epithelial-specific)	2.33	0.04535	-1.03	0.94
**EPB41L4B**	erythrocyte membrane protein band 4.1 like 4B	2.33	0.00082	-1	0.98
**DUSP10**	dual specificity phosphatase 10	2.33	0.00032	-1.22	0.17
**PLA2R1**	phospholipase A2 receptor 1, 180kDa	2.33	0.00637	1.03	0.88508
**RHBDL2**	rhomboid, veinlet-like 2 (Drosophila)	2.33	0.00279	1	0.99
**AI223854**	---	2.34	0.00425	1.13	0.57936
**BC040310**	---	2.34	0.00044	1.03	0.85012
**MX1**	myxovirus (influenza virus) resistance 1, interferon-inducible protein p78 (mouse)	2.34	0.00665	1.19	0.47
**PLAC2**	placenta-specific 2 (non-protein coding)	2.34	0.00424	1.09	0.70
**ANKRD55**	Ankyrin repeat domain 55	2.34	0.03682	-1.06	0.87
**BF110268**	---	2.35	0.02002	1.01	0.9638
**UAP1L1**	UDP-N-acteylglucosamine pyrophosphorylase 1-like 1	2.35	0.00283	-1.15	0.49
**CAMK2A**	calcium/calmodulin-dependent protein kinase II alpha	2.35	0.00052	1	0.98

**HERC5**	hect domain and RLD 5	2.36	0.00028	1.03	0.83
**AW292765**	---	2.37	0.00376	1.11	0.61664
**MTMR11**	myotubularin related protein 11	2.37	0.00138	1.08	0.67
**IFITM10**	interferon induced transmembrane protein 10	2.38	0.04561	1.06	0.88
**OPTN**	optineurin	2.38	4.00E-04	1.08	0.60
**CAV1**	caveolin 1, caveolae protein, 22kDa	2.38	0.03554	-1.02	0.96
**APOBEC3B**	apolipoprotein B mRNA editing enzyme, catalytic polypeptide-like 3B	2.39	0.00196	1.21	0.34
**LAMC2**	laminin, gamma 2	2.39	0.01315	-1.03	0.90
**UPP1**	uridine phosphorylase 1	2.39	0.02813	-1.04	0.90
**FTH1**	ferritin, heavy polypeptide 1	2.39	0.00426	1.06	0.79
**BC041915 ***	---	2.41	0.00502	-2.89	0.0018
**SLIT3**	slit homolog 3 (Drosophila)	2.41	0.00012	1.02	0.8795
**COL5A1**	collagen, type V, alpha 1	2.41	0.00067	-1.09	0.59
**DDX60**	DEAD (Asp-Glu-Ala-Asp) box polypeptide 60	2.41	0.00374	1.16	0.50
**NMNAT2**	nicotinamide nucleotide adenylyltransferase 2	2.41	0.01638	1.17	0.60
**ZEB1**	zinc finger E-box binding homeobox 1	2.41	0.0028	1.17	0.45
**TRIM47**	tripartite motif-containing 47	2.42	0.00066	-1.03	0.86
**AA702930**	---	2.43	0.02414	-1.18	0.61656
**CA442342**	---	2.43	0.00012	1.02	0.84539
**LFNG**	LFNG O-fucosylpeptide 3-beta-N-acetylglucosaminyltransferase	2.44	0.00201	-1.08	0.70
**SLC2A12**	solute carrier family 2 (facilitated glucose transporter), member 12	2.44	0.00246	-1.07	0.73
**IGFBP5**	insulin-like growth factor binding protein 5	2.45	0.00098	1.69	0.02
**AF339789**	---	2.46	0.0366	1.14	0.71273
**MMP10**	matrix metallopeptidase 10 (stromelysin 2)	2.46	0.01733	-1.08	0.80
**TNF**	tumor necrosis factor	2.46	0.00053	-1.01	0.96
**KCND3**	potassium voltage-gated channel, Shal-related subfamily, member 3	2.46	0.00231	1.13	0.56
**C1orf116**	chromosome 1 open reading frame 116	2.47	0.00197	1.01	0.97
**TGFB1I1**	transforming growth factor beta 1 induced transcript 1	2.47	0.01473	1.02	0.96
**TFCP2L1**	transcription factor CP2-like 1	2.47	0.00346	-1.1	0.66
**KLF7**	Kruppel-like factor 7 (ubiquitous)	2.47	0.00165	1.07	0.74
**LCP1**	lymphocyte cytosolic protein 1 (L-plastin)	2.47	0.0248	-1.42	0.31
**VGLL3**	vestigial like 3 (Drosophila)	2.48	0.04286	1.61	0.24
**NUAK2**	NUAK family, SNF1-like kinase, 2	2.49	0.00441	1.3	0.28
**CTHRC1**	collagen triple helix repeat containing 1	2.49	0.00273	1.02	0.94
**PAX5**	paired box 5	2.49	6.00E-05	-1.74	0.00
**RUNX1-IT1**	RUNX1 intronic transcript 1 (non-protein codir	2.50	0.02351	1.16	0.67
**BC020897**	---	2.50	0.01663	-1.02	0.95992
**MAN1A1**	mannosidase, alpha, class 1A, member 1	2.52	0.00193	1.55	0.06
**PPAP2B**	phosphatidic acid phosphatase type 2B	2.52	0.01711	1.24	0.49
**SCN3B**	sodium channel, voltage-gated, type III, beta	2.52	0.00298	1.1	0.66
**BCL11B**	B-cell CLL/lymphoma 11B (zinc finger protein)	2.52	0.00412	1.02	0.94
**GLIS1**	GLIS family zinc finger 1	2.52	0.001	1.19	0.34
**SLC6A20**	solute carrier family 6 (proline IMINO transporter), member 20	2.52	0.00993	-1.13	0.67
**CNPY3**	canopy 3 homolog (zebrafish)	2.53	0.00913	-1.13	0.65
**EMP1**	epithelial membrane protein 1	2.53	0.00554	-1.01	0.96
**NKAIN4**	Na+/K+ transporting ATPase interacting 4	2.54	0.00059	1.06	0.71

**IL32**	interleukin 32	2.54	0.00512	-1.04	0.88
**BM873997**	---	2.54	0.00723	-1.08	0.76795
**PROC**	protein C (inactivator of coagulation factors Va and VIIIa)	2.55	0.00161	-1.11	0.59
**SEMA3E**	sema domain, immunoglobulin domain (Ig), short basic domain, secreted, (semaphorin) 3E	2.55	0.03018	-1.06	0.87
**SCN2A**	sodium channel, voltage-gated, type II, alpha subunit	2.56	0.04753	-1.55	0.30
**CYP27C1**	cytochrome P450, family 27, subfamily C, polypeptide 1	2.58	0.02897	1.02	0.95
**ARHGEF28**	Rho guanine nucleotide exchange factor (GEF)	2.58	4.00E-05	-1.05	0.66
**BF508849**	---	2.58	0.00127	-1.02	0.93729
**CAPN6**	calpain 6	2.59	0.00064	-1.46	0.06
**PLCE1**	phospholipase C, epsilon 1	2.60	0.00048	1.05	0.75
**BM992214**	---	2.60	0.00428	1.1	0.70284
**GULP1**	GULP, engulfment adaptor PTB domain containing 1	2.60	0.00093	1.16	0.43
**TMEM200A**	transmembrane protein 200A	2.60	0.0012	1.07	0.72
**C10orf10**	chromosome 10 open reading frame 10	2.60	0.02025	1.42	0.31
**IL16**	interleukin 16 (lymphocyte chemoattractant factor)	2.60	0.00629	1.06	0.83
**W23691**	---	2.60	0.00139	-1.07	0.73078
**ARL4C**	ADP-ribosylation factor-like 4C	2.61	5.00E-05	1.08	0.49
**TEAD2**	TEA domain family member 2	2.61	0.01421	1.05	0.87
**CD44**	CD44 molecule (Indian blood group)	2.62	0.00099	1.07	0.71
**LOXL4**	lysyl oxidase-like 4	2.63	0.00381	1.04	0.86
**SYT1**	synaptotagmin I	2.63	0.00153	-1.06	0.77
**CSF1R**	colony stimulating factor 1 receptor	2.63	0.00231	-1.65	0.05
**HS3ST2**	heparan sulfate (glucosamine) 3-O-sulfotransferase 2	2.64	0.00102	1.16	0.45
**PSG1**	pregnancy specific beta-1-glycoprotein 1	2.64	0.03158	-1.05	0.90
**R71596**	---	2.64	0.00974	-1.06	0.83822
**MALL**	mal, T-cell differentiation protein-like	2.65	0.00358	1.26	0.34
**DSG3**	desmoglein 3	2.66	7.00E-05	1.04	0.74
**STRA6**	stimulated by retinoic acid gene 6 homolog (mouse)	2.66	0.00108	1.08	0.68
**C20orf160**	chromosome 20 open reading frame 160	2.66	0.0102	-1.02	0.96
**LGALS3**	lectin, galactoside-binding, soluble, 3	2.66	0.00032	1.02	0.90
**BC025343**	---	2.68	0.03919	-1.15	0.73487
**CTSS**	cathepsin S	2.70	0.00603	1.23	0.45
**C3orf52**	chromosome 3 open reading frame 52	2.72	0.00756	-1.06	0.8312
**NID1**	nidogen 1	2.72	0.00362	-1.15	0.57
**CORO2B**	coronin, actin binding protein, 2B	2.72	0.0018	-1.11	0.63
**AI245300**	---	2.73	0.00861	-1.15	0.64071
**BF110534**	---	2.73	1.00E-04	-1.33	0.06382
**LURAP1L**	leucine rich adaptor protein 1-like	2.73	0.00655	1.03	0.90
**MARK1**	MAP/microtubule affinity-regulating kinase 1	2.74	0.00072	1.02	0.91
**KIAA1211L**	KIAA1211-like	2.75	0.00177	1.15	0.52
**L1CAM**	L1 cell adhesion molecule	2.75	0.01583	-1.06	0.87
**PLK2**	polo-like kinase 2	2.75	0.00141	1.27	0.28
**DSC2**	desmocollin 2	2.76	0.00066	1.23	0.29
**IVL**	involucrin	2.77	7.00E-05	-1.19	0.21
**SEC14L2**	SEC14-like 2 (S. cerevisiae)	2.78	0.00167	1.14	0.55

**LRRN1**	leucine rich repeat neuronal 1	2.79	8.00E-05	-1.62	0.01
**SFN**	stratifin	2.79	0.00106	-1.03	0.88
**MIAT**	myocardial infarction associated transcript (non-protein coding)	2.81	0.00368	-1.06	0.81
**AI733336**	---	2.82	0.00076	-1.08	0.69344
**ZNF365**	zinc finger protein 365	2.83	0.00018	-1.02	0.88
**DAPL1**	death associated protein-like 1	2.83	0.00749	1.45	0.23
**SCN3A**	sodium channel, voltage-gated, type III, alpha subunit	2.84	0.02159	-1.86	0.13
**SAMD9L**	sterile alpha motif domain containing 9-like	2.84	0.01154	1.21	0.56
**FAM49A**	family with sequence similarity 49, member A	2.84	9.00E-05	1.33	0.07
**KRT6B**	keratin 6B	2.84	0.00038	1.07	0.70
**BTBD19**	BTB (POZ) domain containing 19	2.85	0.04713	1.16	0.74
**BC028670**	---	2.85	0.00075	1.15	0.4797
**HDAC9**	histone deacetylase 9	2.86	0.00363	1.28	0.35
**KRT14**	keratin 14	2.86	0.00215	-1.11	0.65
**FILIP1L**	filamin A interacting protein 1-like	2.86	0.00308	1.08	0.77
**IFIT2**	interferon-induced protein with tetratricopeptide repeats 2	2.86	0.00031	1.29	0.16
**AF009316**	---	2.87	0.00383	-1.31	0.32074
**TSHZ3**	teashirt zinc finger homeobox 3	2.88	0.00219	1.1	0.68
**DUSP5P**	dual specificity phosphatase 5 pseudogene	2.89	5.00E-04	-1.07	0.71
**GPER**	G protein-coupled estrogen receptor 1	2.89	8.00E-05	1.08	0.56
**RSAD2**	radical S-adenosyl methionine domain containing 2	2.90	0.00155	-1.44	0.13
**UNC5CL**	unc-5 homolog C (C. elegans)-like	2.90	0.00036	-1.01	0.95
**AACSP1**	acetoacetyl-CoA synthetase pseudogene 1	2.91	0.00358	1.01	0.97
**GBP2**	guanylate binding protein 2, interferon-inducible	2.91	0.01575	1.24	0.55
**PLXDC1**	plexin domain containing 1	2.91	0.00125	1.1	0.67
**ALDH1A3**	aldehyde dehydrogenase 1 family, member A3	2.92	0.00026	1.1	0.56
**HPGD**	hydroxyprostaglandin dehydrogenase 15-(NAD)	2.92	0.0414	1.29	0.57
**TUBA1A**	tubulin, alpha 1a	2.94	0.00044	1.01	0.96
**THBD**	thrombomodulin	2.96	0.00033	1.02	0.92
**KCNJ15**	potassium inwardly-rectifying channel, subfamily J, member 15	2.97	0.00436	-1.35	0.30
**STK32A**	serine/threonine kinase 32A	2.97	0.00116	1.06	0.80
**CLEC7A**	C-type lectin domain family 7, member A	2.99	0.01612	-1.03	0.93
**AK026743**	---	2.99	0.01533	-1.16	0.68377
**AKR1C1**	aldo-keto reductase family 1, member C1 (dihydrodiol dehydrogenase 1; 20-alpha (3-alpha)-hydroxysteroid dehydrogenase)	3.00	0.00137	-1.03	0.91
**MAP7D2**	MAP7 domain containing 2	3.00	3.00E-05	1.11	0.40
**RASSF6**	Ras association (RalGDS/AF-6) domain family member 6	3.01	0.00019	1.14	0.45
**CXCL10**	chemokine (C-X-C motif) ligand 10	3.01	0.00484	-1.04	0.88
**BLNK**	B-cell linker	3.03	0.0093	1.02	0.96
**APCDD1L-AS1**	APCDD1L antisense RNA 1	3.04	0.00017	-1.37	0.08
**KRT16**	keratin 16	3.04	0.00032	-1.18	0.37
**RTTN**	rotatin	3.06	0.00032	-1.05	0.79

**ST6GAL1**	ST6 beta-galactosamide alpha-2,6-sialyltranferase 1	3.06	2.00E-05	1.25	0.10
**AKR1B10**	aldo-keto reductase family 1, member B10 (aldose reductase)	3.06	0.00128	1.28	0.30
**UGT1A1**	UDP glucuronosyltransferase 1 family, polypeptide A1	3.06	0.00336	1.61	0.11
**SLAMF7**	SLAM family member 7	3.08	3.00E-04	1.08	0.65
**MYLK**	myosin light chain kinase	3.09	0.00691	1.06	0.85
**TAGLN**	transgelin	3.10	0.04019	1.26	0.62
**MERTK**	c-mer proto-oncogene tyrosine kinase	3.13	0.0346	1.13	0.79
**AF339813**	---	3.13	0.04392	-1.88	0.21725
**TNFAIP3**	tumor necrosis factor, alpha-induced protein 3	3.13	0.00128	1.34	0.23
**NANOS1**	nanos homolog 1 (Drosophila)	3.14	0.00516	1.24	0.48061
**RIMS2**	regulating synaptic membrane exocytosis 2	3.15	0.00195	-1.37	0.23
**SERPINB13**	serpin peptidase inhibitor, clade B (ovalbumin), member 13	3.17	0.00085	-1	0.99
**LPAR5**	lysophosphatidic acid receptor 5	3.18	6.00E-05	1.05	0.71
**PAMR1**	peptidase domain containing associated with muscle regeneration 1	3.20	0.00053	-1.33	0.19
**MAFB**	v-maf musculoaponeurotic fibrosarcoma oncogene homolog B (avian)	3.20	0.01557	1.04	0.91
**PDZK1**	PDZ domain containing 1	3.21	0.0207	-1	0.99
**ADAM12**	ADAM metallopeptidase domain 12	3.22	0.00035	1.02	0.92
**TNFSF15**	tumor necrosis factor (ligand) superfamily, me	3.22	0.00739	1.64	0.15862
**LRRC4C**	leucine rich repeat containing 4C	3.22	0.00111	-1.81	0.03
**OAS1**	2',5'-oligoadenylate synthetase 1, 40/46kDa	3.25	0.00216	1.24	0.42
**BF509371**	---	3.26	0.00287	1.29	0.36837
**TNFAIP2**	tumor necrosis factor, alpha-induced protein 2	3.26	0.00256	1.05	0.87
**CTSH**	cathepsin H	3.27	9.00E-05	-1.03	0.83
**PIM1**	pim-1 oncogene	3.29	1.00E-05	1.05	0.67
**ATP13A2**	ATPase type 13A2	3.29	0.00037	-1.02	0.92
**CSF1**	colony stimulating factor 1 (macrophage)	3.30	0.00859	1.39	0.36
**ITGB6**	integrin, beta 6	3.34	0.00944	-1.07	0.84
**TNFSF10**	tumor necrosis factor (ligand) superfamily, member 10	3.34	0.00567	1.14	0.69
**DLX2**	distal-less homeobox 2	3.34	0.00143	1.27	0.35
**R83322**	---	3.35	0.00277	-1.48	0.19026
**APCDD1L**	adenomatosis polyposis coli down-regulated 1-like	3.37	7.00E-05	-1.02	0.89
**GSTA4**	glutathione S-transferase alpha 4	3.38	0.00147	1.1	0.69
**MMP9**	matrix metallopeptidase 9 (gelatinase B, 92kDa gelatinase, 92kDa type IV collagenase)	3.42	0.00353	1.23	0.50
**PSG4**	pregnancy specific beta-1-glycoprotein 4	3.44	0.01617	-1.03	0.94
**IFIT3**	interferon-induced protein with tetratricopeptide repeats 3	3.45	2.00E-05	1.23	0.13
**GPR115**	G protein-coupled receptor 115	3.48	1.00E-04	1.03	0.87
**AU155234**	---	3.50	0.00096	-1.32	0.2709
**IFIT1**	interferon-induced protein with tetratricopeptide repeats 1	3.50	0.00049	1.16	0.50
**PSG7**	pregnancy specific beta-1-glycoprotein 7 (gene/pseudogene)	3.51	0.02646	-1.41	0.47
**PLAU**	plasminogen activator, urokinase	3.55	0.00081	-1.01	0.95

**AF318340**	---	3.57	0.00437	-1.35	0.3674
**AHNAK2**	AHNAK nucleoprotein 2	3.58	0.00281	1.04	0.89
**NRP1**	neuropilin 1	3.61	0.01165	-1.16	0.71
**SLC47A2**	solute carrier family 47, member 2	3.61	0.00311	1.21	0.54
**BC033184**	---	3.61	0.01033	-1.59	0.25325
**IGFL1**	IGF-like family member 1	3.62	0.00797	1.39	0.38
**VTCN1**	V-set domain containing T cell activation inhibitor 1	3.62	0.00121	1.39	0.23
**OASL**	2'-5'-oligoadenylate synthetase-like	3.66	0.00153	-1	0.99
**HES2**	hairy and enhancer of split 2 (Drosophila)	3.67	0.01278	-1.18	0.69
**BHLHE41**	basic helix-loop-helix family, member e41	3.70	0.00104	-1.09	0.75
**LOC100506374**	uncharacterized LOC100506374	3.70	0.00108	1.2	0.48
**VSNL1**	visinin-like 1	3.72	0.00093	-1.08	0.76
**PRLR**	prolactin receptor	3.74	0.0016	1.08	0.79
**LY96**	lymphocyte antigen 96	3.83	0.00309	-1.01	0.97
**SH3RF2**	SH3 domain containing ring finger 2	3.83	2.00E-05	1.05	0.76
**IFI44L**	interferon-induced protein 44-like	3.83	0.0069	-1.17	0.67
**MFAP2**	microfibrillar-associated protein 2	3.87	0.00492	1.12	0.74
**TGM5**	transglutaminase 5	3.87	0.00299	1.1	0.77
**SEMA5A**	sema domain, seven thrombospondin repeats (type 1 and type 1-like), transmembrane domain (TM) and short cytoplasmic domain, (semaphorin) 5A	3.87	0.00374	-1.37	0.36
**RASGRF1**	Ras protein-specific guanine nucleotide-releasing factor 1	3.88	4.00E-05	-1.01	0.97
**MX2**	myxovirus (influenza virus) resistance 2 (mouse)	3.91	0	1.02	0.82
**KLK6**	kallikrein-related peptidase 6	3.98	2.00E-05	-1.05	0.74
**SNCAIP**	synuclein, alpha interacting protein	4.03	0.00597	-1.15	0.71
**SLC22A3**	solute carrier family 22 (extraneuronal monoamine transporter), member 3	4.12	2.00E-05	1.07	0.63
**CXCL14**	chemokine (C-X-C motif) ligand 14	4.13	0.0139	1.09	0.85
**ARL14**	ADP-ribosylation factor-like 14	4.13	0.00047	-1.25	0.37
**ERC1**	ELKS/RAB6-interacting/CAST family member 1	4.15	9.00E-05	1.21	0.33
**MAP2**	microtubule-associated protein 2	4.21	1.00E-04	1.29	0.21
**ABLIM3**	actin binding LIM protein family, member 3	4.23	0.00021	1.22	0.38
**MMP28**	matrix metallopeptidase 28	4.23	0.00033	1.03	0.91
**HMOX1**	heme oxygenase (decycling) 1	4.24	0.00012	1.05	0.82
**IGF2BP3**	insulin-like growth factor 2 mRNA binding protein 3	4.25	0.00021	-1.02	0.93
**KLK10**	kallikrein-related peptidase 10	4.27	1.00E-04	1.2	0.37
**EPHA4**	EPH receptor A4	4.30	0.00042	-1.02	0.94
**WNT5B**	wingless-type MMTV integration site family, member 5B	4.31	2.00E-05	1.27	0.16
**ADTRP**	androgen-dependent TFPI-regulating protein	4.37	5.00E-05	1.23	0.28
**TCHH**	trichohyalin	4.40	1.00E-04	-1.05	0.79
**LYPD1**	LY6/PLAUR domain containing 1	4.42	2.00E-04	1.05	0.82
**P2RY6**	pyrimidinergic receptor P2Y, G-protein coupled, 6	4.42	0	-1.05	0.71
**FABP4**	fatty acid binding protein 4, adipocyte	4.45	0.01837	1.49	0.45
**SLITRK6**	SLIT and NTRK-like family, member 6	4.46	1.00E-05	-1.32	0.08
**SERPINB7**	serpin peptidase inhibitor, clade B (ovalbumin), member 7	4.54	0.01435	1.06	0.90

**C18orf1**	chromosome 18 open reading frame 1	4.58	0.0013	1.08	0.81
**AI694413**	---	4.61	0.02425	-1.1	0.86638
**GPR39**	G protein-coupled receptor 39	4.64	0.00029	1.34	0.26
**CLCA2**	chloride channel accessory 2	4.68	0.00369	1.08	0.83
**ABCA1**	ATP-binding cassette, sub-family A (ABC1), member 1	4.70	2.00E-05	1.06	0.72
**MTSS1**	metastasis suppressor 1	4.83	0.00047	1.04	0.90
**AI819863**	---	4.87	0.00019	1.1	0.67672
**KRT75**	keratin 75	4.93	0.00057	1.15	0.63
**NEURL3**	neuralized homolog 3 (Drosophila) pseudogene	5.03	0	-1.1	0.48
**CHI3L1**	chitinase 3-like 1 (cartilage glycoprotein-39)	5.08	0.00545	1	1.00
**C4orf26**	chromosome 4 open reading frame 26	5.16	0	1.16	0.26
**PHEX**	phosphate regulating endopeptidase homolog, X-linked	5.21	0.0014	-1.01	0.98
**KRT6A**	keratin 6A	5.22	4.00E-05	1.24	0.27
**KLK7**	kallikrein-related peptidase 7	5.24	0.00023	1.37	0.23
**CXCL12**	chemokine (C-X-C motif) ligand 12	5.33	0.01159	-1.1	0.86
**BG010493**	---	5.34	0.002	-1.08	0.84059
**SPRR1B**	small proline-rich protein 1B	5.39	6.00E-05	1.01	0.98
**SPOCD1**	SPOC domain containing 1	5.54	0.00084	-1.18	0.61
**C6orf141**	chromosome 6 open reading frame 141	5.57	0	1.01	0.96
**KRT6C**	keratin 6C	5.58	3.00E-05	-1.09	0.65
**GBP1**	guanylate binding protein 1, interferon-inducible, 67kDa	5.65	6.00E-05	-1.02	0.93
**DACT1**	dapper, antagonist of beta-catenin, homolog 1	5.65	0.00065	1.89	0.07
**FBXO32**	F-box protein 32	5.68	0.00022	1.39	0.23
**TNC**	tenascin C	5.68	9.00E-05	-1.04	0.85
**AL389956**	---	5.70	0.00055	1.03	0.92538
**BF244402**	---	5.75	1.00E-05	1.74	0.00616
**SERPINA3**	serpin peptidase inhibitor, clade A (alpha-1 antiproteinase, antitrypsin), member 3	5.78	0.00155	-1.45	0.33
**MMP13**	matrix metallopeptidase 13 (collagenase 3)	5.84	0.00162	-1.14	0.72
**AI333596**	---	5.88	0.00143	1.02	0.96015
**CST6**	cystatin E/M	5.90	1.00E-05	1.05	0.79
**MMP12**	matrix metallopeptidase 12 (macrophage elastase)	6.06	0.00429	-1.12	0.80
**TRIM22**	tripartite motif-containing 22	6.10	8.00E-04	1.01	0.97
**TNFSF11**	tumor necrosis factor (ligand) superfamily, member 11	6.38	0.00206	-1.11	0.80
**SCN9A**	sodium channel, voltage-gated, type IX, alpha subunit	6.49	0	-1.03	0.86
**KRT15**	keratin 15	6.65	0.00113	1.48	0.31
**IFI44**	Interferon-induced protein 44	7.11	0.00021	1.02	0.94
**VAV3**	vav 3 guanine nucleotide exchange factor	7.17	0.00034	1.22	0.53
**FLG-AS1**	FLG antisense RNA 1	7.24	2.00E-05	-1.1	0.65
**KLK5**	kallikrein-related peptidase 5	7.66	0	-1.04	0.81
**GABBR1**	gamma-aminobutyric acid (GABA) B receptor, 1	7.82	0.0038	1.14	0.80
**FYB**	FYN binding protein	8.14	0.00032	1.22	0.56
**TSPAN2**	tetraspanin 2	8.66	3.00E-05	1.18	0.50
**GRIN2A**	glutamate receptor, ionotropic, N-methyl D-aspartate 2A	9.73	6.00E-05	-1.01	0.97

**MMP1**	matrix metallopeptidase 1 (interstitial collagenase)	11.48	7.00E-05	-1.5	0.22
**SERPINE1**	serpin peptidase inhibitor, clade E (nexin, plasminogen activator inhibitor type 1), member 1	15.77	0	-1.05	0.79
**GPNMB**	glycoprotein (transmembrane) nmb	16.00	2.00E-05	1.17	0.58
**KANK4**	KN motif and ankyrin repeat domains 4	16.06	0	1.02	0.94
**MMP3**	matrix metallopeptidase 3 (stromelysin 1, progelatinase)	16.38	0.00023	-1.08	0.86
**CCL2**	chemokine (C-C motif) ligand 2	42.02	0	1.04	0.88

Table S2. A list of differentially regulated genes affected by CREB depletion in human fusion-positive MEC, but not fusion-negative cells. The The differentially expressed genes after CREB was depleted were identified in both fusion-positive MEC H3118 cells and fusion-negative HSY cells. Those CREB-regulated genes in H3118 cells showing the same regulated direction in fusion-negative HSY were then filtered out. The "positive" and "negative" signs denote up-regulated or down-regulated genes in KD compared to control groups, respectively. The asterisk indicates the common gene in different regulatory direction between HSY and H3118 cells.

Gene Symbol	Gene Title	Fold change CREB KD/Control (H3118)	p-value (H3118)	Fold change CREB KD/Control (HSY)	p-value (HSY)
**LOC100288985**	Hypothetical protein LOC100288985	-41.04	8.00E-05	1.34	0.63291
**STC1**	stanniocalcin 1	-26.98	2.00E-05	1.07	0.88257
**SMOX**	spermine oxidase	-21.45	0	-1.57	0.08883
**BCAT1**	branched chain amino-acid transaminase 1, cytosolic	-21.11	1.00E-05	1.12	0.7643
**DUSP6**	dual specificity phosphatase 6	-20.44	0	-1.02	0.9561
**AREG**	amphiregulin	-18.34	0	-1.04	0.91531
**NGFR**	nerve growth factor receptor	-16.22	0	-1.11	0.56875
**GPC6**	glypican 6	-14.21	0	1.13	0.51143
**ATP6V0A4**	ATPase, H+ transporting, lysosomal V0 subunit a4	-13.97	0	1.02	0.94703
**PLAT**	plasminogen activator, tissue	-13.61	1.00E-05	-1.35	0.39202
**AI093492**	---	-12.24	0.00023	-2.44	0.07764
**GDF15**	growth differentiation factor 15	-10.63	0.00361	-1.47	0.55723
**CHST11**	carbohydrate (chondroitin 4) sulfotransferase 11	-10.61	6.00E-05	-2.02	0.08082
**UGT8**	UDP glycosyltransferase 8	-10.45	1.00E-05	-1.06	0.83301
**IL1B**	interleukin 1, beta	-10.32	7.00E-05	-1.09	0.8173
**CDH11**	cadherin 11, type 2, OB-cadherin (osteoblast)	-10.27	0	-1.32	0.31101
**BC027448**	---	-10.18	0	1.04	0.85824
**HBEGF**	heparin-binding EGF-like growth factor	-10.15	3.00E-05	-1.33	0.40938
**MFSD2A**	major facilitator superfamily domain containing 2A	-10.08	0	-1.55	0.12522
**LOC100506870**	uncharacterized LOC100506870	-10.04	0.00016	-1.78	0.17859
**STC2**	stanniocalcin 2	-9.91	0.01089	1.11	0.88649
**LIF**	leukemia inhibitory factor	-9.77	4.00E-05	-1.16	0.67491
**HAL**	histidine ammonia-lyase	-9.33	0	1.16	0.27135
**PSAT1**	phosphoserine aminotransferase 1	-9.13	2.00E-04	-1.96	0.11804
**DMBT1**	deleted in malignant brain tumors 1	-8.39	5.00E-05	-1.02	0.95146
**CYP24A1**	cytochrome P450, family 24, subfamily A, polypeptide 1	-8.31	0.00013	-1.04	0.92083
**CHAC1**	ChaC, cation transport regulator homolog 1 (E. coli)	-8.30	0.0188	-1.3	0.73814
**PNMA2**	paraneoplastic Ma antigen 2	-8.08	3.00E-05	1.11	0.73676
**SLCO4A1**	solute carrier organic anion transporter family, member 4A1	-7.92	6.00E-05	-1.01	0.97645
**IL11**	interleukin 11	-7.62	7.00E-05	1.71	0.12442
**JAM3**	junctional adhesion molecule 3	-7.59	0	-1.14	0.56748
**PPP1R3C**	protein phosphatase 1, regulatory subunit 3C	-7.28	1.00E-05	-1.2	0.49605
**HPCAL4**	hippocalcin like 4	-7.20	0	-1.22	0.28761
**PTGS1**	prostaglandin-endoperoxide synthase 1 (prostaglandin G/H synthase and cyclooxygenase)	-7.16	9.00E-05	-1.89	0.07106
**DHRS2 ***	dehydrogenase/reductase (SDR family) member 2	-7.12	0.00051	4.02	0.00531
**ELOVL6**	ELOVL fatty acid elongase 6	-7.06	0	-1.34	0.16912
**CTSL2**	cathepsin L2	-6.95	0.00071	-1.21	0.65159

**IL8**	interleukin 8	-6.83	0	1.94	0.00775
**SERPINB9**	serpin peptidase inhibitor, clade B (ovalbumin), member 9	-6.82	0	-1.04	0.86809
**AREGB**	Amphiregulin B	-6.68	0	-1.07	0.74896
**FOS**	FBJ murine osteosarcoma viral oncogene homolog	-6.66	3.00E-05	-1.51	0.16137
**AI936034**	---	-6.65	6.00E-05	-1.06	0.83556
**SERPINB4**	serpin peptidase inhibitor, clade B (ovalbumin), member 4	-6.58	1.00E-05	-1.01	0.9801
**PLAU**	plasminogen activator, urokinase	-6.56	0	-1.04	0.81734
**ITGA4**	integrin, alpha 4 (antigen CD49D, alpha 4 subunit of VLA-4 receptor)	-6.51	0.00015	1.21	0.56536
**DUSP5**	dual specificity phosphatase 5	-6.50	0	1.09	0.68515
**AGPAT9**	1-acylglycerol-3-phosphate O-acyltransferase 9	-6.50	0.00013	-1.58	0.17957
**LOC541471**	uncharacterized LOC541471	-6.43	0	-1.14	0.28764
**KLF9**	Kruppel-like factor 9	-6.30	0.00242	-1.18	0.72463
**KYNU**	kynureninase	-6.23	0	-1.68	0.02918
**NR4A3**	nuclear receptor subfamily 4, group A, member 3	-6.19	3.00E-05	-1.2	0.48638
**SPRY4**	sprouty homolog 4 (Drosophila)	-6.03	0	-1.15	0.52646
**ABCC3**	ATP-binding cassette, sub-family C (CFTR/MRP), member 3	-5.95	4.00E-05	-1.67	0.07485
**CHL1-AS2**	CHL1 antisense RNA 2 (non-protein coding)	-5.91	2.00E-05	-1.23	0.405
**HIST1H3A**	histone cluster 1, H3a	-5.89	9.00E-05	-1.94	0.04225
**DESI1**	desumoylating isopeptidase 1	-5.86	5.00E-04	-3.64	0.00435
**KIF21B**	kinesin family member 21B	-5.83	1.00E-04	-1.36	0.3111
**KCNQ5**	potassium voltage-gated channel, KQT-like subfamily, member 5	-5.82	0	1.06	0.71397
**CA12**	carbonic anhydrase XII	-5.81	0.00027	1.15	0.67697
**TIMP1**	TIMP metallopeptidase inhibitor 1	-5.76	0.00025	-1.79	0.10017
**SH3GL3**	SH3-domain GRB2-like 3	-5.76	0.00054	1.16	0.68034
**AL049245**	---	-5.75	0	-1.05	0.7761
**PCOLCE2**	procollagen C-endopeptidase enhancer 2	-5.72	0	1.08	0.70474
**IL1A**	interleukin 1, alpha	-5.65	4.00E-05	1.14	0.62626
**F2RL2**	coagulation factor II (thrombin) receptor-like 2	-5.63	0.00428	1.59	0.35014
**TGM2**	transglutaminase 2 (C polypeptide, protein-glutamine-gamma-glutamyltransferase)	-5.62	0.00027	1.13	0.70417
**SPP1**	secreted phosphoprotein 1	-5.59	0.00028	1.03	0.93498
**ANO3**	anoctamin 3	-5.59	0	-1.5	0.05304
**LOC100653010**	uncharacterized LOC100653010	-5.58	0.02428	-1.45	0.57858
**FSTL1**	follistatin-like 1	-5.57	1.00E-05	-1.81	0.01931
**CBLC**	Cbl proto-oncogene, E3 ubiquitin protein ligase C	-5.55	1.00E-05	-1.19	0.41841
**IGFBP7**	insulin-like growth factor binding protein 7	-5.48	0	-1.54	0.02086
**CDC45**	cell division cycle 45 homolog (S. cerevisiae)	-5.43	0.00073	-1.18	0.65399
**FAM101B**	family with sequence similarity 101, member B	-5.41	0.00092	1.34	0.44382
**SAA1**	serum amyloid A1	-5.34	0.00149	-1.38	0.4275
**PECAM1**	platelet/endothelial cell adhesion molecule 1	-5.33	8.00E-05	-1.25	0.41692
**KIF15**	kinesin family member 15	-5.23	0.00205	-1.55	0.30368
**DUSP4**	dual specificity phosphatase 4	-5.23	0	-1.31	0.11602
**SKA3**	spindle and kinetochore associated complex subunit 3	-5.22	0.00665	-1.4	0.50876
**TRIB3**	tribbles homolog 3 (Drosophila)	-5.20	0.02592	-1.29	0.69487
**CTGF**	connective tissue growth factor	-5.16	2.00E-05	-1.18	0.49192
**BU955063**	---	-5.15	2.00E-04	1.01	0.97263

**FARP1**	FERM, RhoGEF (ARHGEF) and pleckstrin domain protein 1 (chondrocyte-derived)	-5.11	0	-1.4	0.09444
**FAM216A**	family with sequence similarity 216, member A	-5.08	0.00011	-1.73	0.06788
**HTRA3**	HtrA serine peptidase 3	-5.05	0	-1.2	0.30938
**TSPAN12**	tetraspanin 12	-5.05	0.01751	-2.85	0.09684
**MELK**	maternal embryonic leucine zipper kinase	-5.04	0.00751	-1.11	0.83668
**COL4A1**	collagen, type IV, alpha 1	-5.01	2.00E-05	-1.13	0.58963
**FGF2**	fibroblast growth factor 2 (basic)	-4.96	5.00E-05	1.1	0.70631
**RASD1**	RAS, dexamethasone-induced 1	-4.96	2.00E-05	1.05	0.8222
**SLC9A7**	solute carrier family 9, subfamily A (NHE7, cation proton antiporter 7), member 7	-4.92	0.00171	-1.03	0.94709
**BC043357**	---	-4.87	6.00E-05	-1.05	0.85858
**SERPINA3**	serpin peptidase inhibitor, clade A (alpha-1 antiproteinase, antitrypsin), member 3	-4.85	8.00E-04	-1.05	0.88585
**SFRP1**	secreted frizzled-related protein 1	-4.85	4.00E-05	-1.18	0.49084
**SYT12**	synaptotagmin XII	-4.85	1.00E-05	-1.3	0.21144
**TMCC3**	transmembrane and coiled-coil domain family 3	-4.85	0.00034	-1.71	0.10433
**EXO1**	exonuclease 1	-4.83	4.00E-05	-1.08	0.75247
**KCNG1**	potassium voltage-gated channel, subfamily G, member 1	-4.82	1.00E-05	-1.1	0.65882
**SLC1A4**	solute carrier family 1 (glutamate/neutral amino acid transporter), member 4	-4.81	0.00034	-1.22	0.52294
**CENPQ**	centromere protein Q	-4.80	0.00204	-1.59	0.25458
**PAQR5**	progestin and adipoQ receptor family member V	-4.77	7.00E-05	1.25	0.39312
**TGFB2**	transforming growth factor, beta 2	-4.76	0.00034	-1.21	0.53776
**CKAP2L**	cytoskeleton associated protein 2-like	-4.74	0.00111	-1.49	0.27735
**LRP8**	low density lipoprotein receptor-related protein 8, apolipoprotein e receptor	-4.73	0.00032	1.27	0.43833
**SPRY2**	sprouty homolog 2 (Drosophila)	-4.67	0.00054	-1.17	0.61829
**CSGALNACT1**	chondroitin sulfate N-acetylgalactosaminyltransferase 1	-4.67	0.00032	1.21	0.5261
**MICAL2**	microtubule associated monoxygenase, calponin and LIM domain containing 2	-4.65	0.0036	1.05	0.90275
**SERPINA1**	serpin peptidase inhibitor, clade A (alpha-1 antiproteinase, antitrypsin), member 1	-4.65	0.00044	-1.07	0.83686
**SLC9A1**	solute carrier family 9, subfamily A (NHE1, cation proton antiporter 1), member 1	-4.65	1.00E-05	-1.75	0.01078
**LOC100507307**	uncharacterized LOC100507307	-4.63	0	-1.47	0.05153
**PLEK2**	pleckstrin 2	-4.60	0.00022	1.47	0.19332
**ABCA12**	ATP-binding cassette, sub-family A (ABC1), member 12	-4.59	0.00018	-1.13	0.65965
**MIR17HG**	MIR17 host gene (non-protein coding)	-4.59	3.00E-05	-1.95	0.01178
**IL27RA**	interleukin 27 receptor, alpha	-4.54	0.00111	1.15	0.6816
**KCNK5**	potassium channel, subfamily K, member 5	-4.54	0.00062	-1.41	0.29856
**C11orf82**	chromosome 11 open reading frame 82	-4.53	0.00019	-1.39	0.25196
**IL20**	interleukin 20	-4.53	0.0017	-1.1	0.7965
**ADORA2B**	adenosine A2b receptor	-4.52	8.00E-05	-1.07	0.77319
**CLGN**	calmegin	-4.51	0.00913	1.02	0.97307
**CLDN10**	claudin 10	-4.50	0	-1.01	0.96027
**SGTB**	small glutamine-rich tetratricopeptide repeat (TPR)-containing, beta	-4.50	1.00E-05	-1.32	0.16415
**GALNT12**	UDP-N-acetyl-alpha-D-galactosamine:polypeptide N-acetylgalactosaminyltransferase 12 (GalNAc-T12)	-4.47	8.00E-05	-1.85	0.02656

**GRAMD1B**	GRAM domain containing 1B	-4.47	5.00E-05	1.26	0.33164
**CTH ***	cystathionase (cystathionine gamma-lyase)	-4.45	0.00113	2.38	0.0172
**MCM6**	minichromosome maintenance complex component 6	-4.45	0.00083	-1.08	0.81474
**MKI67**	antigen identified by monoclonal antibody Ki-67	-4.45	0.00115	-1.5	0.25198
**BC015429**	---	-4.39	0.00034	-1.07	0.82733
**PTPRE**	protein tyrosine phosphatase, receptor type, E	-4.38	1.00E-05	-1.23	0.2796
**SLC39A8**	solute carrier family 39 (zinc transporter), member 8	-4.36	7.00E-05	-1.68	0.04818
**C11orf86**	chromosome 11 open reading frame 86	-4.34	0.00013	1.03	0.89948
**ADAM23**	ADAM metallopeptidase domain 23	-4.34	0.00016	1.18	0.53925
**ASF1B**	ASF1 anti-silencing function 1 homolog B (S. cerevisiae)	-4.33	0.0113	1.37	0.52752
**RMI2**	RMI2, RecQ mediated genome instability 2, homolog (S. cerevisiae)	-4.33	0.00544	-1.02	0.95803
**STEAP4**	STEAP family member 4	-4.27	0.00091	-1.99	0.0536
**BEND6**	BEN domain containing 6	-4.27	2.00E-05	-1.42	0.10304
**DEPDC1**	DEP domain containing 1	-4.25	0.01146	-1.33	0.55778
**ZNF697**	zinc finger protein 697	-4.24	0.00231	-1.46	0.31807
**SORBS2**	sorbin and SH3 domain containing 2	-4.23	0.00779	-1.05	0.91115
**SLC2A3**	solute carrier family 2 (facilitated glucose transporter), member 3	-4.22	0.00034	1.45	0.20854
**TPRG1**	tumor protein p63 regulated 1	-4.22	0.00033	1.2	0.51485
**PHF20L1**	PHD finger protein 20-like 1	-4.19	0.01178	-1.7	0.28264
**HIST1H1D**	histone cluster 1, H1d	-4.18	0	-1.07	0.67885
**PHGDH**	phosphoglycerate dehydrogenase	-4.18	0.00044	1.06	0.83981
**DLGAP5**	discs, large (Drosophila) homolog-associated protein 5	-4.17	0.02027	-1.76	0.30433
**GJA3**	gap junction protein, alpha 3, 46kDa	-4.17	0.01423	-1.11	0.83517
**CHSY3**	chondroitin sulfate synthase 3	-4.16	0.00028	-1.13	0.65437
**LUM**	lumican	-4.16	0.03358	-1.33	0.63405
**MND1**	meiotic nuclear divisions 1 homolog (S. cerevisiae)	-4.16	1.00E-05	-1.35	0.11375
**TRAF5**	TNF receptor-associated factor 5	-4.14	0	1.62	0.00257
**HOXD-AS1**	HOXD cluster antisense RNA 1 (non-protein coding)	-4.14	0.00449	-2.62	0.03358
**XPR1**	xenotropic and polytropic retrovirus receptor 1	-4.14	0.00657	-1.47	0.37781
**LAPTM5**	lysosomal protein transmembrane 5	-4.14	0	-1.03	0.80679
**BCL3**	B-cell CLL/lymphoma 3	-4.12	1.00E-05	-1.06	0.72639
**FNDC3B**	fibronectin type III domain containing 3B	-4.12	0.00773	-1.92	0.15967
**GPT2**	glutamic pyruvate transaminase (alanine aminotransferase) 2	-4.10	0.00012	-1.01	0.95903
**ERRFI1**	ERBB receptor feedback inhibitor 1	-4.08	1.00E-04	-1.84	0.02463
**CDT1**	Chromatin licensing and DNA replication factor 1	-4.08	0.02336	-1.17	0.76838
**EGR1**	early growth response 1	-4.08	1.00E-05	-2.26	0.00114
**DKK1**	dickkopf 1 homolog (Xenopus laevis)	-4.06	0.00966	-1.56	0.33531
**AA938184**	---	-4.06	0.02191	1.07	0.90576
**BF590917**	---	-4.06	1.00E-04	1.02	0.9321
**UBE2V2**	ubiquitin-conjugating enzyme E2 variant 2	-4.05	0.00781	-3.57	0.01315
**ALDH1A3**	aldehyde dehydrogenase 1 family, member A3	-4.04	0.00105	-1.29	0.4377
**SLCO2A1**	solute carrier organic anion transporter family, member 2A1	-4.04	0.00095	-1.35	0.34924
**CDCA7**	cell division cycle associated 7	-4.03	0.00118	-1.11	0.738
**RNF165**	ring finger protein 165	-4.02	1.00E-05	-1.76	0.01058

**FOSL1**	FOS-like antigen 1	-4.01	0.00018	-1.26	0.36281
**PSRC1**	proline/serine-rich coiled-coil 1	-4.01	0.00592	-2.11	0.0922
**IER3**	immediate early response 3	-4.00	0	-1.1	0.55523
**MAP1B**	microtubule-associated protein 1B	-4.00	0.00822	1.16	0.73494
**VLDLR**	very low density lipoprotein receptor	-3.99	0.00422	-1	0.99744
**DLGAP1-AS2**	DLGAP1 antisense RNA 2	-3.99	9.00E-05	-1.57	0.06964
**AI926924**	---	-3.93	0.01003	-1.46	0.40675
**ZNF239**	zinc finger protein 239	-3.92	0.00015	-1.19	0.48845
**JPH1**	junctophilin 1	-3.92	0.0011	1.25	0.48168
**CHRM3**	cholinergic receptor, muscarinic 3	-3.92	0.00085	-1.07	0.83136
**BLMH**	bleomycin hydrolase	-3.91	1.00E-04	-1.29	0.27753
**SH3KBP1**	SH3-domain kinase binding protein 1	-3.90	0.00018	-1.27	0.34487
**SHISA2**	shisa homolog 2 (Xenopus laevis)	-3.89	0.00035	1.14	0.61964
**KLHL8**	kelch-like 8 (Drosophila)	-3.89	0.00637	-2.16	0.0815
**MAP3K9**	mitogen-activated protein kinase kinase kinase 9	-3.88	1.00E-04	1.02	0.93607
**MARC1**	mitochondrial amidoxime reducing component 1	-3.88	0.00127	-1.15	0.66513
**FYN**	FYN oncogene related to SRC, FGR, YES	-3.87	0.00025	-1.11	0.69561
**NR4A2 ***	nuclear receptor subfamily 4, group A, member 2	-3.86	0.00041	2.17	0.01446
**SLC29A1**	solute carrier family 29 (nucleoside transporters), member 1	-3.85	0.00018	1.09	0.72033
**TNFRSF12A**	tumor necrosis factor receptor superfamily, member 12A	-3.85	0.00162	-1.35	0.36653
**AI743753**	---	-3.85	0.00102	-1.93	0.05111
**GLB1L2**	galactosidase, beta 1-like 2	-3.83	0.00101	-1.26	0.45423
**EIF4EBP1**	eukaryotic translation initiation factor 4E binding protein 1	-3.83	0.00085	-1.17	0.59764
**RAD51AP1**	RAD51 associated protein 1	-3.82	0.03328	1.17	0.77625
**NTN4**	netrin 4	-3.82	0.001	-1.95	0.04646
**GEM**	GTP binding protein overexpressed in skeletal muscle	-3.82	0.00416	-1.02	0.94967
**TRPA1**	transient receptor potential cation channel, subfamily A, member 1	-3.81	0.0023	-1.08	0.81279
**PALD1**	phosphatase domain containing, paladin 1	-3.81	3.00E-05	1.2	0.34561
**PGF**	placental growth factor	-3.81	0.00459	-1.13	0.74268
**BQ024490**	---	-3.80	0.00026	-1.14	0.60793
**FAM132B**	family with sequence similarity 132, member B	-3.79	0.00015	-1.03	0.89889
**SEMA3G**	sema domain, immunoglobulin domain (Ig), short basic domain, secreted, (semaphorin) 3G	-3.79	0.00491	1.06	0.8723
**CDCA5**	cell division cycle associated 5	-3.78	0.04824	-1.29	0.67428
**LAMA1**	laminin, alpha 1	-3.78	7.00E-05	1.06	0.7756
**CHST6**	carbohydrate (N-acetylglucosamine 6-O) sulfotrans	-3.75	0.00028	-1.61	0.08158
**ZNF331**	zinc finger protein 331	-3.73	1.00E-05	-1.04	0.79857
**PRKCH**	protein kinase C, eta	-3.73	0.00024	1.22	0.42117
**LPAR3**	lysophosphatidic acid receptor 3	-3.71	1.00E-05	-1.1	0.54784
**LOC100507564**	uncharacterized LOC100507564	-3.70	0.00018	1.13	0.59692
**SARS**	seryl-tRNA synthetase	-3.69	0.00314	-1.13	0.72791
**ARL13B**	ADP-ribosylation factor-like 13B	-3.68	0.0106	-1.72	0.22489
**CTNNAL1**	catenin (cadherin-associated protein), alpha-like 1	-3.68	0.0018	1.21	0.55403
**E2F8**	E2F transcription factor 8	-3.68	0.00376	-1.04	0.91717
**NID2**	nidogen 2 (osteonidogen)	-3.68	0.00591	1	0.99963
**NOG**	noggin	-3.67	0.00015	-1.27	0.31226
**PRKAR2B**	protein kinase, cAMP-dependent, regulatory, type II, beta	-3.67	0.02668	1.46	0.47116
**UBASH3B**	ubiquitin associated and SH3 domain containing B	-3.66	3.00E-05	1.44	0.07612

**PBK**	PDZ binding kinase	-3.65	0.03004	-1.15	0.79546
**LGSN**	lengsin, lens protein with glutamine synthetase domain	-3.65	0.02779	1.01	0.98181
**DEPDC1B**	DEP domain containing 1B	-3.65	0.02583	-1.3	0.60783
**LOC100506918**	hypothetical LOC100506918	-3.63	0.00107	-1.82	0.06307
**SPAG4**	sperm associated antigen 4	-3.62	0.00425	-1.03	0.92528
**TCF19**	transcription factor 19	-3.62	0.017	1.17	0.73299
**EGF**	epidermal growth factor	-3.62	4.00E-05	-1.42	0.08946
**NUF2**	NUF2, NDC80 kinetochore complex component, homolog (S. cerevisiae)	-3.61	0.0157	-1.3	0.57088
**OSMR**	oncostatin M receptor	-3.60	0.00092	-1.21	0.50367
**LINC00473**	long intergenic non-protein coding RNA 473	-3.60	6.00E-05	-1.51	0.06267
**STK32C**	serine/threonine kinase 32C	-3.60	0.00125	-1.6	0.13821
**MCM10**	minichromosome maintenance complex component 10	-3.59	8.00E-05	-1.67	0.02965
**BG054960**	---	-3.59	0.00026	-1.39	0.19663
**LYPD6B**	LY6/PLAUR domain containing 6B	-3.58	1.00E-05	1.23	0.19594
**MTHFD1L**	methylenetetrahydrofolate dehydrogenase (NADP+ dependent) 1-like	-3.57	7.00E-05	-1.07	0.74611
**DNASE1L3**	deoxyribonuclease I-like 3	-3.56	0.00101	-1.37	0.29023
**AI806183**	---	-3.55	0.00021	1.05	0.8325
**CCNYL1**	cyclin Y-like 1	-3.54	0.00777	-1.03	0.93456
**SLC7A11**	solute carrier family 7 (anionic amino acid transporter light chain, xc- system), member 11	-3.54	0.00907	1.06	0.88323
**KIAA1644**	KIAA1644	-3.53	1.00E-05	-1.33	0.08499
**SH3PXD2B**	SH3 and PX domains 2B	-3.52	0.00383	1.43	0.31562
**UBE2C**	ubiquitin-conjugating enzyme E2C	-3.51	0.00696	-1.63	0.22187
**S100A9**	S100 calcium binding protein A9	-3.51	0.00015	-1.11	0.63018
**BF590850**	---	-3.50	0.00171	-1.23	0.50862
**FAS**	Fas (TNF receptor superfamily, member 6)	-3.50	0.00021	-1.17	0.51164
**NEIL3**	nei endonuclease VIII-like 3 (E. coli)	-3.50	4.00E-04	-1.69	0.05716
**SAA2**	serum amyloid A2	-3.49	8.00E-05	-1.76	0.01761
**LEPROTL1**	leptin receptor overlapping transcript-like 1	-3.49	0	-1.14	0.36824
**SMC2**	structural maintenance of chromosomes 2	-3.49	0.0155	1.09	0.85304
**MGC20647**	uncharacterized protein MGC20647	-3.48	7.00E-05	-1.18	0.41407
**AKAP12**	A kinase (PRKA) anchor protein 12	-3.48	0	1.41	0.03918
**NCAM1**	neural cell adhesion molecule 1	-3.48	0.00082	1.06	0.823
**AURKB**	aurora kinase B	-3.47	0.02316	-1.42	0.47211
**SSH1**	slingshot homolog 1 (Drosophila)	-3.47	0.00743	1.13	0.74807
**NCAPH**	non-SMC condensin I complex, subunit H	-3.47	0.03792	-1.47	0.47798
**SPDL1**	spindle apparatus coiled-coil protein 1	-3.45	0.00167	-1.41	0.27314
**BG112263**	---	-3.45	1.00E-04	-1.52	0.06324
**RAB3B**	RAB3B, member RAS oncogene family	-3.43	0.00945	1	0.99046
**IL24**	interleukin 24	-3.43	0.04046	1.31	0.6167
**ABCC4**	ATP-binding cassette, sub-family C (CFTR/MRP), member 4	-3.42	0.00018	-1.19	0.44815
**CXCL2**	chemokine (C-X-C motif) ligand 2	-3.41	0.0041	-1.58	0.2029
**SPC25**	SPC25, NDC80 kinetochore complex component, homolog (S. cerevisiae)	-3.41	0.00082	-1.42	0.21554
**CLDN23**	claudin 23	-3.41	0.00133	-1.69	0.09183
**BC020911**	---	-3.41	0.00068	-1.19	0.50858
**CA2**	carbonic anhydrase II	-3.40	0.01952	1.13	0.78525
**TOX2**	TOX high mobility group box family member 2	-3.40	0.0143	1.15	0.73783
**C1orf112**	chromosome 1 open reading frame 112	-3.39	0.00114	-1.1	0.72907

**CENPV**	centromere protein V	-3.39	0.00011	1.1	0.63599
**GALNT6**	UDP-N-acetyl-alpha-D-galactosamine:polypeptide N-acetylgalactosaminyltransferase 6 (GalNAc-T6)	-3.39	0.00014	1.14	0.54046
**FEZ1**	fasciculation and elongation protein zeta 1 (zygin I)	-3.39	0.00021	1.04	0.86559
**ADD2**	adducin 2 (beta)	-3.39	0.00129	-1.68	0.09371
**CTNNB1**	catenin (cadherin-associated protein), beta 1, 88kDa	-3.38	1.00E-04	-1.15	0.49464
**FAM83A**	family with sequence similarity 83, member A	-3.38	0.00279	-1.29	0.43941
**HK2**	hexokinase 2	-3.37	0	-1.18	0.16748
**PGM2**	phosphoglucomutase 2	-3.37	0.00069	-1.56	0.11362
**CEP128**	centrosomal protein 128kDa	-3.37	0.00173	-1.52	0.17622
**FAM46C**	family with sequence similarity 46, member C	-3.36	0.00079	-1.74	0.05686
**SMAD3**	SMAD family member 3	-3.36	0.00017	-1.42	0.12612
**CNTNAP2**	contactin associated protein-like 2	-3.36	0.00154	1.05	0.87244
**INPP4B**	inositol polyphosphate-4-phosphatase, type II, 105kDa	-3.36	0	-1.37	0.01381
**CDC25C**	cell division cycle 25 homolog C (S. pombe)	-3.35	0.00603	-1.72	0.15227
**SLC17A9**	solute carrier family 17, member 9	-3.35	9.00E-05	-1.23	0.31178
**PRSS12**	protease, serine, 12 (neurotrypsin, motopsin)	-3.35	0.03186	1.32	0.58368
**CCNA2**	cyclin A2	-3.35	0.03142	-1.25	0.66059
**PMAIP1**	phorbol-12-myristate-13-acetate-induced protein 1	-3.35	0.00146	1.48	0.19682
**NUP210**	nucleoporin 210kDa	-3.33	6.00E-05	-1.24	0.2806
**HJURP**	Holliday junction recognition protein	-3.33	0.01518	-1.71	0.22348
**RUNX1**	runt-related transcription factor 1	-3.32	0.00111	-1.15	0.61944
**AI435073**	---	-3.32	9.00E-04	-1.26	0.39973
**LOC100506860**	uncharacterized LOC100506860	-3.31	0	-1.46	0.01351
**ERVMER34-1**	endogenous retrovirus group MER34, member 1	-3.30	0.00084	-1.09	0.75541
**CEP55**	centrosomal protein 55kDa	-3.30	0.00328	-1.38	0.33236
**CA9**	carbonic anhydrase IX	-3.29	0.03444	-1.85	0.23587
**THBD**	thrombomodulin	-3.29	0.00486	1.05	0.88989
**TPBG**	trophoblast glycoprotein	-3.29	8.00E-05	-1.15	0.4715
**CCNB1**	cyclin B1	-3.29	0.00812	-1.39	0.39181
**SCLY**	selenocysteine lyase	-3.28	0.015	1.31	0.52706
**TK1**	thymidine kinase 1, soluble	-3.28	0.01742	-1.25	0.61055
**ABCG1**	ATP-binding cassette, sub-family G (WHITE), member 1	-3.28	0.00195	-1.08	0.79403
**SERPINB1**	serpin peptidase inhibitor, clade B (ovalbumin), member 1	-3.28	1.00E-05	-1.65	0.00514
**NDRG4**	NDRG family member 4	-3.28	0.00201	-1.19	0.56409
**TMEM51**	transmembrane protein 51	-3.28	1.00E-05	-1.32	0.08556
**ATAD2**	ATPase family, AAA domain containing 2	-3.28	0.03566	1.28	0.62544
**NPHP1**	nephronophthisis 1 (juvenile)	-3.27	3.00E-05	-1.47	0.0444
**HCAR3**	hydroxycarboxylic acid receptor 3	-3.27	7.00E-05	-1.06	0.76753
**HIP1**	huntingtin interacting protein 1	-3.27	0.00596	1.04	0.90959
**AW009630**	---	-3.27	0.00148	1.16	0.6038
**ARHGAP29**	Rho GTPase activating protein 29	-3.26	2.00E-05	-1.01	0.94795
**FUT11**	fucosyltransferase 11 (alpha (1,3) fucosyltransferase)	-3.26	0.01646	-1.66	0.2487
**LINC00622**	long intergenic non-protein coding RNA 622	-3.26	0.00199	-1.14	0.65081
**HPSE**	heparanase	-3.26	0.00119	1.89	0.03772
**BF056892**	---	-3.26	0.00166	-1.31	0.36374

**GPD2**	glycerol-3-phosphate dehydrogenase 2 (mitochondrial)	-3.26	0.0029	1.15	0.65494
**RAET1E**	retinoic acid early transcript 1E	-3.26	3.00E-05	-1.04	0.8185
**BUB1B**	budding uninhibited by benzimidazoles 1 homolog beta (yeast)	-3.26	0.00624	-1.1	0.78182
**STAC**	SH3 and cysteine rich domain	-3.26	0.00029	-1.2	0.43036
**RASGEF1A**	RasGEF domain family, member 1A	-3.26	0.00165	1.93	0.04032
**CDKN3**	cyclin-dependent kinase inhibitor 3	-3.25	0.00112	-1.51	0.15075
**KIF5C**	kinesin family member 5C	-3.25	0.00202	1.08	0.79614
**SRPX**	sushi-repeat containing protein, X-linked	-3.25	0.00184	1.19	0.54965
**AK022474**	---	-3.25	0.00018	1.01	0.95255
**MAFF**	v-maf musculoaponeurotic fibrosarcoma oncogene homolog F (avian)	-3.25	4.00E-05	1.2	0.31914
**MFI2**	antigen p97 (melanoma associated) identified by monoclonal antibodies 133.2 and 96.5	-3.25	0.00137	-1.07	0.79867
**H1F0**	H1 histone family, member 0	-3.24	7.00E-05	-1.53	0.04592
**JKAMP**	JNK1/MAPK8-associated membrane protein	-3.22	0.03274	-1.49	0.42179
**SPRY4-IT1**	SPRY4 intronic transcript 1 (non-protein coding)	-3.22	0.03184	1.18	0.73759
**CDC6**	cell division cycle 6 homolog (S. cerevisiae)	-3.22	4.00E-05	-1.01	0.96389
**CDCP1**	CUB domain containing protein 1	-3.21	0.00111	-1.52	0.14136
**RGCC**	regulator of cell cycle	-3.21	0.02073	1.1	0.82487
**ANGPTL4**	angiopoietin-like 4	-3.21	0.00597	-1.22	0.5731
**AI640482**	---	-3.20	0.00033	1	0.99189
**S100A3**	S100 calcium binding protein A3	-3.20	0.00835	1.29	0.49349
**ENO3**	enolase 3 (beta, muscle)	-3.19	8.00E-05	1.41	0.09206
**SRGN**	serglycin	-3.18	3.00E-04	-1.02	0.93817
**AK022350**	---	-3.18	0.02706	1.14	0.78143
**N69098**	---	-3.18	0.00039	-1.03	0.89821
**IL4R**	interleukin 4 receptor	-3.18	1.00E-04	-1.21	0.33246
**GJB2**	gap junction protein, beta 2, 26kDa	-3.18	0.00253	1.35	0.32958
**EGR2**	early growth response 2	-3.17	6.00E-05	-1.27	0.20255
**PDP1**	pyruvate dehyrogenase phosphatase catalytic subunit 1	-3.17	0.0225	1.14	0.76302
**SIX1**	SIX homeobox 1	-3.17	0.00489	1.49	0.24833
**SPRED1**	sprouty-related, EVH1 domain containing 1	-3.16	0.00092	-1.21	0.47189
**AI088104**	---	-3.16	0.00791	-1.07	0.85801
**RASL11B**	RAS-like, family 11, member B	-3.16	0.00068	-1.06	0.82468
**PRPS1**	phosphoribosyl pyrophosphate synthetase 1	-3.16	0.00463	1.03	0.91909
**RBM24**	RNA binding motif protein 24	-3.16	0.00474	-1.3	0.43812
**TIAM1**	T-cell lymphoma invasion and metastasis 1	-3.16	0.00886	1.03	0.93353
**TEX30**	testis expressed 30	-3.15	0.00311	1.28	0.42589
**DDX28**	DEAD (Asp-Glu-Ala-Asp) box polypeptide 28	-3.15	0.02276	-2.19	0.09709
**PRIMA1**	proline rich membrane anchor 1	-3.15	0.00259	-1.13	0.69479
**GPRC5B**	G protein-coupled receptor, family C, group 5, member B	-3.15	0.00234	-1.9	0.04762
**IL36G**	interleukin 36, gamma	-3.14	0.00053	-1.11	0.64851
**FZD5**	frizzled family receptor 5	-3.14	0.00251	-1.25	0.46484
**AI761207**	---	-3.14	0.00697	1.45	0.29651
**LYPD6**	LY6/PLAUR domain containing 6	-3.14	0.00333	1.04	0.9077
**CHRNA5**	cholinergic receptor, nicotinic, alpha 5 (neuronal)	-3.13	0.00119	1.53	0.12825
**PCSK9**	proprotein convertase subtilisin/kexin type 9	-3.13	0.00098	-1.23	0.4326
**PAQR4**	progestin and adipoQ receptor family member IV	-3.13	0.00377	1.09	0.78133
**KIF23**	kinesin family member 23	-3.12	0.02753	-1.37	0.49418
**GCH1**	GTP cyclohydrolase 1	-3.12	0.00027	-1.07	0.74815

**ODF2**	outer dense fiber of sperm tails 2	-3.12	0.02259	1.05	0.90229
**AW293316**	---	-3.12	0.02818	-1.54	0.35271
**DTL**	denticleless E3 ubiquitin protein ligase homolog (Drosophila)	-3.11	0.0014	1.38	0.2484
**C18orf54**	chromosome 18 open reading frame 54	-3.11	0.00456	-1.27	0.46304
**AI809536**	---	-3.10	8.00E-05	-1.64	0.01949
**PFAS**	phosphoribosylformylglycinamidine synthase	-3.09	0.00084	-1.12	0.64607
**SLC6A15**	solute carrier family 6 (neutral amino acid transporter), member 15	-3.09	0.00581	-1.07	0.83558
**WDHD1**	WD repeat and HMG-box DNA binding protein 1	-3.08	0.00629	1.26	0.50069
**CBS**	cystathionine-beta-synthase	-3.08	0.01815	1.31	0.51184
**TSLP**	thymic stromal lymphopoietin	-3.08	0.00553	-1.27	0.46889
**CDCA3**	cell division cycle associated 3	-3.08	0.01976	-2.16	0.087
**TRMU**	tRNA 5-methylaminomethyl-2-thiouridylate methyltransferase	-3.08	0.04821	-1.11	0.83775
**AIF1L**	allograft inflammatory factor 1-like	-3.08	0.00021	1.81	0.01437
**S1PR3**	sphingosine-1-phosphate receptor 3	-3.08	0.00619	-1.15	0.67266
**DBF4B**	DBF4 homolog B (S. cerevisiae)	-3.08	0.02536	-1.31	0.54092
**E2F7**	E2F transcription factor 7	-3.07	0.0013	1.32	0.30531
**WWTR1**	WW domain containing transcription regulator 1	-3.07	0.00011	-1.38	0.1171
**SCNN1A**	sodium channel, non-voltage-gated 1 alpha subunit	-3.07	0.00038	1.56	0.06739
**TDRKH**	tudor and KH domain containing	-3.07	0.01686	1.03	0.94669
**DPH3**	DPH3, KTI11 homolog (S. cerevisiae)	-3.07	0.03076	-2.1	0.12742
**CENPF**	centromere protein F, 350/400kDa (mitosin)	-3.06	0.00437	-1.23	0.51816
**ARHGAP19**	Rho GTPase activating protein 19	-3.06	0.00498	-1.73	0.11234
**C21orf58**	chromosome 21 open reading frame 58	-3.06	0.0065	-1.01	0.98383
**FAM27E3**	family with sequence similarity 27, member E3	-3.06	0.00093	-1.54	0.10883
**AI701591**	---	-3.06	0.0229	-1.66	0.25507
**TTK**	TTK protein kinase	-3.05	0.03161	-1.4	0.47166
**GDF11**	growth differentiation factor 11	-3.05	0.00546	-1.95	0.06114
**AI476341**	---	-3.04	5.00E-05	1.03	0.85934
**HSPA12A**	heat shock 70kDa protein 12A	-3.04	0.00543	1.48	0.24674
**CYR61**	cysteine-rich, angiogenic inducer, 61	-3.04	0.03887	1.09	0.85417
**LCN2**	lipocalin 2	-3.04	0.01076	-1.37	0.40369
**HAUS7**	HAUS augmin-like complex, subunit 7	-3.03	0.00015	-1.04	0.8597
**ICAM3**	intercellular adhesion molecule 3	-3.03	0.00453	-1.16	0.63775
**TFPI2**	tissue factor pathway inhibitor 2	-3.03	0.00042	-1.31	0.23977
**SLC44A3**	solute carrier family 44, member 3	-3.03	0.00066	-1.65	0.05393
**NELL2**	NEL-like 2 (chicken)	-3.02	0.00575	1	0.99726
**WRB**	tryptophan rich basic protein	-3.02	3.00E-04	-1.64	0.03706
**ARPP19**	cAMP-regulated phosphoprotein, 19kDa	-3.02	0.03013	-1.66	0.27516
**AI085377**	---	-3.02	0.00137	1.08	0.78136
**MTHFD2**	methylenetetrahydrofolate dehydrogenase (NADP+ dependent) 2, methenyltetrahydrofolate cyclohydrolase	-3.01	0.00323	1.17	0.59388
**FXYD6**	FXYD domain containing ion transport regulator 6	-3.01	0.00057	1.19	0.46711
**FAM89A**	family with sequence similarity 89, member A	-3.01	0.00233	1.04	0.87931
**BMP6**	bone morphogenetic protein 6	-3.00	4.00E-05	1.05	0.77486
**XDH**	xanthine dehydrogenase	-3.00	0.00331	-1.08	0.80158
**KIAA1549**	KIAA1549	-3.00	0	-1.89	0.00042
**QSOX2**	quiescin Q6 sulfhydryl oxidase 2	-3.00	1.00E-05	-1.63	0.0033
**VSIG1**	V-set and immunoglobulin domain containing 1	-3.00	0.00036	-1.08	0.73456
**GLIS3**	GLIS family zinc finger 3	-3.00	0.00035	-1.25	0.31486

**KIF14**	kinesin family member 14	-3.00	0.01018	-1.25	0.53575
**AMIGO2**	adhesion molecule with Ig-like domain 2	-2.99	0.00507	1.25	0.48514
**C15orf23**	chromosome 15 open reading frame 23	-2.99	0.00277	-1.28	0.4019
**HEATR5A**	HEAT repeat containing 5A	-2.99	0.01051	-2.03	0.07101
**ERLIN1**	ER lipid raft associated 1	-2.98	0.01059	-1.25	0.53596
**PI3**	peptidase inhibitor 3, skin-derived	-2.98	0.00364	-1.99	0.0399
**UGGT2**	UDP-glucose glycoprotein glucosyltransferase 2	-2.98	0.02363	-2	0.12313
**RGS20**	regulator of G-protein signaling 20	-2.98	0.00078	1.08	0.73511
**WDR76**	WD repeat domain 76	-2.98	0.00247	1.52	0.15917
**ETV5**	Ets variant 5	-2.97	5.00E-05	-1.18	0.34541
**CCNE1**	cyclin E1	-2.96	6.00E-05	1.74	0.00792
**TMTC1**	transmembrane and tetratricopeptide repeat containing 1	-2.96	0.00058	-1	0.99439
**SYT17**	synaptotagmin XVII	-2.96	0.00013	1.01	0.9549
**AI494113**	---	-2.96	2.00E-05	-1.01	0.9727
**BF447286**	---	-2.96	0.01319	-2.01	0.08345
**AW874669**	---	-2.96	0.02715	-1.48	0.37096
**AW132066**	---	-2.96	0.00756	-1.47	0.26977
**TPX2**	TPX2, microtubule-associated, homolog (Xenopus laevis)	-2.95	0.01262	-1.25	0.55424
**FST ***	follistatin	-2.95	0.00143	2.30	0.01951
**PPM1E**	protein phosphatase, Mg2+/Mn2+ dependent, 1E	-2.95	0.00011	-1.42	0.08189
**SERPINE1**	serpin peptidase inhibitor, clade E (nexin, plasminogen activator inhibitor type 1), member 1	-2.95	0.03059	1.05	0.9123
**LRRC8B**	leucine rich repeat containing 8 family, member B	-2.95	0.00057	-1.34	0.21599
**SLC4A7**	solute carrier family 4, sodium bicarbonate cotransporter, member 7	-2.94	0	-1.43	0.00962
**RBMS3**	RNA binding motif, single stranded interacting protein 3	-2.94	6.00E-05	1.2	0.28816
**OSBP2**	oxysterol binding protein 2	-2.94	0.00121	-1.14	0.60473
**TMEM107**	transmembrane protein 107	-2.93	0.03492	1.06	0.90022
**BIRC5**	baculoviral IAP repeat containing 5	-2.93	0.01501	-1.48	0.31013
**GRB14**	growth factor receptor-bound protein 14	-2.93	1.00E-05	1.08	0.54709
**AV700891**	---	-2.93	2.00E-05	-1.05	0.7346
**AA010315**	---	-2.93	0.01968	2.03	0.09937
**TSC22D1**	TSC22 domain family, member 1	-2.93	8.00E-05	-1.18	0.36
**CAMK4**	calcium/calmodulin-dependent protein kinase IV	-2.93	8.00E-04	-1.21	0.42432
**AK026659**	---	-2.93	0.0218	1.4	0.41439
**C11orf24**	chromosome 11 open reading frame 24	-2.93	0.00014	-1.27	0.22457
**ATP8B2**	ATPase, aminophospholipid transporter, class I, type 8B, member 2	-2.92	0.01421	1.38	0.39641
**CHL1**	cell adhesion molecule with homology to L1CAM (close homolog of L1)	-2.92	0.03528	1.2	0.68595
**DARS2**	aspartyl-tRNA synthetase 2, mitochondrial	-2.92	0.00431	-1.22	0.51669
**SMOC1**	SPARC related modular calcium binding 1	-2.92	0.00125	-1.32	0.28696
**HAUS6**	HAUS augmin-like complex, subunit 6	-2.92	0.01471	1.25	0.56259
**SMAGP**	small cell adhesion glycoprotein	-2.92	8.00E-04	-1.3	0.28407
**CDK1**	cyclin-dependent kinase 1	-2.92	0.00454	-1.23	0.49924
**NFATC2**	nuclear factor of activated T-cells, cytoplasmic, calcineurin-dependent 2	-2.92	0.00996	-1.5	0.26124
**FAM213B**	family with sequence similarity 213, member B	-2.92	0.00125	-1.33	0.26472
**PRMT3**	protein arginine methyltransferase 3	-2.91	0.01206	-1.07	0.86065
**OAS3**	2'-5'-oligoadenylate synthetase 3, 100kDa	-2.91	0.00226	-1.04	0.88464

**HEYL**	hairy/enhancer-of-split related with YRPW motif-like	-2.91	0.00946	-1.76	0.12237
**ZNRF3**	zinc and ring finger 3	-2.91	0.00602	-1.81	0.08326
**TTF2**	transcription termination factor, RNA polymerase II	-2.90	0.00899	-1.21	0.57169
**PCDHGC3**	protocadherin gamma subfamily C, 3	-2.90	0.0178	1.45	0.34611
**AI936724**	---	-2.90	0.00923	-1.74	0.12796
**FAM72A**	family with sequence similarity 72, member A	-2.90	0.00093	-1.39	0.18827
**CENPO**	centromere protein O	-2.89	0.00093	1.49	0.11519
**LOC100506844**	uncharacterized LOC100506844	-2.89	0	-1.56	0.00279
**DGKG**	diacylglycerol kinase, gamma 90kDa	-2.89	0.0097	-1.31	0.4338
**RSL1D1**	ribosomal L1 domain containing 1	-2.88	0.00529	-1.54	0.18137
**ATP6V1C1**	ATPase, H+ transporting, lysosomal 42kDa, V1 subunit C1	-2.88	0.03011	-2.3	0.07575
**HSPA2**	heat shock 70kDa protein 2	-2.88	0.01408	1.09	0.81676
**MMD**	monocyte to macrophage differentiation-associated	-2.88	0	1.71	0.00105
**IFITM1**	interferon induced transmembrane protein 1	-2.87	0.00016	-1.15	0.46912
**RAB31**	RAB31, member RAS oncogene family	-2.87	1.00E-05	-1.4	0.03554
**S1PR1**	sphingosine-1-phosphate receptor 1	-2.87	0.00223	-1.07	0.81175
**RAB38**	RAB38, member RAS oncogene family	-2.87	0.00285	-1.31	0.34402
**PLAUR**	plasminogen activator, urokinase receptor	-2.87	0.00036	1.39	0.13881
**CD58**	CD58 molecule	-2.86	0.00297	-1.41	0.2358
**EDNRB**	endothelin receptor type B	-2.86	0.02402	1.23	0.61451
**KIAA1549L**	KIAA1549-like	-2.86	4.00E-05	1.06	0.70448
**C9orf89**	chromosome 9 open reading frame 89	-2.86	0.02207	-2.26	0.06227
**R41296**	---	-2.86	0.00212	-1.22	0.46582
**SPAG5**	sperm associated antigen 5	-2.85	0.02843	-1.22	0.64436
**CALCA**	calcitonin-related polypeptide alpha	-2.85	0.0117	-1.01	0.97185
**TMEM194A**	transmembrane protein 194A	-2.85	5.00E-05	1.01	0.95389
**EFHD2**	EF-hand domain family, member D2	-2.85	0.00821	-1.19	0.59714
**RGS16**	regulator of G-protein signaling 16	-2.84	0.00517	-1.54	0.17751
**TNFSF10**	tumor necrosis factor (ligand) superfamily, member 10	-2.84	0.00748	-1.21	0.55131
**CCSAP**	centriole, cilia and spindle-associated protein	-2.84	0.00096	1.11	0.6664
**CYP27B1**	cytochrome P450, family 27, subfamily B, polypeptide 1	-2.83	0.01374	-1.39	0.37589
**SKA1**	spindle and kinetochore associated complex subunit 1	-2.83	0.01	1.05	0.88772
**LEPREL1**	leprecan-like 1	-2.83	5.00E-05	-1.97	0.00139
**SEMA4A**	sema domain, immunoglobulin domain (Ig), transmembrane domain (TM) and short cytoplasmic domain, (semaphorin) 4A	-2.83	0.00365	-1.04	0.88475
**NALCN**	sodium leak channel, non-selective	-2.83	0.00227	1.2	0.49713
**STAT4**	signal transducer and activator of transcription 4	-2.83	0.00011	-1.28	0.18452
**AIDA**	axin interactor, dorsalization associated	-2.83	0.00041	-1.81	0.01487
**FOXD2-AS1**	FOXD2 antisense RNA 1	-2.83	0.01082	1.06	0.87496
**NDC80**	NDC80 kinetochore complex component homolog (S. cerevisiae)	-2.82	0.00164	-1.23	0.42704
**AW117383**	---	-2.82	0.00021	1.08	0.6898
**PHLDA2**	pleckstrin homology-like domain, family A, member 2	-2.82	0.00951	1.22	0.55792
**LRRN1**	leucine rich repeat neuronal 1	-2.82	0.03339	1.32	0.53025
**STAMBPL1**	STAM binding protein-like 1	-2.82	4.00E-05	-1.06	0.70309

**SLFN5**	schlafen family member 5	-2.82	0.01198	-1.44	0.30695
**EIF2AK3**	eukaryotic translation initiation factor 2-alpha kinase 3	-2.81	0.03033	-1.48	0.36778
**GOLT1B**	golgi transport 1B	-2.81	0.00162	-1.67	0.06248
**KIF4A**	kinesin family member 4A	-2.81	0.03618	-1.31	0.54604
**C17orf96**	chromosome 17 open reading frame 96	-2.81	0.00092	-1.08	0.75009
**MCL1**	myeloid cell leukemia sequence 1 (BCL2-related)	-2.81	0.04619	-1.34	0.53341
**BUB1**	budding uninhibited by benzimidazoles 1 homolog (yeast)	-2.81	0.00396	-1.55	0.14757
**BICD1**	bicaudal D homolog 1 (Drosophila)	-2.80	0.0018	1.01	0.96719
**CENPA**	centromere protein A	-2.80	0.00658	-1.65	0.12915
**EIF5A2**	eukaryotic translation initiation factor 5A2	-2.80	0.00101	1.26	0.32932
**TRIP13**	thyroid hormone receptor interactor 13	-2.80	0.00984	1.09	0.79559
**LINC00263**	long intergenic non-protein coding RNA 263	-2.79	0.00925	-1.05	0.88683
**ESCO2**	establishment of cohesion 1 homolog 2 (S. cerevisiae)	-2.79	0.04139	-1.27	0.59484
**N67305**	---	-2.79	0.00457	-1.02	0.95905
**ULBP2**	UL16 binding protein 2	-2.79	0.00095	-1.61	0.05918
**ESPL1**	extra spindle pole bodies homolog 1 (S. cerevisiae)	-2.79	0.03505	-1.46	0.38809
**BMP8B**	bone morphogenetic protein 8b	-2.79	7.00E-05	-1.47	0.03652
**BID**	BH3 interacting domain death agonist	-2.79	0.03148	1.21	0.65728
**TTC39B**	Tetratricopeptide repeat domain 39B	-2.78	0.00037	-1.27	0.24713
**WFDC2**	WAP four-disulfide core domain 2	-2.78	0.00024	-1.27	0.23415
**RRP7A**	ribosomal RNA processing 7 homolog A (S. cerevisiae)	-2.78	0.02116	-1.43	0.35958
**PFKFB4**	6-phosphofructo-2-kinase/fructose-2,6-biphosphatase 4	-2.78	0.00181	1.2	0.4645
**CYP4F11**	cytochrome P450, family 4, subfamily F, polypeptide 11	-2.77	0.00178	1.01	0.95431
**SHCBP1**	SHC SH2-domain binding protein 1	-2.77	0.03592	-1.44	0.40584
**FADS1**	fatty acid desaturase 1	-2.77	0.00127	1.09	0.72362
**VEGFA**	vascular endothelial growth factor A	-2.77	0.00165	-1.12	0.64269
**C9orf72**	chromosome 9 open reading frame 72	-2.77	0.00165	-1.01	0.96459
**NUSAP1**	nucleolar and spindle associated protein 1	-2.77	0.00225	-1.47	0.15907
**LOC440288**	uncharacterized LOC440288	-2.76	0.02952	-1.31	0.52302
**CORO2B**	coronin, actin binding protein, 2B	-2.76	0.00062	1.95	0.00943
**SPOPL**	speckle-type POZ protein-like	-2.76	0.0025	-1.46	0.17139
**TMEFF1**	transmembrane protein with EGF-like and two follistatin-like domains 1	-2.76	0.01735	1.1	0.79891
**TMEM231**	transmembrane protein 231	-2.76	0.00393	1.67	0.09057
**PRKD1**	protein kinase D1	-2.75	0.00064	1.15	0.51782
**UPP1**	uridine phosphorylase 1	-2.75	0.0041	-1.1	0.74882
**PPARG**	peroxisome proliferator-activated receptor gamma	-2.75	9.00E-04	-1.46	0.11478
**UBTD2**	ubiquitin domain containing 2	-2.75	1.00E-05	-1.56	0.00367
**FHOD1**	formin homology 2 domain containing 1	-2.75	8.00E-04	-1.09	0.69104
**IL20RB**	interleukin 20 receptor beta	-2.75	0.03429	-1.32	0.52005
**FGFR4**	fibroblast growth factor receptor 4	-2.74	0.0211	-1.38	0.40818
**SUB1**	SUB1 homolog (S. cerevisiae)	-2.74	4.00E-05	-1.78	0.00313
**MYC**	v-myc myelocytomatosis viral oncogene homolog (avian)	-2.74	1.00E-05	-1.23	0.11935
**RCAN1**	regulator of calcineurin 1	-2.74	5.00E-05	-1.52	0.01944
**FBXO5**	F-box protein 5	-2.74	0.03521	1.06	0.89174

**SPRY1**	sprouty homolog 1, antagonist of FGF signaling (Drosophila)	-2.73	0.00472	-1.08	0.78259
**GRK5**	G protein-coupled receptor kinase 5	-2.73	0.00112	-1.04	0.85224
**EGLN3**	egl nine homolog 3 (C. elegans)	-2.73	0.01171	-1.53	0.22465
**PADI3**	peptidyl arginine deiminase, type III	-2.73	2.00E-05	-1.01	0.92524
**FANCI**	Fanconi anemia, complementation group I	-2.72	0.00417	1.17	0.57839
**COL21A1**	collagen, type XXI, alpha 1	-2.72	0.00061	1.14	0.52966
**RBP4**	Retinol binding protein 4, plasma	-2.72	0.00435	-1.61	0.11396
**TSPAN18**	tetraspanin 18	-2.72	0.00015	1.03	0.86381
**BARD1**	BRCA1 associated RING domain 1	-2.72	0.00688	1.03	0.91666
**CLIP2**	CAP-GLY domain containing linker protein 2	-2.72	0.0431	-1.24	0.63537
**BE271180**	---	-2.72	0.01322	-1.34	0.40306
**ADA**	adenosine deaminase	-2.72	0.00077	-1.09	0.7056
**CXCL16**	chemokine (C-X-C motif) ligand 16	-2.71	0.00303	-1.89	0.0338
**FAM63B**	family with sequence similarity 63, member B	-2.71	0.03303	-2.02	0.11366
**LOC100506895**	uncharacterized LOC100506895	-2.71	0.00604	-1.12	0.69735
**CHST2**	carbohydrate (N-acetylglucosamine-6-O) sulfotransferase 2	-2.71	0.0394	-2.14	0.10182
**B4GALNT1**	beta-1,4-N-acetyl-galactosaminyl transferase 1	-2.71	0.03174	-1.17	0.71048
**NRN1**	neuritin 1	-2.70	0.02391	1.1	0.79767
**TMPRSS4**	transmembrane protease, serine 4	-2.70	0.00069	1.3	0.24116
**KIAA1324**	KIAA1324	-2.70	0.00626	-1.18	0.58551
**NCR3LG1**	natural killer cell cytotoxicity receptor 3 ligand 1	-2.70	0.01343	-1.23	0.54867
**SH2D5**	SH2 domain containing 5	-2.70	0.00181	1.07	0.78286
**CENPK**	centromere protein K	-2.70	0.02516	1.42	0.37636
**SLC25A43**	solute carrier family 25, member 43	-2.69	0.04933	-2.29	0.09123
**MCM2**	minichromosome maintenance complex component 2	-2.69	0.02566	-1.06	0.87843
**DCLRE1B**	DNA cross-link repair 1B	-2.69	0.00582	1.04	0.90083
**BOP1**	block of proliferation 1	-2.69	0.02716	-1.64	0.22988
**ZNF114**	zinc finger protein 114	-2.69	0.00222	1.15	0.58621
**AW961576**	---	-2.69	4.00E-05	-1.28	0.11532
**LOC100505668**	uncharacterized LOC100505668	-2.69	0.00067	1.1	0.6434
**RNF144A**	ring finger protein 144A	-2.69	0.00058	-1	0.98744
**NCAPG**	non-SMC condensin I complex, subunit G	-2.69	0.00193	-1.41	0.18312
**IL7R**	interleukin 7 receptor	-2.69	0.00402	1.38	0.26046
**LINC00313**	long intergenic non-protein coding RNA 313	-2.69	0.00567	-1.16	0.61782
**AI361034**	---	-2.69	0.00458	-1.96	0.03328
**AURKA**	aurora kinase A	-2.68	0.00332	-1.47	0.16832
**LARP6**	La ribonucleoprotein domain family, member 6	-2.68	0.00864	-1.15	0.66568
**ORC6**	origin recognition complex, subunit 6	-2.68	0.00107	-1.11	0.65157
**MAK16**	MAK16 homolog (S. cerevisiae)	-2.67	3.00E-04	-1.26	0.24297
**BRI3BP**	BRI3 binding protein	-2.67	0.00234	-1.59	0.08937
**AI479285**	---	-2.67	0.00614	-1.09	0.7642
**DSCC1**	defective in sister chromatid cohesion 1 homolog (S. cerevisiae)	-2.67	0.00655	1.43	0.24405
**TTC25**	tetratricopeptide repeat domain 25	-2.67	0.00066	-1.22	0.35888
**PTRH2**	peptidyl-tRNA hydrolase 2	-2.67	3.00E-05	-1.88	0.00112
**FAM102B**	family with sequence similarity 102, member B	-2.67	0.03744	-1.12	0.7825
**C12orf52**	chromosome 12 open reading frame 52	-2.67	0.01981	-1.98	0.08286
**KNTC1**	kinetochore associated 1	-2.67	0.0011	1.16	0.51727
**AL117546**	---	-2.67	0.00285	-1.77	0.04714
**KCTD15**	potassium channel tetramerisation domain containing 15	-2.67	0.00125	1.1	0.68588

**ALAD**	aminolevulinate dehydratase	-2.66	0.02093	-1.77	0.14421
**POLA2**	polymerase (DNA directed), alpha 2, accessory subunit	-2.66	0.02087	-1.18	0.65931
**PAPPA**	pregnancy-associated plasma protein A, pappalysin 1	-2.66	0.01142	1.15	0.66179
**PRKAG2-AS1**	PRKAG2 antisense RNA 1 (non-protein coding)	-2.66	0.02381	1.18	0.65786
**POLE3**	polymerase (DNA directed), epsilon 3, accessory subunit	-2.66	0.046	-1.16	0.73987
**CCDC64**	coiled-coil domain containing 64	-2.66	0.00017	1.31	0.14993
**SERTAD4**	SERTA domain containing 4	-2.66	0.00373	1.09	0.75581
**GK**	glycerol kinase	-2.65	0.00082	-1.15	0.51226
**OAF**	OAF homolog (Drosophila)	-2.65	0.00922	-1.35	0.34344
**AI638195**	---	-2.65	0.00427	-1.17	0.56295
**ETV4**	ets variant 4	-2.65	5.00E-05	-1.07	0.67106
**MCTP2**	multiple C2 domains, transmembrane 2	-2.65	0.01823	1.46	0.30328
**CENPI**	centromere protein I	-2.64	0.01343	1.01	0.98709
**BLM**	Bloom syndrome, RecQ helicase-like	-2.64	0.00072	-1.01	0.95799
**TM4SF1**	transmembrane 4 L six family member 1	-2.64	0.00084	-1.12	0.59262
**ITGAX**	integrin, alpha X (complement component 3 receptor 4 subunit)	-2.64	7.00E-05	-1.07	0.68765
**TDP1**	tyrosyl-DNA phosphodiesterase 1	-2.64	0.02616	-1.29	0.51841
**CNN3**	calponin 3, acidic	-2.64	0.01294	-1.01	0.96797
**KLHL23**	kelch-like 23 (Drosophila)	-2.63	0.00103	-1.3	0.24451
**FOXE1**	forkhead box E1 (thyroid transcription factor 2)	-2.63	0.00761	-1.25	0.4617
**PIGH**	phosphatidylinositol glycan anchor biosynthesis, class H	-2.63	0.00153	-1.93	0.01558
**GPR126**	G protein-coupled receptor 126	-2.63	2.00E-05	1.08	0.56505
**DNAL1**	dynein, axonemal, light chain 1	-2.63	0.02379	-1.14	0.72023
**PLK4**	polo-like kinase 4	-2.63	0.02073	1.13	0.73025
**BIRC3**	baculoviral IAP repeat containing 3	-2.62	0.01083	1.18	0.60078
**SLC39A14**	solute carrier family 39 (zinc transporter), member 14	-2.62	0.00018	-1.7	0.01055
**EPDR1**	ependymin related protein 1 (zebrafish)	-2.62	0.00033	-1.06	0.75784
**FOXN2**	forkhead box N2	-2.62	0	-1.41	0.00385
**W91876**	---	-2.62	0.00221	1.18	0.50045
**LOC100507672**	uncharacterized LOC100507672	-2.62	0.00026	-1.17	0.40999
**STK17B**	serine/threonine kinase 17b	-2.62	0.00872	-1.37	0.3112
**EIF3J**	eukaryotic translation initiation factor 3, subunit J	-2.62	0.00417	-1.85	0.04021
**FEN1**	flap structure-specific endonuclease 1	-2.61	0.01326	1.02	0.95445
**AI686793**	---	-2.61	0.00132	-1.1	0.68277
**OVOS2**	ovostatin 2	-2.61	0.00123	1.01	0.95877
**LONRF3**	LON peptidase N-terminal domain and ring finger 3	-2.61	0.00106	-1.3	0.24914
**RQCD1**	RCD1 required for cell differentiation1 homolog (S. pombe)	-2.61	0.01114	-1.58	0.17275
**PCED1B**	PC-esterase domain containing 1B	-2.61	0.00039	-1.18	0.39518
**CRIM1**	cysteine rich transmembrane BMP regulator 1 (chordin-like)	-2.60	0.00744	-1.18	0.57718
**KCTD12**	potassium channel tetramerisation domain containing 12	-2.60	0.00978	-1.34	0.36295
**THAP10**	THAP domain containing 10	-2.60	0.00341	1.14	0.6232
**MNS1**	meiosis-specific nuclear structural 1	-2.60	0.00474	1.24	0.44304
**CLSPN**	claspin	-2.60	0.02098	1.54	0.24794
**AL110176**	---	-2.60	0.00086	-1.57	0.05285

**TCF3**	transcription factor 3 (E2A immunoglobulin enhancer binding factors E12/E47)	-2.60	0.00249	-1.75	0.04176
**METTL21A**	methyltransferase like 21A	-2.60	0.00919	-1.3	0.39699
**GYG2**	glycogenin 2	-2.60	0.00267	-1.16	0.56191
**UHRF1**	ubiquitin-like with PHD and ring finger domains 1	-2.60	0.00694	1.1	0.74569
**H59221**	---	-2.60	0.00209	-1.12	0.64994
**AW274560**	---	-2.59	0.00052	1.09	0.67123
**C11orf75**	chromosome 11 open reading frame 75	-2.59	3.00E-05	-1.06	0.69045
**LOC439949**	uncharacterized LOC439949	-2.59	0.00097	1.13	0.58207
**SYBU**	syntabulin (syntaxin-interacting)	-2.59	0.03692	1.08	0.85227
**MOXD1**	monooxygenase, DBH-like 1	-2.59	3.00E-04	-1.04	0.82282
**VMA21**	VMA21 vacuolar H+-ATPase homolog (S. cerevisiae)	-2.58	0.00014	-1.71	0.0077
**LOC401052**	uncharacterized LOC401052	-2.58	0.01759	1.1	0.77821
**ETS2**	V-ets erythroblastosis virus E26 oncogene homolog 2 (avian)	-2.58	0.00025	-1.02	0.91849
**UBL3**	ubiquitin-like 3	-2.58	0.01858	-2.09	0.05524
**GRB10**	growth factor receptor-bound protein 10	-2.58	0.0048	-1.5	0.15747
**AA133285**	---	-2.58	0.01514	-1.1	0.78522
**SEL1L**	sel-1 suppressor of lin-12-like (C. elegans)	-2.58	0.00085	-1.97	0.00758
**BRCA1**	breast cancer 1, early onset	-2.58	0.00862	1	0.98965
**LRR1**	leucine rich repeat protein 1	-2.58	0.04335	1.07	0.87634
**FABP4**	fatty acid binding protein 4, adipocyte	-2.57	0.02591	1.05	0.89379
**AA404273**	---	-2.57	0.02283	-1.6	0.21323
**C12orf75**	chromosome 12 open reading frame 75	-2.57	0.0174	1.37	0.36353
**LOC284561**	uncharacterized LOC284561	-2.57	0.01754	-1.15	0.6847
**AI475033**	---	-2.57	0.00162	-1.08	0.74524
**MAD2L1**	MAD2 mitotic arrest deficient-like 1 (yeast)	-2.57	0.03534	-1.02	0.95535
**MYO10**	myosin X	-2.57	0.00014	-1.16	0.37092
**THBS1**	thrombospondin 1	-2.57	0.00079	-1.15	0.49431
**EOMES**	eomesodermin	-2.57	0.00381	-1.09	0.73514
**HEY2**	hairy/enhancer-of-split related with YRPW motif 2	-2.56	0.00556	1.13	0.65773
**GALNTL4**	UDP-N-acetyl-alpha-D-galactosamine:polypeptide N-acetylgalactosaminyltransferase-like 4	-2.56	0.00923	1.06	0.85443
**EAF2**	ELL associated factor 2	-2.56	0.00149	-1.24	0.35027
**HHEX**	hematopoietically expressed homeobox	-2.56	0.04495	1.18	0.70322
**MLF1IP**	MLF1 interacting protein	-2.55	0.00105	1.44	0.10925
**ARRB2**	arrestin, beta 2	-2.55	0.04086	-1.29	0.54157
**ADAM8**	ADAM metallopeptidase domain 8	-2.55	0.00074	1.08	0.72133
**TACC3**	transforming, acidic coiled-coil containing protein 3	-2.55	0.01022	-1.37	0.31784
**BE502264**	---	-2.55	1.00E-04	-1.1	0.55361
**AW151660**	---	-2.55	7.00E-05	-1.62	0.00828
**ZWINT**	ZW10 interactor	-2.55	0.01679	1.04	0.89744
**STRBP**	spermatid perinuclear RNA binding protein	-2.55	0.01308	-1.36	0.34762
**CCDC90A**	coiled-coil domain containing 90A	-2.55	0.02223	-1.12	0.75685
**SGOL2**	shugoshin-like 2 (S. pombe)	-2.55	0.0046	-1.28	0.36083
**FNDC3A**	fibronectin type III domain containing 3A	-2.55	0.01236	-1.31	0.40733
**POLR3G**	polymerase (RNA) III (DNA directed) polypeptide G (32kD)	-2.54	0.01263	1.37	0.32849
**TMTC2**	transmembrane and tetratricopeptide repeat containing 2	-2.54	0.00252	-1.37	0.20843
**GAS2L3**	growth arrest-specific 2 like 3	-2.54	0.04244	-1.1	0.81815
**SMC4**	structural maintenance of chromosomes 4	-2.54	0.00483	-1.4	0.22406

**VMP1**	vacuole membrane protein 1	-2.54	9.00E-05	-1.39	0.05417
**AI300077**	---	-2.54	4.00E-05	1.41	0.02884
**NNMT**	nicotinamide N-methyltransferase	-2.53	0.005	-1.42	0.20975
**RAD54L**	RAD54-like (S. cerevisiae)	-2.53	0.01111	1.44	0.25023
**PCSK5**	proprotein convertase subtilisin/kexin type 5	-2.53	0.00853	-1.13	0.67265
**ARNTL**	aryl hydrocarbon receptor nuclear translocator-like	-2.53	0.00208	-1.1	0.6711
**SEMA6D**	sema domain, transmembrane domain (TM), and cytoplasmic domain, (semaphorin) 6D	-2.53	0.03247	1.25	0.56394
**LOC100505730**	uncharacterized LOC100505730	-2.52	0.00012	1.32	0.10401
**BF115851**	---	-2.52	0.00028	-1.31	0.14364
**LZTFL1**	leucine zipper transcription factor-like 1	-2.52	0.00664	-1.5	0.17059
**SH2D3A**	SH2 domain containing 3A	-2.52	0.01852	-1.22	0.56017
**PRR3**	proline rich 3	-2.52	0.00145	-1.4	0.1508
**PTX3**	pentraxin 3, long	-2.52	0.02566	1.41	0.35397
**RANBP1**	RAN binding protein 1	-2.51	0.00084	-1.21	0.3568
**E2F2**	E2F transcription factor 2	-2.51	0.04144	1.29	0.53078
**TMEM209**	transmembrane protein 209	-2.51	0.00339	-1.13	0.61827
**HS6ST2**	heparan sulfate 6-O-sulfotransferase 2	-2.51	7.00E-05	-1.02	0.89493
**KIF2C**	kinesin family member 2C	-2.51	0.0114	-1.32	0.38124
**FKBP11**	FK506 binding protein 11, 19 kDa	-2.51	0.00359	1.04	0.88556
**SYTL2**	synaptotagmin-like 2	-2.51	0.00117	-1.5	0.07694
**HTATSF1P2 ***	HIV-1 Tat specific factor 1 pseudogene 2	-2.51	0.00041	2.03	0.00266
**FOXM1**	forkhead box M1	-2.51	0.02301	-1.47	0.28535
**LINC00621**	long intergenic non-protein coding RNA 621	-2.50	0.00713	-1.56	0.13372
**FHDC1**	FH2 domain containing 1	-2.50	0.01311	1.48	0.22749
**ARL10**	ADP-ribosylation factor-like 10	-2.50	0.00162	1.08	0.71664
**PNP**	purine nucleoside phosphorylase	-2.49	0.00677	-1	0.99832
**PANX1**	pannexin 1	-2.49	0.00847	1.11	0.7183
**ATP2C2**	ATPase, Ca++ transporting, type 2C, member 2	-2.49	0.01694	-1.41	0.30556
**SREBF1**	sterol regulatory element binding transcription factor 1	-2.49	0.00164	-1.49	0.09318
**ANP32E**	acidic (leucine-rich) nuclear phosphoprotein 32 family, member E	-2.49	0.00624	1.01	0.98354
**SMAD2**	SMAD family member 2	-2.49	3.00E-05	-1.89	0.00058
**HMGA1**	high mobility group AT-hook 1	-2.48	0.00011	-1.3	0.10833
**DCUN1D4**	DCN1, defective in cullin neddylation 1, domain containing 4 (S. cerevisiae)	-2.48	0.00594	1.24	0.43264
**PASK**	PAS domain containing serine/threonine kinase	-2.48	0.04558	1.54	0.30582
**SPATA13**	spermatogenesis associated 13	-2.48	0.03993	-1.17	0.69167
**CDKL1**	cyclin-dependent kinase-like 1 (CDC2-related kinase)	-2.48	0.0024	1.19	0.45233
**ELOVL5**	ELOVL fatty acid elongase 5	-2.48	0.00051	-1.23	0.2896
**PYCR1**	pyrroline-5-carboxylate reductase 1	-2.48	0.00853	-1.39	0.26417
**COBL**	cordon-bleu homolog (mouse)	-2.48	6.00E-05	-1.21	0.19826
**CPNE8**	copine VIII	-2.48	0.00015	-1.15	0.39584
**IQGAP3**	IQ motif containing GTPase activating protein 3	-2.48	0.03527	-1.25	0.56398
**FAM40B**	family with sequence similarity 40, member B	-2.47	0.01704	1.69	0.12949
**AA142959**	---	-2.47	0.01508	1.7	0.11851
**KCTD20**	potassium channel tetramerisation domain containing 20	-2.47	0.00021	-1.67	0.00965
**TYRP1**	tyrosinase-related protein 1	-2.47	0.03317	-1.08	0.83029
**GEMIN5**	gem (nuclear organelle) associated protein 5	-2.47	0.00879	-1.02	0.94158
**LOC283050**	uncharacterized LOC283050	-2.47	0.00096	1.29	0.22704

**AA933082**	---	-2.46	0.01178	1.21	0.53384
**RNASEH2A**	ribonuclease H2, subunit A	-2.46	0.01731	1.34	0.38308
**TYMS**	thymidylate synthetase	-2.46	0.00819	1.18	0.56989
**MMP28**	matrix metallopeptidase 28	-2.46	0.02048	1	0.99977
**STK17A**	serine/threonine kinase 17a	-2.46	0.00219	-1.21	0.41295
**FBLN1**	fibulin 1	-2.46	0.00057	-1.2	0.33659
**RNASEH1**	ribonuclease H1	-2.46	0.00052	-1.81	0.00839
**LHX2**	LIM homeobox 2	-2.45	0.03229	1.1	0.80041
**SLC1A5**	solute carrier family 1 (neutral amino acid transporter), member 5	-2.45	0.00133	-1.02	0.94146
**MID1IP1**	MID1 interacting protein 1	-2.45	0.00017	1.01	0.94805
**ITPR1**	inositol 1,4,5-trisphosphate receptor, type 1	-2.45	0.00145	-1.01	0.97876
**MYBL1**	v-myb myeloblastosis viral oncogene homolog (avian)-like 1	-2.45	0.00726	1.54	0.14056
**UBE2S**	ubiquitin-conjugating enzyme E2S	-2.45	0.00311	-1.71	0.04491
**MAP3K5**	mitogen-activated protein kinase kinase kinase 5	-2.45	0.00771	1.21	0.50078
**F2R**	coagulation factor II (thrombin) receptor	-2.45	0.00287	1.15	0.57001
**NPR3**	natriuretic peptide receptor C/guanylate cyclase C (atrionatriuretic peptide receptor C)	-2.45	0.02913	-1.08	0.82628
**OIP5**	Opa interacting protein 5	-2.45	0.02091	1.06	0.86146
**AA416756**	---	-2.44	0.00676	-1.18	0.54798
**HPRT1**	hypoxanthine phosphoribosyltransferase 1	-2.44	0.00084	1.28	0.22244
**C19orf48**	chromosome 19 open reading frame 48	-2.44	0.00313	-1.33	0.24641
**MIR21**	microRNA 21	-2.44	0.00043	-1.62	0.02052
**AW954199**	---	-2.44	0.00038	-1.53	0.03423
**CLMN**	calmin (calponin-like, transmembrane)	-2.44	3.00E-05	1.49	0.00931
**FADS2**	fatty acid desaturase 2	-2.44	0.04319	1.21	0.62658
**CCDC71L**	coiled-coil domain containing 71-like	-2.44	0.00201	1.63	0.04777
**KIAA0040**	KIAA0040	-2.44	0.00094	1.28	0.23759
**MICU1**	mitochondrial calcium uptake 1	-2.44	4.00E-04	-1.84	0.00522
**BACH2**	BTB and CNC homology 1, basic leucine zipper transcription factor 2	-2.44	0.00147	1.01	0.97045
**TROAP**	trophinin associated protein (tastin)	-2.43	0.03039	-1.81	0.12464
**AI247368**	---	-2.43	0.03106	-1.33	0.44296
**PID1**	phosphotyrosine interaction domain containing 1	-2.43	0.00313	1.28	0.31848
**WRAP53**	WD repeat containing, antisense to TP53	-2.43	0.01505	-1.78	0.088
**KIF11**	kinesin family member 11	-2.43	0.01628	-1.08	0.80488
**AI986112**	---	-2.43	0.00623	-1.34	0.28044
**ATP11A**	ATPase, class VI, type 11A	-2.43	0.00037	-1.51	0.0372
**KRT80**	keratin 80	-2.43	0.0079	1.08	0.79106
**AI797678**	---	-2.43	0.0016	-1.02	0.91491
**PRC1**	protein regulator of cytokinesis 1	-2.43	0.02746	1.01	0.97664
**NUS1**	nuclear undecaprenyl pyrophosphate synthase 1 homolog (S. cerevisiae)	-2.43	0.02409	-1.44	0.30188
**NMU**	neuromedin U	-2.42	0.00326	-1.72	0.0412
**PLXNA2**	plexin A2	-2.42	0.00274	1.35	0.20934
**VWA1**	Von Willebrand factor A domain containing 1	-2.42	0.01244	1.04	0.88984
**TSPAN4**	tetraspanin 4	-2.42	0.02476	-1.5	0.2583
**AGPAT5**	1-acylglycerol-3-phosphate O-acyltransferase 5 (lysophosphatidic acid acyltransferase, epsilon)	-2.42	0.02888	-1.3	0.46346
**CCDC88A**	coiled-coil domain containing 88A	-2.42	0.01121	-1.31	0.36406
**ARF3**	ADP-ribosylation factor 3	-2.42	0.02157	-1.16	0.65946
**LRRC49**	leucine rich repeat containing 49	-2.42	0.00481	-1.29	0.33232
**APOLD1**	apolipoprotein L domain containing 1	-2.42	0.01385	-1.03	0.92491

**AU144916**	---	-2.42	0.0269	1.24	0.53798
**C14orf182**	chromosome 14 open reading frame 182	-2.42	0.00226	-1.17	0.48849
**LANCL2**	LanC lantibiotic synthetase component C-like 2 (bacterial)	-2.42	0.00485	-1.85	0.03153
**NETO2**	neuropilin (NRP) and tolloid (TLL)-like 2	-2.41	0.00187	1.07	0.7651
**KIF18A**	kinesin family member 18A	-2.41	0.0258	-1.69	0.15318
**TAP2**	transporter 2, ATP-binding cassette, sub-family B (MDR/TAP)	-2.41	0.01963	1.34	0.37559
**AW612461**	---	-2.41	1.00E-04	-1.11	0.50334
**PLXND1**	plexin D1	-2.41	0.00075	1.13	0.52631
**AW293012**	---	-2.41	0.00121	-1.48	0.07487
**RNF24**	ring finger protein 24	-2.40	0.00043	-1.09	0.62716
**CORO2A**	coronin, actin binding protein, 2A	-2.40	0.03249	1.15	0.69604
**TMEM154**	transmembrane protein 154	-2.40	0.00652	-1.27	0.37194
**ARNT2**	aryl-hydrocarbon receptor nuclear translocator 2	-2.40	0.00266	-1.29	0.28569
**POLR3B**	polymerase (RNA) III (DNA directed) polypeptide B	-2.40	0.01732	-1.69	0.12104
**CD44**	CD44 molecule (Indian blood group)	-2.40	0.00231	-1.73	0.03049
**NOLC1**	nucleolar and coiled-body phosphoprotein 1	-2.39	0.00096	-1.28	0.22562
**UCK2**	uridine-cytidine kinase 2	-2.39	0.00064	-1.05	0.77872
**TFDP1**	transcription factor Dp-1	-2.39	0.04555	-1.36	0.43873
**TCOF1**	Treacher Collins-Franceschetti syndrome 1	-2.39	4.00E-05	-1.3	0.0639
**ADAM28**	ADAM metallopeptidase domain 28	-2.39	0.00022	1.1	0.55921
**USP14**	ubiquitin specific peptidase 14 (tRNA-guanine transglycosylase)	-2.39	0	-1.94	4.00E-05
**TNFAIP8**	tumor necrosis factor, alpha-induced protein 8	-2.39	0.00443	-1.29	0.31994
**TMED4 ***	transmembrane emp24 protein transport domain containing 4	-2.39	0.00333	2.73	0
**IPO5**	importin 5	-2.39	0.00232	-1.43	0.1274
**RASIP1**	Ras interacting protein 1	-2.39	0.00269	1.12	0.61314
**PYCR2**	pyrroline-5-carboxylate reductase family, member 2	-2.39	0.02661	-1.04	0.91637
**GLT25D1**	glycosyltransferase 25 domain containing 1	-2.38	0.02264	-1.52	0.22275
**C5orf34**	chromosome 5 open reading frame 34	-2.38	0.00023	1.03	0.87192
**PDE3B**	phosphodiesterase 3B, cGMP-inhibited	-2.38	0.02226	-1.5	0.23952
**ATP8A1**	ATPase, aminophospholipid transporter (APLT), class I, type 8A, member 1	-2.38	0.02088	-1.45	0.27194
**ZAK**	sterile alpha motif and leucine zipper containing kinase AZK	-2.38	2.00E-05	-1.6	0.00221
**POC1A**	POC1 centriolar protein homolog A (Chlamydomonas)	-2.38	0.02784	-1.72	0.13972
**MTG1**	Mitochondrial GTPase 1 homolog (S. cerevisiae)	-2.38	0.02017	-1.97	0.05623
**CBX2**	chromobox homolog 2	-2.38	0.01245	1.27	0.4204
**AI200546**	---	-2.38	0.04207	-1.66	0.20524
**CENPH**	centromere protein H	-2.38	9.00E-04	1.08	0.7069
**XBP1**	X-box binding protein 1	-2.38	0.00331	-1.43	0.15053
**DKC1**	dyskeratosis congenita 1, dyskerin	-2.37	0.00029	1.01	0.96711
**ZNF367**	zinc finger protein 367	-2.37	0.03245	2.01	0.0731
**GNLY**	granulysin	-2.37	0.00021	1.06	0.72621
**DDX39A**	DEAD (Asp-Glu-Ala-Asp) box polypeptide 39A	-2.37	0.01105	-1.27	0.41319
**PER1**	period homolog 1 (Drosophila)	-2.37	0.02402	-1.24	0.52737
**NASP**	nuclear autoantigenic sperm protein (histone-binding)	-2.37	0.00449	1.21	0.43475
**BG231709**	---	-2.37	0.00197	-1.64	0.03915

**POLR2D**	polymerase (RNA) II (DNA directed) polypeptide D	-2.37	0.00036	-1.64	0.01309
**CACNA1D**	calcium channel, voltage-dependent, L type, alpha 1D subunit	-2.37	0.03258	1.52	0.25503
**PAG1**	phosphoprotein associated with glycosphingolipid microdomains 1	-2.36	0.01839	1.66	0.12838
**RAB43**	RAB43, member RAS oncogene family	-2.36	0.04006	-1.49	0.29885
**LOC728431**	uncharacterized LOC728431	-2.36	0.01613	-1	0.99482
**TMEM170B**	transmembrane protein 170B	-2.36	0.01954	-1.18	0.61547
**CDADC1**	cytidine and dCMP deaminase domain containing 1	-2.36	0.02164	-1.31	0.41904
**PPP3CB**	protein phosphatase 3, catalytic subunit, beta isozyme	-2.36	0.00086	-1.69	0.01756
**LAMB1**	laminin, beta 1	-2.36	6.00E-05	1.2	0.19315
**C11orf96**	chromosome 11 open reading frame 96	-2.36	0.00313	-1.28	0.29579
**LIPG**	lipase, endothelial	-2.36	0.02665	-1.53	0.22736
**WWTR1-AS1**	WWTR1 antisense RNA 1 (non-protein coding)	-2.36	0.03256	-1.14	0.72012
**HILPDA**	hypoxia inducible lipid droplet-associated	-2.35	0.00459	-1.22	0.42727
**MCTP1**	multiple C2 domains, transmembrane 1	-2.35	0.00031	1.57	0.01843
**PLEKHA6**	pleckstrin homology domain containing, family A member 6	-2.34	0.00899	1.23	0.4595
**KCNS3**	potassium voltage-gated channel, delayed-rectifier, subfamily S, member 3	-2.34	0.0021	1.19	0.42827
**ZFP64**	Zinc finger protein 64 homolog (mouse)	-2.34	0.00925	-1.05	0.85819
**PDE8B**	phosphodiesterase 8B	-2.34	0.02764	-1.31	0.43286
**NEK2**	NIMA (never in mitosis gene a)-related kinase 2	-2.34	0.00354	-1.21	0.42225
**NPNT**	nephronectin	-2.34	0.01275	-1.28	0.40209
**RECQL**	RecQ protein-like (DNA helicase Q1-like)	-2.34	0.01012	-1.33	0.31911
**MAGOHB**	mago-nashi homolog B (Drosophila)	-2.34	0.03775	1.13	0.73338
**KCNC4**	potassium voltage-gated channel, Shaw-related subfamily, member 4	-2.34	0.01957	1.04	0.90332
**GTSE1**	G-2 and S-phase expressed 1	-2.34	0.04456	-1.18	0.66029
**TXNDC5**	thioredoxin domain containing 5 (endoplasmic reticulum)	-2.34	2.00E-05	-1.23	0.11478
**CMTM3**	CKLF-like MARVEL transmembrane domain containing 3	-2.33	5.00E-05	-1.11	0.4268
**PTGES**	prostaglandin E synthase	-2.33	0.00273	1.06	0.80894
**TAF1A**	TATA box binding protein (TBP)-associated factor, RNA polymerase I, A, 48kDa	-2.33	0.01708	-1.02	0.93794
**NRIP1**	nuclear receptor interacting protein 1	-2.33	0.00093	-1.62	0.02643
**LOC284751**	uncharacterized LOC284751	-2.33	0.0069	1.09	0.73639
**NCAPH2**	non-SMC condensin II complex, subunit H2	-2.33	0.01759	-1.11	0.74224
**PPCDC**	phosphopantothenoylcysteine decarboxylase	-2.33	0.04319	-1.69	0.18142
**CELF2**	CUGBP, Elav-like family member 2	-2.33	0.01169	1.34	0.31535
**PPP2R2A**	protein phosphatase 2, regulatory subunit B, alpha	-2.33	0.00149	-1.82	0.0121
**NCALD**	neurocalcin delta	-2.33	0.00014	-1.15	0.36343
**SIPA1L1**	signal-induced proliferation-associated 1 like 1	-2.33	0.00601	1.2	0.46585
**RFC4**	replication factor C (activator 1) 4, 37kDa	-2.32	0.00145	1.22	0.33972
**PRR7-AS1**	PRR7 antisense RNA 1 (non-protein coding)	-2.32	0.00051	-1.3	0.15582
**MDC1**	mediator of DNA-damage checkpoint 1	-2.32	0.00971	-1.69	0.07614
**BC000885**	---	-2.32	0.03295	-1.3	0.46512
**MIR3682**	microRNA 3682	-2.32	0.02887	-1.88	0.08475
**DISC1**	disrupted in schizophrenia 1	-2.32	0.03095	-1.76	0.12541
**BFSP1**	beaded filament structural protein 1, filensin	-2.32	0.00851	-1.26	0.39768

**FAM105A ***	family with sequence similarity 105, member A	-2.32	0.01603	2.36	0.01452
**NCOA7**	nuclear receptor coactivator 7	-2.32	0.00039	-1.37	0.08542
**AF052109**	---	-2.32	0.04632	1.08	0.83737
**RASAL1**	RAS protein activator like 1 (GAP1 like)	-2.32	0.00679	1	0.99857
**AV702787**	---	-2.31	0.00058	-1.1	0.57949
**RFC3**	replication factor C (activator 1) 3, 38kDa	-2.31	0.02842	1.36	0.36987
**IL15RA**	interleukin 15 receptor, alpha	-2.31	0.00159	-1.23	0.31591
**TUBA1A**	tubulin, alpha 1a	-2.31	0.04361	1.23	0.57629
**NRTN**	neurturin	-2.31	0.03453	1.27	0.5095
**ROBO3**	roundabout, axon guidance receptor, homolog 3 (Drosophila)	-2.31	0.00022	-1.3	0.11889
**CDC42EP1**	CDC42 effector protein (Rho GTPase binding) 1	-2.31	0.00354	-1.64	0.04973
**TBC1D1**	TBC1 (tre-2/USP6, BUB2, cdc16) domain family, member 1	-2.31	0.00108	1.24	0.2806
**DPYSL2**	dihydropyrimidinase-like 2	-2.31	0.0017	1.32	0.1926
**PIGA**	phosphatidylinositol glycan anchor biosynthesis, class A	-2.31	0.00549	1	0.98415
**TNFRSF10B**	tumor necrosis factor receptor superfamily, member 10b	-2.31	0.02723	-1.33	0.40738
**LOC100129104**	uncharacterized LOC100129104	-2.31	6.00E-05	-1.26	0.10608
**DCTN1**	dynactin 1	-2.31	0.0107	1.23	0.4649
**BCL10**	B-cell CLL/lymphoma 10	-2.31	0.00822	-1.4	0.21756
**PAX6**	paired box 6	-2.31	0.00485	1.03	0.89084
**TRIM27**	tripartite motif containing 27	-2.31	0.00749	-1.34	0.27343
**PRKCA**	protein kinase C, alpha	-2.31	0.00127	-1.71	0.01815
**TRAF3IP3**	TRAF3 interacting protein 3	-2.31	0.00154	-1.31	0.19567
**SGMS2**	sphingomyelin synthase 2	-2.31	2.00E-05	1.07	0.59431
**VARS**	valyl-tRNA synthetase	-2.31	0.00934	-1.23	0.45472
**AL050136**	---	-2.31	0.02903	-1.34	0.39566
**IER5**	immediate early response 5	-2.31	0.0228	-1.23	0.52188
**CCDC147**	coiled-coil domain containing 147	-2.31	0.02621	-1.22	0.5585
**MSANTD2**	Myb/SANT-like DNA-binding domain containing 2	-2.31	0.02649	-1.14	0.70128
**RFESD**	Rieske (Fe-S) domain containing	-2.31	0.00189	-1.2	0.38633
**SNX10**	sorting nexin 10	-2.31	0.0223	1.39	0.31104
**PSEN1**	presenilin 1	-2.31	0.03516	-1.67	0.16472
**MGC39372**	serpin peptidase inhibitor, clade B (ovalbumin), member 9 pseudogene	-2.30	0.0039	-1.17	0.50529
**LOC100652770**	Uncharacterized LOC100652770	-2.30	0.00571	-1.06	0.82361
**EIF3E**	eukaryotic translation initiation factor 3, subunit E	-2.30	7.00E-05	-1.8	0.00112
**GPC4**	glypican 4	-2.30	0.00761	-1.29	0.33632
**ENTPD3**	ectonucleoside triphosphate diphosphohydrolase 3	-2.30	0.00629	-1.14	0.59394
**FAM190B**	family with sequence similarity 190, member B	-2.30	0.00196	-1.1	0.66213
**TMEM92**	transmembrane protein 92	-2.30	0.00223	-1.12	0.58951
**PAICS**	phosphoribosylaminoimidazole carboxylase, phosphoribosylaminoimidazole succinocarboxamide synthetase	-2.30	0.00028	-1	0.98934
**SEMA3B**	sema domain, immunoglobulin domain (Ig), short basic domain, secreted, (semaphorin) 3B	-2.30	0.00081	-1.51	0.04284
**FAM84B**	family with sequence similarity 84, member B	-2.30	0.00118	-1.16	0.45548
**BF995452**	---	-2.30	0.04067	-1.37	0.39493
**ADCY3**	adenylate cyclase 3	-2.29	0.00472	1.5	0.10951
**CHAF1B**	chromatin assembly factor 1, subunit B (p60)	-2.29	0.00059	1.25	0.21498

**FJX1**	four jointed box 1 (Drosophila)	-2.29	0.04942	-1.2	0.64211
**RAP2A**	RAP2A, member of RAS oncogene family	-2.29	0.00376	-1.83	0.02146
**LITAF**	lipopolysaccharide-induced TNF factor	-2.29	0.00026	-1.93	0.00147
**LONRF1**	LON peptidase N-terminal domain and ring finger 1	-2.29	0.00133	-1.36	0.13622
**CTSC**	cathepsin C	-2.29	4.00E-05	-1.26	0.08709
**SLC36A4**	solute carrier family 36 (proton/amino acid symporter), member 4	-2.29	0.01173	1.15	0.62712
**SRR**	Serine racemase	-2.29	0.02903	-1.2	0.58904
**EIF2S1**	eukaryotic translation initiation factor 2, subunit 1 alpha, 35kDa	-2.28	0.00095	-1.6	0.02672
**RCC1**	regulator of chromosome condensation 1	-2.28	0.00153	-1.17	0.43042
**PITPNC1**	phosphatidylinositol transfer protein, cytoplasmic 1	-2.28	0.00025	1.06	0.69483
**NABP1**	nucleic acid binding protein 1	-2.28	0.00709	-1.37	0.22549
**CD83**	CD83 molecule	-2.28	0.00021	-1.01	0.94113
**AV646177**	---	-2.28	0.00343	-1.14	0.57543
**HIVEP3**	human immunodeficiency virus type I enhancer binding protein 3	-2.28	0.01828	-1.09	0.78276
**PC**	pyruvate carboxylase	-2.28	0.00514	-1.28	0.3139
**FAP**	fibroblast activation protein, alpha	-2.28	0.03047	-1.12	0.73734
**ZBTB38**	zinc finger and BTB domain containing 38	-2.28	0.00617	-1.09	0.72987
**THRB**	thyroid hormone receptor, beta	-2.28	0.00074	1.01	0.96019
**ORC1**	origin recognition complex, subunit 1	-2.27	0.04202	-1.01	0.97576
**GCSH**	glycine cleavage system protein H (aminomethyl carrier)	-2.27	0.00012	1.02	0.8895
**MOCOS**	molybdenum cofactor sulfurase	-2.27	0.01098	-1.13	0.65229
**ERGIC1**	endoplasmic reticulum-golgi intermediate compartment (ERGIC) 1	-2.27	0.04547	-1.11	0.7814
**BC037339**	---	-2.27	0.01655	-1.12	0.70323
**FKBP5**	FK506 binding protein 5	-2.27	5.00E-05	1.04	0.76769
**FMNL3**	formin-like 3	-2.27	0.02731	1.06	0.85782
**DPYD**	dihydropyrimidine dehydrogenase	-2.27	0.01544	-1.09	0.77531
**AW301241**	---	-2.27	0.03729	-1.63	0.18327
**OGFR**	opioid growth factor receptor	-2.27	0.00811	-1.93	0.02449
**AGXT2L2**	alanine-glyoxylate aminotransferase 2-like 2	-2.27	0.00335	-1.68	0.03746
**NEDD1**	neural precursor cell expressed, developmentally down-regulated 1	-2.27	0.00303	-1.02	0.93983
**RHEBL1 ***	Ras homolog enriched in brain like 1	-2.27	0.00013	2.24	0.00015
**PTER**	phosphotriesterase related	-2.26	6.00E-05	-1.38	0.02738
**ADD3**	adducin 3 (gamma)	-2.26	0.01916	1.02	0.9494
**A2M**	alpha-2-macroglobulin	-2.26	0.01347	-1.18	0.55515
**FGFR1**	fibroblast growth factor receptor 1	-2.26	0.00545	1.39	0.18379
**SPCS3**	signal peptidase complex subunit 3 homolog (S. cerevisiae)	-2.26	0.02401	-1.63	0.14507
**PKP4**	plakophilin 4	-2.26	0.00177	-1.38	0.12607
**PRIM1**	primase, DNA, polypeptide 1 (49kDa)	-2.26	0.01068	-1.23	0.45306
**MOSPD1**	motile sperm domain containing 1	-2.26	0.04144	-1.28	0.49783
**CYP26B1**	cytochrome P450, family 26, subfamily B, polypeptide 1	-2.26	0.00017	1.43	0.02915
**FIBIN**	fin bud initiation factor homolog (zebrafish)	-2.26	0.04173	1.33	0.43852
**E2F1**	E2F transcription factor 1	-2.25	0.00247	-1.31	0.21302
**RGS10**	Regulator of G-protein signaling 10	-2.25	0.00125	-1.14	0.49111
**LOC100506021**	uncharacterized LOC100506021	-2.25	0.00178	1.06	0.76355

**PROCR**	protein C receptor, endothelial	-2.25	0.0136	-1.25	0.42589
**SH2D4A**	SH2 domain containing 4A	-2.25	0.00016	-1.03	0.86157
**AL563613**	---	-2.25	0.00569	-1.51	0.10853
**DNMT1**	DNA (cytosine-5-)-methyltransferase 1	-2.25	0.00827	-1.07	0.80552
**C8orf33**	chromosome 8 open reading frame 33	-2.25	0.00187	-1.91	0.0075
**AGFG1**	ArfGAP with FG repeats 1	-2.25	0.00335	-1.43	0.1261
**AW302848**	---	-2.25	0.00219	-1.05	0.79831
**SLC1A1**	solute carrier family 1 (neuronal/epithelial high affinity glutamate transporter, system Xag), member 1	-2.25	0.04642	1.73	0.15474
**SAMD9**	sterile alpha motif domain containing 9	-2.25	0.02845	1.21	0.56532
**ME2**	malic enzyme 2, NAD(+)-dependent, mitochondrial	-2.25	0.01237	-1.33	0.31499
**BNIP3L**	BCL2/adenovirus E1B 19kDa interacting protein 3-like	-2.25	0.00379	-1.26	0.31986
**ANKRD29**	ankyrin repeat domain 29	-2.25	0.00601	-1.38	0.20437
**SPRED2**	sprouty-related, EVH1 domain containing 2	-2.24	0.03321	-1.13	0.71683
**SLC30A1**	solute carrier family 30 (zinc transporter), member 1	-2.24	0.00011	-1.61	0.00516
**VKORC1L1**	vitamin K epoxide reductase complex, subunit 1-like 1	-2.24	0.0034	-1.55	0.06868
**GJC1**	gap junction protein, gamma 1, 45kDa	-2.24	0.02626	1.88	0.0706
**RFC2**	replication factor C (activator 1) 2, 40kDa	-2.24	0.00028	-1.25	0.16756
**HPCAL1**	hippocalcin-like 1	-2.24	0.00325	-1.43	0.11893
**DPYSL3**	dihydropyrimidinase-like 3	-2.24	0.00301	-1.15	0.51253
**NQO1**	NAD(P)H dehydrogenase, quinone 1	-2.24	0.00109	-1.5	0.04684
**ZNF227**	zinc finger protein 227	-2.24	0.03937	-1.66	0.16846
**C12orf60**	chromosome 12 open reading frame 60	-2.24	0.00625	1.15	0.5622
**RFC5**	replication factor C (activator 1) 5, 36.5kDa	-2.24	0.02702	1.24	0.50596
**CENPJ**	centromere protein J	-2.24	0.01464	-1.19	0.54285
**TSNAX**	translin-associated factor X	-2.24	0.01814	-1.45	0.22274
**SGK494**	uncharacterized serine/threonine-protein kinase SgK494	-2.24	0.0028	-1.06	0.78249
**BATF3**	basic leucine zipper transcription factor, ATF-like 3	-2.24	0.02917	1.09	0.79182
**RAB27A**	RAB27A, member RAS oncogene family	-2.23	0.00712	-1.1	0.69552
**ITM2C**	integral membrane protein 2C	-2.23	0.00116	-1.48	0.05686
**MYO1B**	myosin IB	-2.23	0.00385	-1.14	0.55547
**DHRS13**	dehydrogenase/reductase (SDR family) member 13	-2.23	0.00021	1.3	0.09816
**PPM1J**	protein phosphatase, Mg2+/Mn2+ dependent, 1J	-2.23	0.00236	-1.15	0.49572
**NLN**	neurolysin (metallopeptidase M3 family)	-2.23	0.00424	1.21	0.41328
**PPT1**	palmitoyl-protein thioesterase 1	-2.23	1.00E-04	-1.77	0.00138
**CENPM**	centromere protein M	-2.23	0.01289	-1.23	0.45803
**PHF19**	PHD finger protein 19	-2.23	0.0144	-1.05	0.86182
**SNX3**	sorting nexin 3	-2.23	0.00377	-1.45	0.11734
**NCAPG2**	non-SMC condensin II complex, subunit G2	-2.23	0.00446	1.11	0.65583
**MFSD12**	major facilitator superfamily domain containing 12	-2.23	0.00065	1.28	0.17262
**SLC6A6**	solute carrier family 6 (neurotransmitter transporter, taurine), member 6	-2.23	0.00075	-1.5	0.03687
**BF432941**	---	-2.23	0.01014	-1.44	0.18532
**AL832803**	---	-2.23	0.00444	-1.05	0.82855
**KITLG**	KIT ligand	-2.23	0.00035	1.18	0.29968

**CORO1C**	coronin, actin binding protein, 1C	-2.23	0.00317	1.03	0.90464
**RTKN2**	rhotekin 2	-2.23	0.04338	-1.22	0.58403
**TMPO**	thymopoietin	-2.22	0.02857	1.07	0.8362
**ALKBH2**	alkB, alkylation repair homolog 2 (E. coli)	-2.22	0.03059	-1.13	0.71055
**KIAA1524**	KIAA1524	-2.22	0.0155	-1.25	0.44209
**PRPS2**	Phosphoribosyl pyrophosphate synthetase 2	-2.22	0.01436	1.26	0.40752
**TFR2**	transferrin receptor 2	-2.22	0.00478	1.89	0.0165
**TOP1MT**	topoisomerase (DNA) I, mitochondrial	-2.22	0.00256	-1.85	0.01217
**TMC7**	transmembrane channel-like 7	-2.22	0.00061	-1.21	0.27778
**ARSI**	arylsulfatase family, member I	-2.22	0.00063	-1.1	0.57633
**IRAK2**	interleukin-1 receptor-associated kinase 2	-2.22	0.00285	1.36	0.16075
**C3orf80**	chromosome 3 open reading frame 80	-2.22	0.02345	1.87	0.06251
**SORD**	sorbitol dehydrogenase	-2.22	0.00933	-1.1	0.70219
**MAPRE2**	microtubule-associated protein, RP/EB family, member 2	-2.22	0.03019	-1.09	0.79444
**ATP2B1**	ATPase, Ca++ transporting, plasma membrane 1	-2.22	0.01145	-1.18	0.54771
**TMEM205**	transmembrane protein 205	-2.22	0.02995	-1.13	0.70617
**KRT6B**	keratin 6B	-2.22	0.0132	-1.03	0.90177
**CARS**	cysteinyl-tRNA synthetase	-2.22	0.01605	1.38	0.27103
**TRAM2**	translocation associated membrane protein 2	-2.21	0.00268	-1.43	0.10677
**DTYMK**	deoxythymidylate kinase (thymidylate kinase)	-2.21	0.0375	-1.18	0.63197
**CCL20 ***	chemokine (C-C motif) ligand 20	-2.21	0.01529	3.63	0.00076
**ATP2A2**	ATPase, Ca++ transporting, cardiac muscle, slow twitch 2	-2.21	0.02918	-1.35	0.35864
**CHAF1A**	chromatin assembly factor 1, subunit A (p150)	-2.21	0.04633	-1.07	0.84735
**DBF4**	DBF4 homolog (S. cerevisiae)	-2.21	0.02998	1.09	0.79053
**GIT2**	G protein-coupled receptor kinase interacting ArfGAP 2	-2.21	0.00715	-1.12	0.64614
**ADAM19**	ADAM metallopeptidase domain 19	-2.21	0.01085	1.89	0.03121
**NTRK2**	neurotrophic tyrosine kinase, receptor, type 2	-2.21	0.02626	1.14	0.67468
**PURA**	purine-rich element binding protein A	-2.21	0.00904	-1.76	0.04505
**MIS18A**	MIS18 kinetochore protein homolog A (S. pombe)	-2.21	0.00038	-1.07	0.66733
**UBE4A**	ubiquitination factor E4A	-2.21	0.00016	-1.63	0.00495
**SLC44A1**	solute carrier family 44, member 1	-2.21	0.00111	-1.63	0.02018
**RNF157-AS1**	RNF157 antisense RNA 1 (non-protein coding)	-2.21	0.03193	-1.24	0.52195
**TCF7L2**	transcription factor 7-like 2 (T-cell specific, HMG-box)	-2.20	0.00939	-1.39	0.2117
**SAAL1**	serum amyloid A-like 1	-2.20	1.00E-04	1.03	0.84244
**FRMD5**	FERM domain containing 5	-2.20	0.04546	-1.56	0.22615
**AI807197**	---	-2.20	0.01074	-1.14	0.62055
**KRT86**	keratin 86	-2.20	0.00972	-1.19	0.51024
**PPP4R4**	protein phosphatase 4, regulatory subunit 4	-2.20	0.01076	-1.05	0.8398
**CDK17**	cyclin-dependent kinase 17	-2.20	0.00038	-2	0.00103
**AGMAT**	agmatine ureohydrolase (agmatinase)	-2.20	0.03134	1.15	0.66494
**TGOLN2**	trans-golgi network protein 2	-2.20	0.02572	-1.48	0.22278
**ENOX2**	ecto-NOX disulfide-thiol exchanger 2	-2.20	0.00178	-1.74	0.01448
**SNORA56**	small nucleolar RNA, H/ACA box 56	-2.20	2.00E-05	-1.1	0.39192
**LRRC8D**	leucine rich repeat containing 8 family, member D	-2.20	8.00E-05	-1.44	0.01527
**MAPKAPK2**	mitogen-activated protein kinase-activated protein kinase 2	-2.20	0.03525	-1.35	0.37997
**CDC20**	cell division cycle 20 homolog (S. cerevisiae)	-2.20	0.0152	-1.42	0.22698
**POLE2**	polymerase (DNA directed), epsilon 2, accessory subunit	-2.19	0.00305	1.38	0.14561

**SLC5A3**	solute carrier family 5 (sodium/myo-inositol cotransporter), member 3	-2.19	0.00064	-1.35	0.09497
**ACOT7**	acyl-CoA thioesterase 7	-2.19	0.01286	1.49	0.1606
**BRCA2**	breast cancer 2, early onset	-2.19	0.00337	-1.05	0.82534
**CD97**	CD97 molecule	-2.19	0.01929	-1.13	0.68602
**WBSCR22**	Williams Beuren syndrome chromosome region 22	-2.19	0.00539	-1.59	0.06411
**GATA6**	GATA binding protein 6	-2.19	0.00924	-1.08	0.75527
**APOE**	Apolipoprotein E	-2.19	0.03356	-1.52	0.21785
**TXNDC16**	thioredoxin domain containing 16	-2.19	0.01059	1.08	0.76037
**LIMCH1**	LIM and calponin homology domains 1	-2.19	1.00E-05	-1.03	0.79926
**AW362945**	---	-2.19	0.00149	1.05	0.78704
**BC040884**	---	-2.19	0.01115	1.05	0.84784
**C11orf9**	chromosome 11 open reading frame 9	-2.19	0.008	-1.68	0.05511
**PDXP**	pyridoxal (pyridoxine, vitamin B6) phosphatase	-2.19	0.01438	-1.19	0.53518
**BAG4**	BCL2-associated athanogene 4	-2.19	0.00817	-1.33	0.25824
**RPL31**	ribosomal protein L31	-2.19	0.00079	-1.35	0.10374
**HAUS1**	HAUS augmin-like complex, subunit 1	-2.18	0.00335	-1	0.99311
**IFT81**	intraflagellar transport 81 homolog (Chlamydomonas)	-2.18	0.00016	1.32	0.06921
**AI638063**	---	-2.18	0.03209	-1.29	0.44455
**PRIM2**	primase, DNA, polypeptide 2 (58kDa)	-2.18	0.02349	1.05	0.87948
**WAPAL**	wings apart-like homolog (Drosophila)	-2.18	0.01618	-1.36	0.28639
**AI672356**	---	-2.18	0.00827	-1.18	0.51216
**WDR35**	WD repeat domain 35	-2.18	0.00308	-1.21	0.3632
**GART**	phosphoribosylglycinamide formyltransferase, phosphoribosylglycinamide synthetase, phosphoribosylaminoimidazole synthetase	-2.18	0.00635	-1.64	0.05574
**AK021887**	---	-2.18	0.00157	1.06	0.74497
**BE551781**	---	-2.17	0.00067	-1.15	0.40243
**PLK1S1**	polo-like kinase 1 substrate 1	-2.17	0.00292	-1.37	0.14501
**N34514**	---	-2.17	0.04764	-1.18	0.64836
**PRDX3**	peroxiredoxin 3	-2.17	2.00E-05	-1.15	0.20936
**TRIB1**	tribbles homolog 1 (Drosophila)	-2.17	0.00769	-1.95	0.01698
**LOC100507535**	uncharacterized LOC100507535	-2.17	0.0046	-2.47	0.00172
**CASP9**	caspase 9, apoptosis-related cysteine peptidase	-2.17	4.00E-05	1.24	0.0897
**PXK**	PX domain containing serine/threonine kinase	-2.17	0.02051	1.02	0.95044
**LOC100505634**	uncharacterized LOC100505634	-2.17	0.03313	1.05	0.8707
**PPIF**	peptidylprolyl isomerase F	-2.17	6.00E-05	-1.94	0.00022
**ZNF37BP**	zinc finger protein 37B, pseudogene	-2.17	0.04363	1.09	0.79847
**AA523939**	---	-2.17	0.00911	1.64	0.06745
**AP2B1**	adaptor-related protein complex 2, beta 1 subunit	-2.17	0.00038	-1.44	0.03486
**CREB5 ***	cAMP responsive element binding protein 5	-2.17	0.02318	2.11	0.02696
**POLD3**	polymerase (DNA-directed), delta 3, accessory subunit	-2.17	0.00555	-1.01	0.96754
**ALG13**	asparagine-linked glycosylation 13 homolog (S. cerevisiae)	-2.16	0.04856	-1.08	0.83066
**LOC728613**	programmed cell death 6 pseudogene	-2.16	0.00034	-1.23	0.19204
**LEPREL4**	leprecan-like 4	-2.16	0.00053	-1.55	0.01858
**EXOSC2**	exosome component 2	-2.16	8.00E-05	-1.32	0.04517
**TBL2**	transducin (beta)-like 2	-2.16	0.00899	-1.56	0.0937
**SLC7A2**	solute carrier family 7 (cationic amino acid transporter, y+ system), member 2	-2.16	0.01212	1.11	0.68504
**CUL4A**	cullin 4A	-2.16	0.01549	-1.48	0.17069

**GNAS-AS1**	GNAS antisense RNA 1 (non-protein coding)	-2.16	0.02563	-1.19	0.56398
**EME1**	essential meiotic endonuclease 1 homolog 1 (S. pombe)	-2.16	0.02229	-1.35	0.32407
**RASSF10**	Ras association (RalGDS/AF-6) domain family (N-terminal) member 10	-2.16	0.01003	1.16	0.55756
**SOCS2 ***	suppressor of cytokine signaling 2	-2.16	0.01048	2.3	0.00678
**PER2**	period homolog 2 (Drosophila)	-2.16	0.03433	-1.46	0.25977
**MTMR2**	myotubularin related protein 2	-2.16	0.01277	-1.21	0.46671
**ADPRM**	ADP-ribose/CDP-alcohol diphosphatase, manganes	-2.16	0.03609	-1.61	0.16578
**PKMYT1**	protein kinase, membrane associated tyrosine/threonine 1	-2.16	0.002	-1.03	0.87025
**XK**	X-linked Kx blood group (McLeod syndrome)	-2.16	1.00E-04	1.07	0.62658
**RPRD1A**	regulation of nuclear pre-mRNA domain containing 1A	-2.16	0.01001	-1.77	0.04175
**HIST1H1A**	histone cluster 1, H1a	-2.16	0.00339	-1.35	0.16533
**MCM4**	minichromosome maintenance complex component 4	-2.16	0.02153	-1.24	0.46879
**AV700165**	---	-2.16	0.00874	1.14	0.6041
**NOP16**	NOP16 nucleolar protein homolog (yeast)	-2.16	0.02944	-1.33	0.37344
**RPL13P5**	ribosomal protein L13 pseudogene 5	-2.16	0.00609	-1.3	0.26351
**HOXA2**	homeobox A2	-2.16	0.00518	1.18	0.47046
**ANKRD27**	ankyrin repeat domain 27 (VPS9 domain)	-2.16	2.00E-05	-1.85	0.00012
**HYAL1**	hyaluronoglucosaminidase 1	-2.15	0.01291	-1.32	0.3061
**CASK**	calcium/calmodulin-dependent serine protein kinase (MAGUK family)	-2.15	0.00644	-1.2	0.44065
**PCP4L1**	Purkinje cell protein 4 like 1	-2.15	0.00098	-1.05	0.76425
**DUSP1**	Dual specificity phosphatase 1	-2.15	0.03823	-1.1	0.77805
**STEAP1B**	STEAP family member 1B	-2.15	0.00217	-1.09	0.65694
**ZWILCH**	Zwilch, kinetochore associated, homolog (Drosophila)	-2.15	0.01575	1.11	0.70745
**SLC37A3**	solute carrier family 37 (glycerol-3-phosphate transporter), member 3	-2.15	0.00781	-1.56	0.08471
**HSD17B12**	hydroxysteroid (17-beta) dehydrogenase 12	-2.14	0.00923	1.03	0.90883
**GOSR2**	golgi SNAP receptor complex member 2	-2.14	0.03711	-1.43	0.2883
**THSD4**	thrombospondin, type I, domain containing 4	-2.14	0.01684	1.1	0.73467
**UBA2**	ubiquitin-like modifier activating enzyme 2	-2.14	0.01256	-1.35	0.2591
**SAMD8**	sterile alpha motif domain containing 8	-2.14	0.04886	1.05	0.89641
**FZD4**	frizzled family receptor 4	-2.14	0.00046	-1.15	0.37176
**BZW1**	basic leucine zipper and W2 domains 1	-2.14	0.00568	-1.19	0.43636
**C1orf198**	chromosome 1 open reading frame 198	-2.14	0.01711	-1.02	0.93921
**DCAF13**	DDB1 and CUL4 associated factor 13	-2.14	0.00442	-1.36	0.17158
**SCD**	stearoyl-CoA desaturase (delta-9-desaturase)	-2.14	0.04806	1.08	0.83268
**NAMPT**	nicotinamide phosphoribosyltransferase	-2.14	0.00475	1.05	0.80757
**TFDP2**	transcription factor Dp-2 (E2F dimerization partner 2)	-2.14	0.02505	1.26	0.44406
**TRIB2**	tribbles homolog 2 (Drosophila)	-2.13	0.04787	-1.04	0.91538
**MMP1**	matrix metallopeptidase 1 (interstitial collagenase)	-2.13	0.00423	-1.02	0.91305
**UBE2T**	ubiquitin-conjugating enzyme E2T (putative)	-2.13	0.00971	1.1	0.70586
**BF445387**	---	-2.13	0.00411	-1.09	0.68739
**APLP1**	amyloid beta (A4) precursor-like protein 1	-2.13	0.00032	1.16	0.33678
**ZNF473**	zinc finger protein 473	-2.13	0.04825	-1.61	0.1864
**FLJ11871**	hypothetical protein FLJ11871	-2.13	0.04852	-1.96	0.07438

**SLC2A14**	solute carrier family 2 (facilitated glucose transporter), member 14	-2.13	0.00724	1.45	0.13219
**IGF2BP2**	insulin-like growth factor 2 mRNA binding protein 2	-2.13	0.00054	-1.28	0.14498
**USP54**	ubiquitin specific peptidase 54	-2.13	0.01173	-1.3	0.31978
**AW611560**	---	-2.13	0.00268	1.08	0.71556
**TMF1**	TATA element modulatory factor 1	-2.13	0.00277	-1.36	0.14701
**BCL2**	B-cell CLL/lymphoma 2	-2.13	0.03334	-1.1	0.77085
**DST**	dystonin	-2.13	0.01481	-1.17	0.55258
**BACE1**	beta-site APP-cleaving enzyme 1	-2.13	0.02133	-1.41	0.24932
**AA825721**	---	-2.13	0.01918	1.04	0.88351
**ITGA3**	integrin, alpha 3 (antigen CD49C, alpha 3 subunit of VLA-3 receptor)	-2.13	0.00489	-1.33	0.21263
**PARPBP**	PARP1 binding protein	-2.13	0.02254	-1.29	0.3817
**CSNK1E**	casein kinase 1, epsilon	-2.13	0.00075	-1.26	0.17844
**PVR**	poliovirus receptor	-2.12	0.00169	-1.3	0.17956
**AK094711**	---	-2.12	0.01695	1.23	0.44773
**TOP2A**	topoisomerase (DNA) II alpha 170kDa	-2.12	0.00545	-1.28	0.27155
**S100A13**	S100 calcium binding protein A13	-2.12	0.0352	-1.26	0.46861
**AA861608**	---	-2.12	0.00044	-1.21	0.23625
**RBM11**	RNA binding motif protein 11	-2.12	0.03091	-1.29	0.42476
**MTSS1**	metastasis suppressor 1	-2.12	0.02179	-1.14	0.65932
**ACER3**	alkaline ceramidase 3	-2.12	0.00174	-1.35	0.12654
**EFEMP2**	EGF containing fibulin-like extracellular matrix protein 2	-2.12	0.0274	1	0.99424
**CC2D2A**	coiled-coil and C2 domain containing 2A	-2.12	0.04185	-1.91	0.07349
**JDP2**	Jun dimerization protein 2	-2.12	0.01512	-1.22	0.46319
**SLC35F2**	solute carrier family 35, member F2	-2.12	6.00E-04	1.31	0.11413
**PEX13**	peroxisomal biogenesis factor 13	-2.12	0.02168	-1.08	0.79611
**AA532745**	---	-2.12	0.00979	-1.81	0.0313
**ADRB2**	adrenoceptor beta 2, surface	-2.12	0.03602	-1.35	0.35892
**NUP155**	nucleoporin 155kDa	-2.12	0.00081	1.1	0.57927
**NM_024853**	---	-2.12	0.03494	-1.03	0.9377
**C4orf46**	chromosome 4 open reading frame 46	-2.12	0.00087	-1.3	0.13945
**CBLB**	Cbl proto-oncogene, E3 ubiquitin protein ligase B	-2.11	0.00998	-1.08	0.76557
**AI356405**	---	-2.11	0.03733	-1.28	0.44278
**LOC283278**	uncharacterized LOC283278	-2.11	0.0027	-1.13	0.5417
**TJP2**	tight junction protein 2 (zona occludens 2)	-2.11	0.01622	1.23	0.43917
**UACA**	uveal autoantigen with coiled-coil domains and ankyrin repeats	-2.11	0.00398	-1.32	0.19649
**AI300571**	---	-2.11	0.04021	-1.95	0.06234
**TTC39C**	tetratricopeptide repeat domain 39C	-2.11	0.04239	-1.48	0.24892
**ATP2A1**	ATPase, Ca++ transporting, cardiac muscle, fast twitch 1	-2.11	0.04682	1.08	0.81904
**EPHA5**	EPH receptor A5	-2.11	0.01668	-1.04	0.8906
**TRAF3IP2-AS1**	TRAF3IP2 antisense RNA 1 (non-protein coding)	-2.11	0.00039	-1.44	0.02909
**WARS**	tryptophanyl-tRNA synthetase	-2.11	0.03212	1.23	0.5037
**NRIP3**	nuclear receptor interacting protein 3	-2.11	0.01216	-1.09	0.72272
**MFAP3**	microfibrillar-associated protein 3	-2.11	0.04492	-1.3	0.43557
**LOC100130357**	uncharacterized LOC100130357	-2.11	0.00492	1.07	0.76912
**RGS2**	regulator of G-protein signaling 2, 24kDa	-2.11	0.02556	1.22	0.50575
**GPN3**	GPN-loop GTPase 3	-2.11	0.04951	1.48	0.26851
**IKBIP**	IKBKB interacting protein	-2.11	0.01915	-1.17	0.57842
**ADCY1**	adenylate cyclase 1 (brain)	-2.10	0.02722	1.07	0.81966

**TMEM5**	transmembrane protein 5	-2.10	0.03205	-1.52	0.19151
**AK4**	adenylate kinase 4	-2.10	0.00485	1.11	0.63144
**ALG14**	asparagine-linked glycosylation 14 homolog (S. cerevisiae)	-2.10	0.02983	-1.57	0.15398
**WDR5**	WD repeat domain 5	-2.10	0.03197	-1.11	0.73922
**LOC100505880**	uncharacterized LOC100505880	-2.10	0.00269	1.1	0.62417
**METTL1**	methyltransferase like 1	-2.10	0.00493	-1.05	0.80711
**HELLS**	helicase, lymphoid-specific	-2.10	0.00621	1.12	0.61808
**FANCD2**	Fanconi anemia, complementation group D2	-2.10	0.01249	-1.09	0.72181
**FZD7**	frizzled family receptor 7	-2.10	0.02181	-1.17	0.5862
**CNKSR2**	connector enhancer of kinase suppressor of Ras 2	-2.10	0.04231	1.38	0.33922
**YWHAZ**	tyrosine 3-monooxygenase/tryptophan 5-monooxygenase activation protein, zeta polypeptide	-2.10	0.00407	-1.34	0.17698
**AI286239**	---	-2.10	0.0135	-1.45	0.16327
**TRIL**	TLR4 interactor with leucine-rich repeats	-2.09	0.00287	1.19	0.38747
**AW779917**	---	-2.09	0.00244	-1.55	0.0382
**LOC100128653**	uncharacterized LOC100128653	-2.09	0.00161	-1.23	0.26237
**SLC16A7**	solute carrier family 16, member 7 (monocarboxylic acid transporter 2)	-2.09	0.00426	-1.08	0.72202
**ENOSF1**	enolase superfamily member 1	-2.09	0.00308	1.1	0.61494
**NEURL1B**	neuralized homolog 1B (Drosophila)	-2.09	0.02728	1.24	0.46796
**FAM131B**	family with sequence similarity 131, member B	-2.09	0.00325	-1.07	0.71885
**DDX18**	DEAD (Asp-Glu-Ala-Asp) box polypeptide 18	-2.09	1.00E-04	-1.87	0.00038
**SIX4**	SIX homeobox 4	-2.09	0.00031	-1.17	0.27764
**NMNAT1**	nicotinamide nucleotide adenylyltransferase 1	-2.09	0.04481	-1.39	0.3366
**ETS1**	v-ets erythroblastosis virus E26 oncogene homolog 1 (avian)	-2.09	0.0024	-1.17	0.40876
**PRR11**	proline rich 11	-2.09	0.00017	-1.56	0.00618
**ARHGEF39**	Rho guanine nucleotide exchange factor (GEF) 39	-2.09	0.03228	-1.14	0.67624
**TOR3A**	torsin family 3, member A	-2.09	0.01209	-1.16	0.54897
**CSPG5**	chondroitin sulfate proteoglycan 5 (neuroglycan C)	-2.09	0.04667	1.7	0.13319
**NCAPD2**	non-SMC condensin I complex, subunit D2	-2.09	0.02831	-1.31	0.3657
**OGT**	O-linked N-acetylglucosamine (GlcNAc) transferase	-2.09	0.01695	-1.49	0.15551
**ITPRIP**	inositol 1,4,5-trisphosphate receptor interacting protein	-2.09	0.00459	-1.07	0.73413
**ZSCAN12P1**	zinc finger and SCAN domain containing 12 pseudogene 1	-2.09	0.01342	1.03	0.9222
**ARHGEF2**	Rho/Rac guanine nucleotide exchange factor (GEF) 2	-2.08	0.01164	1.05	0.82975
**TBCE**	tubulin folding cofactor E	-2.08	9.00E-04	-1.55	0.01994
**WWC2**	WW and C2 domain containing 2	-2.08	0.02987	-1.91	0.04991
**NCEH1**	neutral cholesterol ester hydrolase 1	-2.08	0.00352	-1.83	0.01074
**MCOLN2**	mucolipin 2	-2.08	0.00296	-1.07	0.72433
**NR4A1**	nuclear receptor subfamily 4, group A, member 1	-2.08	7.00E-05	1.06	0.63157
**APOC1**	apolipoprotein C-I	-2.08	0.0031	-1.11	0.58266
**MPP6**	membrane protein, palmitoylated 6 (MAGUK p55 subfamily member 6)	-2.08	0.00045	-1.34	0.07265
**RECQL4**	RecQ protein-like 4	-2.08	0.04024	1.01	0.98139
**AUNIP**	aurora kinase A and ninein interacting protein	-2.08	0.02833	-1.19	0.55395
**GLT8D1**	Glycosyltransferase 8 domain containing 1	-2.08	0.03698	-1.03	0.93728
**LOC100289092**	uncharacterized LOC100289092	-2.08	0.02825	1.1	0.75448

**STRN**	striatin, calmodulin binding protein	-2.08	0.00099	-1.47	0.03665
**G3BP2**	GTPase activating protein (SH3 domain) binding protein 2	-2.08	0.02488	-1.26	0.42408
**ERGIC2**	ERGIC and golgi 2	-2.08	0.01276	-1.91	0.02396
**OSBPL6**	oxysterol binding protein-like 6	-2.08	0.00057	1.09	0.57641
**ETFA**	electron-transfer-flavoprotein, alpha polypeptide	-2.08	0.00011	-1.95	0.00023
**BF515158**	---	-2.08	0.00054	-1.3	0.10931
**EIF1AX**	eukaryotic translation initiation factor 1A, X-linked	-2.08	0.03204	1.05	0.85947
**CHST1**	carbohydrate (keratan sulfate Gal-6) sulfotransferase 1	-2.08	0.0052	1.1	0.66153
**BICC1**	bicaudal C homolog 1 (Drosophila)	-2.08	0.02366	1.12	0.68087
**LINC00669**	long intergenic non-protein coding RNA 669	-2.08	0.00229	-1.04	0.81659
**TEAD1**	TEA domain family member 1 (SV40 transcriptional enhancer factor)	-2.07	0.00388	-1.04	0.83987
**NFE2L3**	nuclear factor (erythroid-derived 2)-like 3	-2.07	0.00518	1.05	0.83404
**GZMB**	granzyme B (granzyme 2, cytotoxic T-lymphocyte-associated serine esterase 1)	-2.07	1.00E-04	-1.01	0.91354
**TRIM14**	tripartite motif containing 14	-2.07	0.0415	1.26	0.47306
**SDC2**	syndecan 2	-2.07	0.00019	1.09	0.51334
**WDR67**	WD repeat domain 67	-2.07	0.00094	-1.03	0.84153
**PPIH**	peptidylprolyl isomerase H (cyclophilin H)	-2.07	0.02623	-1.04	0.88104
**CHN1**	chimerin (chimaerin) 1	-2.07	0.00667	1.26	0.3083
**SLC25A45**	solute carrier family 25, member 45	-2.07	0.01745	1.71	0.06179
**ERP29**	endoplasmic reticulum protein 29	-2.07	6.00E-05	-1.87	0.00021
**SRPX2**	sushi-repeat containing protein, X-linked 2	-2.07	0.00409	-1.65	0.0292
**FEM1B**	fem-1 homolog b (C. elegans)	-2.07	0.02164	-1.32	0.32656
**EML4**	echinoderm microtubule associated protein like 4	-2.07	0.0045	-1.19	0.41811
**RASSF8**	Ras association (RalGDS/AF-6) domain family (N-terminal) member 8	-2.07	0.02099	-1.06	0.83329
**EPHA2**	EPH receptor A2	-2.07	0.00177	1.06	0.72688
**ASPM**	asp (abnormal spindle) homolog, microcephaly associated (Drosophila)	-2.07	0.00797	-1.69	0.03836
**SF3B3**	splicing factor 3b, subunit 3, 130kDa	-2.06	0.02853	-1.1	0.73432
**ZNF263**	zinc finger protein 263	-2.06	0.04543	-1.47	0.25046
**TBC1D16**	TBC1 domain family, member 16	-2.06	0.00465	-1.9	0.00963
**ZBTB9**	zinc finger and BTB domain containing 9	-2.06	0.03688	-1.2	0.56096
**MCM3**	minichromosome maintenance complex component 3	-2.06	0.0169	1.06	0.81579
**AA088388**	---	-2.06	0.01107	1.11	0.66161
**ZNF385C**	zinc finger protein 385C	-2.06	0.02141	-1.1	0.73741
**PEX10**	peroxisomal biogenesis factor 10	-2.06	0.03359	-1.33	0.35871
**DIAPH3**	diaphanous homolog 3 (Drosophila)	-2.06	0.02926	-1.02	0.93635
**DHX8**	DEAH (Asp-Glu-Ala-His) box polypeptide 8	-2.06	0.01829	-1.74	0.05665
**SH3BP5**	SH3-domain binding protein 5 (BTK-associated)	-2.06	0.00039	-1.04	0.78127
**ELMO1**	engulfment and cell motility 1	-2.06	0.00336	-1.05	0.81557
**ARTN**	artemin	-2.06	0.00453	-1.27	0.25824
**FAM198B**	family with sequence similarity 198, member B	-2.06	0.00012	1.12	0.37748
**RPE**	ribulose-5-phosphate-3-epimerase	-2.06	0.00109	-1.33	0.11126
**CPD**	carboxypeptidase D	-2.06	0.00087	-1.11	0.51506
**SMYD5**	SMYD family member 5	-2.06	0.04089	-1.9	0.06403
**ARL5A**	ADP-ribosylation factor-like 5A	-2.06	0.0119	-1.3	0.29514
**EIF1**	eukaryotic translation initiation factor 1	-2.06	0.00275	-1.14	0.50123
**ZEB1-AS1**	ZEB1 antisense RNA 1 (non-protein coding)	-2.06	0.04228	-1.2	0.56652

**TNFRSF11A**	tumor necrosis factor receptor superfamily, member 11a, NFKB activator	-2.06	0.02594	1.26	0.42857
**THNSL2**	threonine synthase-like 2 (S. cerevisiae)	-2.06	0.00108	-1.04	0.7973
**ACLY**	ATP citrate lyase	-2.05	0.00024	-1.02	0.90364
**LOC389641**	uncharacterized LOC389641	-2.05	0.01517	1.05	0.84144
**HSF2BP**	heat shock transcription factor 2 binding protein	-2.05	0.01388	-1.04	0.88789
**CENPN**	centromere protein N	-2.05	0.00782	-1.14	0.57236
**ITPRIPL2**	inositol 1,4,5-trisphosphate receptor interacting protein-like 2	-2.05	0.0014	1.07	0.68963
**AA026390**	---	-2.05	0.00039	-1.06	0.70233
**NCBP2**	nuclear cap binding protein subunit 2, 20kDa	-2.05	0.02411	-1.43	0.21531
**SNRPB**	small nuclear ribonucleoprotein polypeptides B and B1	-2.05	0.0381	-1.33	0.36939
**DSN1**	DSN1, MIND kinetochore complex component, homolog (S. cerevisiae)	-2.05	0.02104	-1.05	0.87008
**SSFA2**	sperm specific antigen 2	-2.05	0.03658	-1.42	0.27024
**FAM3C**	family with sequence similarity 3, member C	-2.05	0.01	-1.25	0.34797
**AA213799**	---	-2.05	0.01688	1.01	0.95778
**GPR37**	G protein-coupled receptor 37 (endothelin receptor type B-like)	-2.05	0.00053	-1.19	0.26906
**BBIP1**	BBSome interacting protein 1	-2.05	0.04477	-1.02	0.96111
**EGR3**	early growth response 3	-2.05	0.00916	-1.01	0.95385
**WHSC1**	Wolf-Hirschhorn syndrome candidate 1	-2.05	0.04454	-1.13	0.69595
**ZDHHC23**	zinc finger, DHHC-type containing 23	-2.05	0.00567	1.07	0.7429
**PSD3**	pleckstrin and Sec7 domain containing 3	-2.05	0.0173	1.15	0.59378
**CDRT1**	CMT1A duplicated region transcript 1	-2.04	0.00933	1.01	0.97279
**RHOA**	ras homolog family member A	-2.04	0.03555	-1.25	0.46985
**LINC00184**	long intergenic non-protein coding RNA 184	-2.04	0.04128	-1.23	0.52202
**ASIC1**	acid-sensing (proton-gated) ion channel 1	-2.04	0.00991	1.19	0.46583
**DDX11L2**	DEAD/H (Asp-Glu-Ala-Asp/His) box helicase 11 like 2	-2.04	0.04299	-1.13	0.7063
**EML6**	echinoderm microtubule associated protein like 6	-2.04	0.01769	1.49	0.14757
**F3**	coagulation factor III (thromboplastin, tissue factor)	-2.04	0.00206	1.09	0.62391
**SP110**	SP110 nuclear body protein	-2.04	0.03737	1.04	0.89555
**CLPB**	ClpB caseinolytic peptidase B homolog (E. coli)	-2.04	0.02817	-1.22	0.49816
**COMMD10**	COMM domain containing 10	-2.04	0.00911	-1.59	0.06484
**FAM49A**	family with sequence similarity 49, member A	-2.04	0.00519	-1.05	0.82492
**PARP1**	poly (ADP-ribose) polymerase 1	-2.04	0.00028	-1.27	0.09999
**SERINC2**	serine incorporator 2	-2.04	0.0145	1.1	0.71506
**MTBP**	Mdm2, transformed 3T3 cell double minute 2, p53 binding protein (mouse) binding protein, 104kDa	-2.04	0.00798	-1.23	0.35576
**AA601997**	---	-2.04	0.02956	1.06	0.85235
**SRP72**	signal recognition particle 72kDa	-2.04	0.00062	-1.48	0.02407
**AF070618**	---	-2.04	0.04162	-1.57	0.17052
**HIST1H4F**	histone cluster 1, H4f	-2.03	0.00567	-1.49	0.0779
**GFI1**	growth factor independent 1 transcription repressor	-2.03	0.00676	1.84	0.01521
**KLF4**	Kruppel-like factor 4 (gut)	-2.03	0.03448	1.36	0.3129
**GPR125**	G protein-coupled receptor 125	-2.03	0.03642	1.3	0.39706
**SYNCRIP**	synaptotagmin binding, cytoplasmic RNA interacting protein	-2.03	0.0199	-1.47	0.16585
**HAUS8**	HAUS augmin-like complex, subunit 8	-2.03	0.03245	-1.03	0.9241

**KIAA1958**	KIAA1958	-2.03	0.02018	-1.18	0.53279
**TPM3**	tropomyosin 3	-2.03	0.02308	-1.1	0.73508
**GALC**	galactosylceramidase	-2.03	0.00849	-1.09	0.7058
**STIL**	SCL/TAL1 interrupting locus	-2.03	0.01191	-1.04	0.86449
**HS3ST1**	heparan sulfate (glucosamine) 3-O-sulfotransferase 1	-2.03	0.01363	1.09	0.73021
**ELP5**	elongator acetyltransferase complex subunit 5	-2.03	0.01955	-1.35	0.26387
**SYS1**	SYS1 Golgi-localized integral membrane protein homolog (S. cerevisiae)	-2.03	0.00314	-1.33	0.15102
**URI1**	URI1, prefoldin-like chaperone	-2.03	0.01291	-1.57	0.0854
**NOL7**	nucleolar protein 7, 27kDa	-2.03	0.03974	-1.63	0.13478
**ITGA1**	integrin, alpha 1	-2.03	0.01548	1.08	0.75614
**WDR25**	WD repeat domain 25	-2.03	0.04827	1.04	0.89944
**AI382026**	---	-2.03	0.00126	-1.01	0.93655
**STAMBP**	STAM binding protein	-2.03	0.04001	-1.59	0.15265
**MFAP5 ***	microfibrillar associated protein 5	-2.03	0.01464	2.41	0.00118
**C19orf24**	chromosome 19 open reading frame 24	-2.03	0.0084	-1.86	0.01687
**MPP1**	membrane protein, palmitoylated 1, 55kDa	-2.02	0.00204	1.05	0.77192
**PCSK6**	proprotein convertase subtilisin/kexin type 6	-2.02	0.02436	-1.55	0.1328
**PPAN**	peter pan homolog (Drosophila)	-2.02	0.03712	-1.17	0.60591
**IL21R**	interleukin 21 receptor	-2.02	0.00279	-1.02	0.92278
**C20orf112**	chromosome 20 open reading frame 112	-2.02	0.04396	-1.41	0.28462
**EMC1**	ER membrane protein complex subunit 1	-2.02	0.00019	-1.65	0.00226
**ERLIN2**	ER lipid raft associated 2	-2.02	0.02035	-1.66	0.07512
**UCA1**	urothelial cancer associated 1 (non-protein coding)	-2.02	0.00278	1.68	0.01566
**FAM167A**	family with sequence similarity 167, member A	-2.02	0.0128	1.7	0.04469
**MBTPS2**	membrane-bound transcription factor peptidase, site 2	-2.02	0.00384	-1.47	0.06765
**TLN2**	talin 2	-2.01	0.04812	-1.12	0.72476
**DLGAP1-AS1**	DLGAP1 antisense RNA 1	-2.01	0.00014	-1.3	0.05454
**CDC25A**	cell division cycle 25 homolog A (S. pombe)	-2.01	0.03937	1.97	0.04454
**G2E3**	G2/M-phase specific E3 ubiquitin protein ligase	-2.01	0.02372	-1.74	0.06243
**B3GNT5**	UDP-GlcNAc:betaGal beta-1,3-N-acetylglucosaminyltransferase 5	-2.01	0.00479	1.07	0.75068
**ITGB5**	integrin, beta 5	-2.01	0.03345	1.54	0.15914
**DERL1**	derlin 1	-2.01	0.00076	-1.63	0.00775
**FAM100B**	family with sequence similarity 100, member B	-2.01	0.00195	-1.3	0.15831
**LZIC**	leucine zipper and CTNNBIP1 domain containing	-2.01	0.00122	-1.27	0.16251
**PAQR8**	progestin and adipoQ receptor family member VIII	-2.01	0.03285	1.23	0.48066
**NPM3**	nucleophosmin/nucleoplasmin 3	-2.01	0.00211	-1.1	0.59068
**MAOA**	monoamine oxidase A	-2.01	0.01365	1.01	0.95531
**GPATCH2L**	G patch domain containing 2-like	-2.01	0.01497	-1.18	0.50247
**BE742377**	---	-2.01	0.00467	-1.5	0.06284
**TBC1D9B**	TBC1 domain family, member 9B (with GRAM domain)	-2.01	0.01225	-1.66	0.05114
**CCDC68**	coiled-coil domain containing 68	-2.01	0.00297	-1.02	0.92354
**ASPHD2**	aspartate beta-hydroxylase domain containing 2	-2.01	0.00241	1.1	0.58007
**BC016787**	---	-2.01	0.00466	-1.76	0.01475
**NEDD9**	neural precursor cell expressed, developmentally down-regulated 9	-2.01	0.02511	-1.09	0.74749
**CHST7**	carbohydrate (N-acetylglucosamine 6-O) sulfotransferase 7	-2.01	0.03377	1.32	0.35605

**PLCL2**	phospholipase C-like 2	-2.01	0.00163	-1.21	0.27202
**SNAI1**	snail homolog 1 (Drosophila)	-2.01	0.00233	-1.24	0.24799
**TMEM64**	transmembrane protein 64	-2.01	0.01925	-1.5	0.13482
**PPP2R2C**	protein phosphatase 2, regulatory subunit B, gamma	-2.01	0.00452	1.81	0.01133
**GLA**	galactosidase, alpha	-2.00	0.007	-1.17	0.4602
**BIN1**	bridging integrator 1	-2.00	0.04147	-1.5	0.20228
**AI679555**	---	-2.00	5.00E-04	1.01	0.93089
**RRBP1**	ribosome binding protein 1 homolog 180kDa (dog)	-2.00	0.00888	-1.61	0.05157
**MSH5**	mutS homolog 5 (E. coli)	-2.00	0.01466	-1.31	0.28384
**BF003032**	---	-2.00	0.00325	1.19	0.36197
**LPCAT4**	lysophosphatidylcholine acyltransferase 4	-2.00	0.00768	1.3	0.24687
**TNFRSF10A**	tumor necrosis factor receptor superfamily, member 10a	-2.00	0.0172	1.07	0.79329
**UST**	uronyl-2-sulfotransferase	-2.00	0.01024	1.14	0.55528
**PRKAG2**	protein kinase, AMP-activated, gamma 2 non-catalytic subunit	-2.00	0.03961	-1.66	0.11467
**FGF13-AS1**	FGF13 antisense RNA 1	2.00	0.01298	1.1	0.68338
**N4BP2L1**	NEDD4 binding protein 2-like 1	2.00	0.00501	1.29	0.22239
**SMARCE1**	SWI/SNF related, matrix associated, actin dependent regulator of chromatin, subfamily e, member 1	2.00	0.01735	1.75	0.04514
**ABLIM2**	actin binding LIM protein family, member 2	2.00	0.00122	-1.22	0.24204
**AI003508**	---	2.00	0.02093	-1.02	0.93765
**ANKRD65**	ankyrin repeat domain 65	2.00	0.00034	1.08	0.5582
**VPS13D**	Vacuolar protein sorting 13 homolog D (S. cerevisiae)	2.00	0.00037	1.03	0.83058
**AU144961**	---	2.00	0.00069	1.21	0.21481
**C10orf116**	chromosome 10 open reading frame 116	2.01	0.00478	-1.01	0.95032
**MKRN1**	makorin ring finger protein 1	2.01	0.00893	1.36	0.19071
**AA195276**	---	2.01	0.0154	-1.02	0.92461
**AI832594**	---	2.01	0.02789	1.29	0.36881
**CCDC102B**	coiled-coil domain containing 102B	2.01	0.01925	-1.04	0.89214
**ACP5**	acid phosphatase 5, tartrate resistant	2.01	0.02793	-1.1	0.74457
**AU147291**	---	2.01	0.03791	-1.09	0.77455
**WDR77**	WD repeat domain 77	2.01	0.00308	1.11	0.57488
**HIST1H2AG**	histone cluster 1, H2ag	2.01	0.00181	1.88	0.00359
**AI761498**	---	2.01	0.00618	1.09	0.69168
**LOC158960**	uncharacterized protein BC009467	2.02	0.00023	-1.04	0.76944
**PIK3CB**	phosphoinositide-3-kinase, catalytic, beta polypeptide	2.02	0.04169	1.08	0.81566
**NUDT14**	nudix (nucleoside diphosphate linked moiety X)-type motif 14	2.02	0.00882	-1.27	0.29956
**AK096998**	---	2.03	0.00068	-1.01	0.95586
**PCDHB9**	protocadherin beta 9	2.03	0.00476	1.28	0.23385
**CD24**	CD24 molecule	2.03	0.00102	1.04	0.82659
**ABHD12**	abhydrolase domain containing 12	2.03	0.00054	-1.38	0.04816
**FAM168A**	family with sequence similarity 168, member A	2.03	0.00078	1.5	0.02156
**CREG2**	cellular repressor of E1A-stimulated genes 2	2.03	0.02738	1.17	0.57582
**LOC100996455**	uncharacterized LOC100996455	2.03	0.01343	-1.03	0.91661
**COBLL1**	COBL-like 1	2.03	0.00461	1.21	0.3618
**AL138240**	---	2.03	0.00219	-1.2	0.31961
**HOXA10**	homeobox A10	2.04	0.00012	1.38	0.02153

**FKBP3**	FK506 binding protein 3, 25kDa	2.04	0.00427	1.35	0.15381
**AK000792**	---	2.04	0.03573	1.25	0.46118
**AI733194**	---	2.04	0.00067	-1.14	0.39928
**GPR39**	G protein-coupled receptor 39	2.04	0.01846	-1.1	0.71956
**LOC283788**	FSHD region gene 1 pseudogene	2.04	0.00034	-1.24	0.14181
**NAIP**	NLR family, apoptosis inhibitory protein	2.04	0.00169	1.14	0.46342
**ALS2CR8**	amyotrophic lateral sclerosis 2 (juvenile) chromosome region, candidate 8	2.04	0.00411	1.23	0.31791
**C1orf101**	chromosome 1 open reading frame 101	2.04	0.00143	1.14	0.46068
**ADAM17**	ADAM metallopeptidase domain 17	2.05	0.00086	1.05	0.74106
**MIR4448**	microRNA 4448	2.05	0.00859	1.13	0.58873
**LOC254057**	uncharacterized LOC254057	2.05	0.00033	-1.03	0.80994
**ASMTL-AS1**	ASMTL antisense RNA 1 (non-protein coding)	2.05	0.00094	1.13	0.46551
**SIAE**	sialic acid acetylesterase	2.05	0.01643	1.75	0.04921
**FBXL14**	F-box and leucine-rich repeat protein 14	2.05	0.00563	1.33	0.19441
**C16orf53**	Chromosome 16 open reading frame 53	2.06	0.01534	-1.16	0.55377
**LOC550643**	uncharacterized LOC550643	2.06	2.00E-04	1.11	0.44152
**ACOX1**	acyl-CoA oxidase 1, palmitoyl	2.06	0.00013	1.33	0.04352
**BPNT1**	3'(2'), 5'-bisphosphate nucleotidase 1	2.06	0.00118	1.05	0.77688
**LOC283713**	uncharacterized LOC283713	2.06	0.01445	-1.01	0.98122
**AL137616**	---	2.06	0.01173	1.03	0.8916
**GGTLC2**	gamma-glutamyltransferase light chain 2	2.07	0.00272	-1.02	0.9107
**STAP2**	signal transducing adaptor family member 2	2.07	0.0029	1.05	0.79627
**HEG1**	HEG homolog 1 (zebrafish)	2.07	0.00062	1.76	0.00362
**AK026824**	---	2.07	0.00021	-1.1	0.47321
**GSN**	gelsolin	2.07	0.03916	1.4	0.30593
**C1orf85**	chromosome 1 open reading frame 85	2.08	0.01274	-1.62	0.07457
**PCDHB13**	protocadherin beta 13	2.08	0.01211	1.05	0.83425
**AK025218**	---	2.08	0.003	1.34	0.15435
**BF511336**	---	2.08	0.00188	1.01	0.96832
**PVRL1**	poliovirus receptor-related 1 (herpesvirus entry mediator C)	2.08	0.00444	1.23	0.32848
**TNS3**	tensin 3	2.08	0.00144	-1.5	0.03891
**LAGE3**	L antigen family, member 3	2.08	0.00098	1.42	0.05291
**PHYH**	phytanoyl-CoA 2-hydroxylase	2.09	0.00094	1.6	0.0146
**TRIM29**	tripartite motif containing 29	2.09	0.00459	1.38	0.14362
**PPP1R3F**	Protein phosphatase 1, regulatory subunit 3F	2.09	0.03058	-1.09	0.77736
**RASA4**	RAS p21 protein activator 4	2.09	0.00965	1.95	0.0161
**COL5A1**	collagen, type V, alpha 1	2.09	0.00254	1.17	0.42331
**RNFT1**	ring finger protein, transmembrane 1	2.09	0.01525	1.2	0.48369
**LOC92249**	uncharacterized LOC92249	2.09	0.0414	1.27	0.46341
**BU929456**	---	2.10	0.02389	1.49	0.18274
**LRRC70**	leucine rich repeat containing 70	2.10	0.00524	-1.26	0.29618
**ESYT1**	Extended synaptotagmin-like protein 1	2.10	0.00228	1	0.98191
**TYMP**	thymidine phosphorylase	2.10	0.00195	1.23	0.2731
**POU3F1**	POU class 3 homeobox 1	2.10	0.03782	-1.21	0.55021
**ZNF264**	zinc finger protein 264	2.11	0.00722	1.04	0.86
**SNRNP27**	small nuclear ribonucleoprotein 27kDa (U4/U6.U5)	2.11	0.00042	1.63	0.00705
**HEATR7A**	HEAT repeat containing 7A	2.11	0.00545	1.22	0.37226
**TRPM7**	transient receptor potential cation channel, subfamily M, member 7	2.12	0.01021	1.21	0.44209
**FCHSD2**	FCH and double SH3 domains 2	2.12	0.00012	1.82	0.00073

**PARD3B**	par-3 partitioning defective 3 homolog B (C. elegans)	2.13	0.00148	1.36	0.10947
**KLHL24**	kelch-like 24 (Drosophila)	2.13	0.00038	1	0.98012
**MORC2-AS1**	MORC2 antisense RNA 1 (non-protein coding)	2.13	6.00E-05	-1.12	0.37322
**LOC90246**	uncharacterized LOC90246	2.13	0.00525	1.08	0.73091
**SLC35D1**	solute carrier family 35 (UDP-glucuronic acid/UDP-N-acetylgalactosamine dual transporter), member D1	2.13	0.03689	1.16	0.65377
**BG232090**	---	2.13	0.01274	1.02	0.92906
**KRI1**	KRI1 homolog (S. cerevisiae)	2.14	0.00339	1.14	0.53505
**ECHDC2**	enoyl CoA hydratase domain containing 2	2.14	0.00084	1.31	0.13304
**PATL2**	protein associated with topoisomerase II homolog 2 (yeast)	2.14	0.00148	1.5	0.04419
**TPM1**	tropomyosin 1 (alpha)	2.14	0.00161	-1.03	0.87218
**AU157675**	---	2.14	0.00064	-1.06	0.71295
**BF724733**	---	2.14	0.00131	-1.06	0.74114
**C1QTNF3**	C1q and tumor necrosis factor related protein 3	2.14	0.04033	1.38	0.34722
**CXCR7**	Chemokine (C-X-C motif) receptor 7	2.14	0.0034	1.05	0.80132
**TSHZ1**	teashirt zinc finger homeobox 1	2.15	0.00687	1.02	0.94378
**AI223854**	---	2.15	0.00628	1.08	0.73394
**FLJ13197**	uncharacterized FLJ13197	2.15	0.04223	1.19	0.60376
**PCDHB5**	protocadherin beta 5	2.15	0.02824	1	0.98756
**WWOX**	WW domain containing oxidoreductase	2.15	0.00046	1.54	0.01682
**C17orf108**	chromosome 17 open reading frame 108	2.15	0.00208	1.03	0.86556
**PARM1**	prostate androgen-regulated mucin-like protein 1	2.16	0.00136	1	0.97831
**ZBTB20**	zinc finger and BTB domain containing 20	2.16	0.00686	1.15	0.56129
**PLCD3**	phospholipase C, delta 3	2.16	0.00288	1.05	0.79755
**UNC13D**	unc-13 homolog D (C. elegans)	2.16	8.00E-05	1.35	0.03391
**ATG4D**	autophagy related 4D, cysteine peptidase	2.16	0.00176	1.07	0.71567
**NBPF1**	neuroblastoma breakpoint family, member 1	2.17	0.02157	1.27	0.42214
**GATA3**	GATA binding protein 3	2.17	0.00095	-1.41	0.07244
**RASSF6**	Ras association (RalGDS/AF-6) domain family member 6	2.17	0.02682	-1.36	0.33041
**AA262084**	---	2.17	0.03946	-1.08	0.81551
**LOC728978**	uncharacterized LOC728978	2.18	0.02444	-1.15	0.64854
**ZMYM6**	zinc finger, MYM-type 6	2.18	0.00019	1.15	0.32879
**BDH2**	3-hydroxybutyrate dehydrogenase, type 2	2.18	1.00E-05	1.2	0.09172
**TTC18**	tetratricopeptide repeat domain 18	2.18	0.00182	1.28	0.21352
**AI298089**	---	2.18	0.0468	1.08	0.83562
**HSPB1**	heat shock 27kDa protein 1	2.18	0.02484	1.04	0.88568
**NKIRAS2**	NFKB inhibitor interacting Ras-like 2	2.19	0.00053	1.39	0.06596
**LRRC37A4P**	leucine rich repeat containing 37, member A4, pseu	2.19	0.00033	1.17	0.31018
**AU144676**	---	2.19	0.02347	1.3	0.3977
**AL049975**	---	2.19	0.0336	1.14	0.68317
**FLJ40852**	uncharacterized LOC285962	2.19	0.00078	1.13	0.47245
**SORCS2**	sortilin-related VPS10 domain containing receptor 2	2.19	0.00024	1.45	0.02582
**TNNT2**	troponin T type 2 (cardiac)	2.19	0.02191	-1.18	0.5735
**LIMK2**	LIM domain kinase 2	2.19	0.00035	1.46	0.03027
**PKP1**	plakophilin 1 (ectodermal dysplasia/skin fragility syndrome)	2.19	0.01695	1.23	0.47672
**AK025797**	---	2.19	0.00536	1.23	0.38601
**RIT1**	Ras-like without CAAX 1	2.19	0.00065	1.98	0.00174
**BF830560**	---	2.19	0.001	-1.22	0.28443

**USP15**	ubiquitin specific peptidase 15	2.20	0.00027	1.43	0.03219
**FOXP4**	forkhead box P4	2.20	0.00347	-1.03	0.90793
**TNNI2**	troponin I type 2 (skeletal, fast)	2.20	0.00176	1.02	0.93594
**CCDC171**	coiled-coil domain containing 171	2.21	0.04585	1.44	0.32171
**TMEM19**	transmembrane protein 19	2.21	0.00031	1.04	0.79873
**CYFIP2**	cytoplasmic FMR1 interacting protein 2	2.21	0.00134	-1.35	0.12695
**AA826176**	---	2.21	0.01913	1.27	0.42891
**C14orf164**	chromosome 14 open reading frame 164	2.21	0.00123	1.14	0.47351
**NTF4**	neurotrophin 4	2.21	0.0318	1.09	0.7921
**W60691**	---	2.21	0.00071	1.3	0.15038
**MSX2**	msh homeobox 2	2.22	0.02264	1.15	0.64938
**SLC31A2**	solute carrier family 31 (copper transporters), member 2	2.22	0.0313	1.46	0.26686
**BC038589**	---	2.23	0.01091	1.07	0.78592
**AW518747**	---	2.23	3.00E-05	-1.25	0.07916
**SCARF1**	scavenger receptor class F, member 1	2.23	0.0238	-1.08	0.8043
**EHD2**	EH-domain containing 2	2.24	0.00413	1.29	0.27936
**ARL6IP5**	ADP-ribosylation-like factor 6 interacting protein 5	2.24	2.00E-05	1.21	0.11617
**SPECC1**	sperm antigen with calponin homology and coiled-coil domains 1	2.24	0.03375	1.27	0.4909
**TPTE2P5**	transmembrane phosphoinositide 3-phosphatase and tensin homolog 2 pseudogene 5	2.24	0.02917	-1.01	0.98163
**AGAP11**	ankyrin repeat and GTPase domain Arf GTPase activating protein 11	2.24	0.0014	1.08	0.67841
**KANK1**	KN motif and ankyrin repeat domains 1	2.24	0.01039	1.07	0.78857
**GTF2IRD2**	GTF2I repeat domain containing 2	2.25	0.04855	1.07	0.85271
**JAG2**	jagged 2	2.25	0.00069	1.2	0.30957
**CLCN4**	chloride channel, voltage-sensitive 4	2.25	0.00045	-1.06	0.7175
**RPL10**	ribosomal protein L10	2.25	0.00086	1.23	0.26847
**BF055351**	---	2.25	0.00457	1.12	0.61965
**TOLLIP**	toll interacting protein	2.26	0.00363	1.25	0.32907
**MTCH2**	mitochondrial carrier 2	2.26	3.00E-05	1.23	0.10178
**CAB39L**	calcium binding protein 39-like	2.26	0.02214	1	0.98838
**NUDT19**	nudix (nucleoside diphosphate linked moiety X)-type motif 19	2.26	8.00E-05	1.8	0.00103
**R37728**	---	2.26	0.03292	1.08	0.83026
**PIK3R2**	phosphoinositide-3-kinase, regulatory subunit 2 (beta)	2.26	0.00135	1.01	0.94671
**ID2**	inhibitor of DNA binding 2, dominant negative helix-loop-helix protein	2.27	0.00677	1.5	0.12531
**AMT**	aminomethyltransferase	2.27	7.00E-05	1.03	0.82082
**PPIEL**	peptidylprolyl isomerase E-like pseudogene	2.28	0.01001	1.02	0.93498
**C5orf46**	chromosome 5 open reading frame 46	2.28	0.00056	-1.15	0.42013
**MARVELD1**	MARVEL domain containing 1	2.29	0.00019	1.24	0.17822
**DQX1**	DEAQ box RNA-dependent ATPase 1	2.29	0.0023	1.48	0.08801
**TJP3**	tight junction protein 3 (zona occludens 3)	2.29	6.00E-05	-1.06	0.66876
**COL18A1**	collagen, type XVIII, alpha 1	2.29	0.01342	1.23	0.46997
**DECR1**	2,4-dienoyl CoA reductase 1, mitochondrial	2.30	8.00E-05	1.21	0.17307
**ABCC10**	ATP-binding cassette, sub-family C (CFTR/MRP), member 10	2.30	0.00895	1.39	0.23724
**MACROD2**	MACRO domain containing 2	2.30	0.01558	1.19	0.56634
**NODAL**	nodal homolog (mouse)	2.30	0.00052	-1.07	0.70918
**AW576443**	---	2.30	0.00097	1.36	0.12558

**AI990613**	---	2.31	0.033	1.04	0.91665
**IFITM10**	interferon induced transmembrane protein 10	2.31	0.02374	-1.43	0.28573
**NRF1**	nuclear respiratory factor 1	2.31	9.00E-05	1.01	0.96325
**PER3**	period homolog 3 (Drosophila)	2.31	0.00025	1.18	0.31749
**KRTAP19-1**	keratin associated protein 19-1	2.32	0.00791	1.16	0.56431
**TP53INP1**	tumor protein p53 inducible nuclear protein 1	2.32	0.00257	1.44	0.11363
**TMCC1**	transmembrane and coiled-coil domain family 1	2.32	2.00E-04	1.56	0.01398
**AK021983**	---	2.32	0.00403	1.12	0.62022
**AI797043**	---	2.32	0.00903	1.43	0.20276
**LRTOMT**	leucine rich transmembrane and 0-methyltransferase domain containing	2.33	0.00486	1.02	0.93902
**AU146764**	---	2.33	0.00319	-1.07	0.75727
**ARHGEF7**	Rho guanine nucleotide exchange factor (GEF) 7	2.34	0.00046	-1.02	0.89421
**KRTAP19-3**	keratin associated protein 19-3	2.34	0.03429	1.01	0.96693
**TAGLN**	transgelin	2.34	0.03019	-1.39	0.34995
**ALS2CL**	ALS2 C-terminal like	2.34	0.00318	1.31	0.25332
**ZNF652**	zinc finger protein 652	2.35	0.01964	-1	0.99832
**DUSP5P**	dual specificity phosphatase 5 pseudogene	2.35	0.00587	1.29	0.32585
**SYT8**	synaptotagmin VIII	2.35	0.00062	1.03	0.88476
**AI369187**	---	2.35	0.01507	1.32	0.3678
**AW419344**	---	2.35	0.00697	-1.1	0.72472
**TPTE2P6**	transmembrane phosphoinositide 3-phosphatase and tensin homolog 2 pseudogene 6	2.35	0.00653	1.11	0.69569
**MKNK2**	MAP kinase interacting serine/threonine kinase 2	2.37	0.0013	1.64	0.03092
**TRIM73**	tripartite motif containing 73	2.37	0.00371	-1.17	0.51377
**HYI**	hydroxypyruvate isomerase (putative)	2.37	3.00E-05	1.24	0.10149
**BF507518**	---	2.37	0.00321	1.33	0.24153
**TAPBP**	TAP binding protein (tapasin)	2.37	0.00028	1.33	0.10536
**R76828**	---	2.37	0.0014	1.31	0.20241
**KCNIP3**	Kv channel interacting protein 3, calsenilin	2.37	0.00315	1.13	0.59461
**AU146493**	---	2.38	0.00349	1.3	0.28289
**R62588**	---	2.38	0.03948	-1.41	0.37184
**SLC7A8**	solute carrier family 7 (amino acid transporter light chain, L system), member 8	2.38	0.00423	1.22	0.41428
**BC014318**	---	2.39	0.00048	1.25	0.2264
**MAP3K12**	mitogen-activated protein kinase kinase kinase 12	2.40	0.00276	1.34	0.21773
**ARNT**	aryl hydrocarbon receptor nuclear translocator	2.40	0.03055	1.19	0.62804
**N25555**	---	2.40	0.01166	1.74	0.08128
**LOC158696**	uncharacterized LOC158696	2.40	0.01369	1.12	0.71499
**ADHFE1**	alcohol dehydrogenase, iron containing, 1	2.41	0.00011	1.46	0.02704
**TP53AIP1**	tumor protein p53 regulated apoptosis inducing protein 1	2.42	0.0429	-1.17	0.68548
**AI002328**	---	2.42	0.00014	1.08	0.61826
**QKI**	QKI, KH domain containing, RNA binding	2.42	8.00E-05	-1.17	0.29872
**TMLHE-AS1**	TMLHE antisense RNA 1	2.42	0.00013	1.15	0.37539
**PPM1L**	protein phosphatase, Mg2+/Mn2+ dependent, 1L	2.42	5.00E-04	1.33	0.13923
**DNASE1L1**	deoxyribonuclease I-like 1	2.42	4.00E-05	1.17	0.24881
**ENPP5**	ectonucleotide pyrophosphatase/phosphodiesterase 5 (putative)	2.42	4.00E-05	-1.04	0.7721
**CHADL**	chondroadherin-like	2.43	0.00027	1.12	0.52001
**SEC31B**	SEC31 homolog B (S. cerevisiae)	2.43	0.01562	1.09	0.785
**C5orf56**	chromosome 5 open reading frame 56	2.43	0.00029	1.47	0.04385
**FBXO32**	F-box protein 32	2.44	0.02761	1.18	0.63639
**SMPDL3A**	sphingomyelin phosphodiesterase, acid-like 3A	2.45	0.00035	1.64	0.01603

**RNA45S5**	RNA, 45S pre-ribosomal 5	2.45	0.0064	1.23	0.44628
**AI097501**	---	2.46	0.00085	-1.03	0.87228
**AW976035**	---	2.46	0.03597	1.02	0.95553
**BMF**	Bcl2 modifying factor	2.46	0.0078	1.08	0.78909
**AW189097**	---	2.46	0.03749	1.02	0.95771
**ARMCX1**	armadillo repeat containing, X-linked 1	2.47	0.00665	1.09	0.75567
**CRYL1**	crystallin, lambda 1	2.47	0.0071	1.64	0.09685
**ADAMTS5 ***	ADAM metallopeptidase with thrombospondin type 1 motif, 5	2.47	0.00743	-2.26	0.01801
**PRICKLE1**	prickle homolog 1 (Drosophila)	2.48	0.0021	1.34	0.21789
**UHRF1BP1L**	UHRF1 binding protein 1-like	2.48	3.00E-05	1.38	0.03248
**LOC100129890**	similar to hCG1750329	2.48	0.00643	1.05	0.85927
**AKAP9**	A kinase (PRKA) anchor protein (yotiao) 9	2.49	0.00012	-1.09	0.58062
**CYP1B1**	cytochrome P450, family 1, subfamily B, polypeptide 1	2.49	0.00047	1.72	0.01311
**ERC1**	ELKS/RAB6-interacting/CAST family member 1	2.50	0.00103	-1.11	0.60298
**ARHGAP24**	Rho GTPase activating protein 24	2.50	0.001	-1.12	0.59272
**TMEM178A**	transmembrane protein 178A	2.52	0.02309	1.27	0.50566
**MARCKS**	myristoylated alanine-rich protein kinase C substrate	2.52	0.0027	1.07	0.78962
**EPHA4**	EPH receptor A4	2.53	0.02907	1.06	0.87192
**MACC1**	metastasis associated in colon cancer 1	2.53	0.0175	-1.03	0.93825
**ZNF277**	zinc finger protein 277	2.53	0.02947	1.24	0.56981
**RCAN2**	regulator of calcineurin 2	2.54	0.00063	1.04	0.82534
**ARID5B**	AT rich interactive domain 5B (MRF1-like)	2.54	0.01386	-1	0.9977
**LOC284837**	uncharacterized LOC284837	2.55	0.01852	1.06	0.86776
**AK022004**	---	2.55	0.00027	1.21	0.29197
**HOXD9**	homeobox D9	2.56	0.00068	-1.32	0.18692
**SLCO6A1**	solute carrier organic anion transporter family, member 6A1	2.56	0.00157	1.24	0.35535
**HOXD10**	homeobox D10	2.56	6.00E-05	1.69	0.00434
**GRAMD2**	GRAM domain containing 2	2.56	1.00E-05	1.23	0.08914
**BMP7**	bone morphogenetic protein 7	2.57	0.00327	1.14	0.60026
**EME2**	essential meiotic endonuclease 1 homolog 2 (S. pombe)	2.57	1.00E-05	1.04	0.74187
**AI287817**	---	2.57	0.01101	1.21	0.5544
**LRRN4CL**	LRRN4 C-terminal like	2.58	0.00483	1.18	0.54147
**HSPB3**	heat shock 27kDa protein 3	2.58	0.01175	1.09	0.78095
**MYLK**	myosin light chain kinase	2.58	0.00098	-1.13	0.57064
**N35099**	---	2.58	0.00375	1.18	0.5246
**SNRPN**	small nuclear ribonucleoprotein polypeptide N	2.59	0.00037	1.21	0.32605
**LOC338651**	uncharacterized LOC338651	2.60	0.02727	1.26	0.55128
**AL110133**	---	2.61	0.00103	-1.15	0.53153
**ARHGAP22**	Rho GTPase activating protein 22	2.61	0.00308	-1.05	0.84621
**COX7B**	cytochrome c oxidase subunit VIIb	2.61	3.00E-05	1.51	0.01156
**ZNF503-AS1**	ZNF503 antisense RNA 1 (non-protein coding)	2.62	0.00131	1.06	0.78335
**AL833462**	---	2.62	0.00017	1.39	0.07922
**HSPB2**	heat shock 27kDa protein 2	2.62	0.00016	-1.04	0.81823
**IGHG3**	immunoglobulin heavy constant gamma 3 (G3m ma	2.63	0.00409	-1.07	0.81139
**SNHG7**	small nucleolar RNA host gene 7 (non-protein coding)	2.64	0.00013	-1.3	0.13732
**PRODH**	proline dehydrogenase (oxidase) 1	2.66	0.01094	1.65	0.14232
**APCDD1**	adenomatosis polyposis coli down-regulated 1	2.67	0.00134	1.06	0.80664
**PLA2R1 ***	phospholipase A2 receptor 1, 180kDa	2.68	0.00043	-2.03	0.00428

**BAG1**	BCL2-associated athanogene	2.68	0.00699	-1.33	0.35244
**ZMYM3**	zinc finger, MYM-type 3	2.69	0.00149	1.38	0.19142
**PIK3C2B**	phosphoinositide-3-kinase, class 2, beta polypeptide	2.70	2.00E-05	1.9	8.00E-04
**LOC100859930**	uncharacterized LOC100859930	2.70	0.00485	-1.09	0.76229
**AI421234**	---	2.71	0.00033	-1.08	0.69165
**AI612775**	---	2.72	0.00671	1.09	0.77381
**CDHR1**	cadherin-related family member 1	2.72	0.00197	-1.05	0.85695
**HMCN1**	hemicentin 1	2.72	0.00695	1.17	0.61087
**NRBP2**	nuclear receptor binding protein 2	2.73	4.00E-05	1.7	0.00505
**PLCD4**	phospholipase C, delta 4	2.74	0.02692	1.02	0.95945
**CXorf56**	chromosome X open reading frame 56	2.74	0.00029	-1.24	0.2819
**AI686664**	---	2.75	1.00E-04	-1.37	0.08707
**SERPINF1**	serpin peptidase inhibitor, clade F (alpha-2 antiplasmin, pigment epithelium derived factor), member 1	2.76	0.0021	-1.06	0.83247
**ANXA4**	annexin A4	2.76	0.00047	1.71	0.02361
**LOC100507117**	uncharacterized LOC100507117	2.77	0.01431	-1.01	0.98515
**OVGP1**	oviductal glycoprotein 1, 120kDa	2.78	8.00E-05	-1.1	0.57301
**DLX5**	distal-less homeobox 5	2.78	0.00562	-1.15	0.64782
**DGCR11**	DiGeorge syndrome critical region gene 11 (non-protein coding)	2.79	3.00E-05	1.29	0.11696
**NREP**	neuronal regeneration related protein homolog (rat)	2.79	9.00E-05	1.55	0.02402
**W96062**	---	2.82	0.01089	1.69	0.14607
**ATP6V1G1**	ATPase, H+ transporting, lysosomal 13kDa, V1 subunit G1	2.84	0.00023	1.5	0.05582
**MAF**	v-maf musculoaponeurotic fibrosarcoma oncogene homolog (avian)	2.84	0.00295	1.31	0.34664
**KAL1**	Kallmann syndrome 1 sequence	2.86	0.00116	-1.21	0.44599
**ALDH2**	aldehyde dehydrogenase 2 family (mitochondrial)	2.86	0.00103	1.06	0.81366
**ATOH8**	atonal homolog 8 (Drosophila)	2.87	0.00687	-1.38	0.32394
**FLJ33630**	uncharacterized LOC644873	2.87	0.00018	1.38	0.11262
**U46120**	---	2.87	0.00822	1.19	0.60449
**NDST1**	N-deacetylase/N-sulfotransferase (heparan glucosaminyl) 1	2.89	0.01287	1.3	0.47444
**EGFL6**	EGF-like-domain, multiple 6	2.90	0.02041	1.18	0.68475
**MCCC2**	methylcrotonoyl-CoA carboxylase 2 (beta)	2.91	0.00769	1.05	0.87969
**KRT16**	keratin 16	2.92	0.00046	1.39	0.15277
**AA031832**	---	2.96	0.00914	1.07	0.84765
**B3GALT4**	UDP-Gal:betaGlcNAc beta 1,3-galactosyltransferase, polypeptide 4	2.98	0.00203	1.67	0.08386
**ZNF703**	zinc finger protein 703	2.99	0.00035	-1.37	0.16491
**FRY**	furry homolog (Drosophila)	3.00	0.00094	1.5	0.12302
**ZMYND12**	zinc finger, MYND-type containing 12	3.01	0.00254	1.9	0.04282
**CKMT1A**	creatine kinase, mitochondrial 1A	3.01	9.00E-05	1.37	0.10844
**SOX6**	SRY (sex determining region Y)-box 6	3.03	0.02047	1.12	0.78705
**BF844863**	---	3.04	6.00E-05	1.61	0.01858
**LGR6**	leucine-rich repeat containing G protein-coupled receptor 6	3.10	0.00011	1.3	0.18888
**KIAA1456**	KIAA1456	3.14	0.02984	1.71	0.26633
**C1orf54**	chromosome 1 open reading frame 54	3.15	0.00012	1.78	0.01332
**KRT13**	keratin 13	3.17	0.00511	1.35	0.38327
**ZMAT1**	zinc finger, matrin-type 1	3.19	0.00225	1.48	0.20645

**VIPR1**	vasoactive intestinal peptide receptor 1	3.29	0.00018	-1.1	0.65495
**IQCH-AS1**	IQCH antisense RNA 1 (non-protein coding)	3.34	0.00017	1.55	0.0642
**BF435593**	---	3.36	1.00E-04	-1.05	0.81941
**WNT10A**	Wingless-type MMTV integration site family, member 10A	3.38	0.00059	1.03	0.92006
**AA001390**	---	3.38	0.0182	1.21	0.67273
**WNT4**	wingless-type MMTV integration site family, member 4	3.39	0.00012	-1.12	0.57986
**GPNMB**	glycoprotein (transmembrane) nmb	3.47	0.01109	-1.55	0.30571
**GPR133**	G protein-coupled receptor 133	3.55	0.00646	1.21	0.62202
**AL357212**	---	3.57	0.00051	1.12	0.6731
**SMPD1**	sphingomyelin phosphodiesterase 1, acid lysosomal	3.59	1.00E-04	1.19	0.42387
**DYNLRB1**	dynein, light chain, roadblock-type 1	3.64	0	1.93	0.00083
**GGT6**	gamma-glutamyltransferase 6	3.68	4.00E-05	-1.07	0.72291
**OLR1**	oxidized low density lipoprotein (lectin-like) receptor 1	3.73	2.00E-05	1.22	0.2916
**LOC100292680**	uncharacterized LOC100292680	3.76	0.0032	-1.2	0.61063
**PLEKHB1**	pleckstrin homology domain containing, family B (evectins) member 1	3.77	0	-1.28	0.12964
**LGR5**	leucine-rich repeat containing G protein-coupled receptor 5	3.79	0.00017	-1.05	0.84761
**TPCN2**	two pore segment channel 2	3.81	3.00E-05	1.63	0.02946
**HIST3H2A**	histone cluster 3, H2a	3.82	1.00E-05	1.06	0.73512
**HSD17B10**	hydroxysteroid (17-beta) dehydrogenase 10	3.84	0	1.69	0.00572
**SLC47A2**	solute carrier family 47, member 2	3.86	2.00E-05	1.74	0.01412
**STX19**	syntaxin 19	3.89	7.00E-05	1.07	0.76025
**EDN2**	endothelin 2	4.04	0.00168	1.3	0.44591
**EDN1**	endothelin 1	4.04	0.00056	1.32	0.35072
**AK021431**	---	4.18	0.00837	1.13	0.78478
**AHNAK**	AHNAK nucleoprotein	4.47	3.00E-04	1.73	0.07986
**MAFB**	v-maf musculoaponeurotic fibrosarcoma oncogene homolog B (avian)	4.50	3.00E-05	1.57	0.05957
**UGT1A1**	UDP glucuronosyltransferase 1 family, polypeptide A1 /// UDP glucuronosyltransferase 1 family, polypeptide A10 /// UDP glucuronosyltransferase 1 family, polypeptide A3 /// UDP glucuronosyltransferase 1 family, polypeptide A4 /// UDP glucuronosyltransferase 1 family, polypeptide A5 /// UDP glucuronosyltransferase 1 family, polypeptide A6 /// UDP glucuronosyltransferase 1 family, polypeptide A7 /// UDP glucuronosyltransferase 1 family, polypeptide A8 /// UDP glucuronosyltransferase 1 family, polypeptide A9	4.64	4.00E-05	1.35	0.21751
**AK096324**	---	5.05	0.00021	1.1	0.75854
**MEGF6**	multiple EGF-like-domains 6	5.18	0	1.17	0.26274
**AHNAK2**	AHNAK nucleoprotein 2	5.50	2.00E-04	1.66	0.1261
**C18orf1**	chromosome 18 open reading frame 1	5.86	0.00027	-1.04	0.90742
**AXIN2**	axin 2	5.87	4.00E-05	1.21	0.4851
**RYR3**	ryanodine receptor 3	5.91	3.00E-05	1.15	0.59195
**KRT15**	keratin 15	5.94	2.00E-05	1.44	0.15179
**CYP1A1**	cytochrome P450, family 1, subfamily A, polypeptide 1	5.96	0.00111	-1.53	0.3133

**CYP2E1**	cytochrome P450, family 2, subfamily E, polypeptide 1	6.35	4.00E-05	1.5	0.17043
**WFDC5**	WAP four-disulfide core domain 5	6.72	0.00018	-1.03	0.92517

Table S3. A list of common differentially expressed genes affected by CRTC1-MAML2- or CREB-depletion in human MEC H3118 cells. This gene list represents candidate genes regulated by fusion/CREB interaction. The "positive" and "negative" signs denote up-regulated or down-regulated genes in KD compared to control groups, respectively. The asterisk indicates the common gene in different regulatory direction between fusion and CREB.

Gene Symbol	Gene Title	Fusion Fold Change	Fusion p.value	CREB Fold Change	CREB p.value
**LINC00473**	chromosome 6 open reading frame 176	-37.12	0	-3.6	6.00E-05
**DMBT1**	deleted in malignant brain tumors 1	-35.73	0	-8.39	5.00E-05
**STC1**	stanniocalcin 1	-21.74	0	-26.98	2.00E-05
**LOC100288985**	Hypothetical protein LOC100288985	-17.73	0	-41.04	8.00E-05
**NR4A3**	nuclear receptor subfamily 4, group A, member 3	-16.29	1.00E-05	-6.19	3.00E-05
**AW302848**	---	-12.93	0.00013	-2.25	0.00219
**OSBP2**	oxysterol binding protein 2	-9.52	1.00E-05	-2.94	0.00121
**SORBS2**	sorbin and SH3 domain containing 2	-8.42	6.00E-04	-4.23	0.00779
**LUM**	lumican	-6.98	0.00061	-4.16	0.03358
**HAL**	histidine ammonia-lyase	-6.79	2.00E-05	-9.33	0
**SMOC1**	SPARC related modular calcium binding 1	-6.47	0	-2.92	0.00125
**BC027448**	---	-6.45	0.00045	-10.18	0
**BC020911**	---	-6.44	0.00377	-3.41	0.00068
**NR4A1**	nuclear receptor subfamily 4, group A, member 1	-6.14	4.00E-05	-2.08	7.00E-05
**NPR3**	natriuretic peptide receptor C/guanylate cyclase C (atrionatriuretic peptide receptor C)	-6	0.00019	-2.45	0.02913
**AA523939**	---	-6	1.00E-05	-2.17	0.00911
**CREG2**	cellular repressor of E1A-stimulated genes 2	-5.91	2.00E-05	2.03	0.02738
**CHL1-AS2**	CHL1 antisense RNA 2	-5.54	0.00083	-5.91	2.00E-05
**LGSN**	lengsin, lens protein with glutamine synthetase domain	-5.27	2.00E-05	-3.65	0.02779
**MFSD2A**	major facilitator superfamily domain containing 2A	-4.96	5.00E-05	-10.08	0
**AI476341**	---	-4.87	0.00659	-3.04	5.00E-05
**DUSP1**	dual specificity phosphatase 1	-4.81	0.00017	-2.15	0.03823
**A2M**	alpha-2-macroglobulin	-4.62	0.00102	-2.26	0.01347
**AK022474**	---	-4.51	0.01964	-3.25	0.00018
**CHL1**	Cell adhesion molecule with homology to L1CAM	-4.47	0.00163	-2.92	0.03528
**ZNF331**	zinc finger protein 331	-4.24	8.00E-05	-3.73	1.00E-05
**IL20**	interleukin 20	-4.23	0.00345	-4.53	0.0017
**ENO3**	enolase 3 (beta, muscle)	-4.14	0.00033	-3.19	8.00E-05
**AI806183**	---	-4.1	0.00016	-3.55	0.00021
**EOMES**	eomesodermin	-4.1	1.00E-05	-2.57	0.00381
**SRGN**	serglycin	-3.95	0.00518	-3.18	3.00E-04
**DUSP4**	dual specificity phosphatase 4	-3.93	0.00155	-5.23	0
**NM_024853**	---	-3.92	0.00013	-2.12	0.03494
**PTGS1**	prostaglandin-endoperoxide synthase 1 (prostaglandin G/H synthase and cyclooxygenase)	-3.89	0	-7.16	9.00E-05
**AV702787**	---	-3.81	0.00075	-2.31	0.00058
**BG112263**	---	-3.55	3.00E-05	-3.45	1.00E-04
**CALCA**	calcitonin-related polypeptide alpha	-3.49	0.00643	-2.85	0.0117
**SLCO4A1**	solute carrier organic anion transporter family, member 4A1	-3.46	0	-7.92	6.00E-05
**CDH11**	cadherin 11, type 2, OB-cadherin (osteoblast)	-3.38	0.00044	-10.27	0
**NCALD**	neurocalcin delta	-3.34	5.00E-05	-2.33	0.00014
**LOC100506870**	hypothetical LOC100506870	-3.24	0.00071	-10.04	0.00016
**GRAMD1B**	GRAM domain containing 1B	-3.24	0.00167	-4.47	5.00E-05

**SLC2A14**	solute carrier family 2 (facilitated glucose transporter), member 14	-3.2	0.00037	-2.13	0.00724
**LOC283713**	hypothetical protein LOC283713	-3.19	0.01709	2.06	0.01445
**FAM83A**	family with sequence similarity 83, member A	-3.18	0.00238	-3.38	0.00279
**AA416756**	---	-3.16	0.00578	-2.44	0.00676
**SFRP1**	secreted frizzled-related protein 1	-3.12	0.00018	-4.85	4.00E-05
**F2RL2**	coagulation factor II (thrombin) receptor-like 2	-3.11	0.00039	-5.63	0.00428
**MGC39372**	serpin peptidase inhibitor, clade B (ovalbumin), member 9 pseudogene	-3.09	0.00106	-2.3	0.0039
**TPRG1**	tumor protein p63 regulated 1	-3.06	3.00E-05	-4.22	0.00033
**RASD1**	RAS, dexamethasone-induced 1	-3.05	1.00E-04	-4.96	2.00E-05
**STEAP4**	STEAP family member 4	-3.03	0.00022	-4.27	0.00091
**E2F2**	E2F transcription factor 2	-3.03	0.0024	-2.51	0.04144
**SERPINB9**	serpin peptidase inhibitor, clade B (ovalbumin), member 9	-2.98	0.00472	-6.82	0
**SYT12**	synaptotagmin XII	-2.97	0.00015	-4.85	1.00E-05
**SMOX**	spermine oxidase	-2.95	0.01324	-21.45	0
**ATP6V0A4**	ATPase, H+ transporting, lysosomal V0 subunit a4	-2.9	0.00544	-13.97	0
**TNFRSF10B**	tumor necrosis factor receptor superfamily, member 10b	-2.89	0.00018	-2.31	0.02723
**XDH**	xanthine dehydrogenase	-2.87	0.02018	-3	0.00331
**BC037339**	---	-2.86	0.00021	-2.27	0.01655
**LOC338651**	hypothetical LOC338651	-2.82	0.04575	2.6	0.02727
**PTGES**	prostaglandin E synthase	-2.81	0.0132	-2.33	0.00273
**SEMA3B**	sema domain, immunoglobulin domain (Ig), short basic domain, secreted, (semaphorin) 3B	-2.78	0.00031	-2.3	0.00081
**XPR1**	xenotropic and polytropic retrovirus receptor 1	-2.77	0.00322	-4.14	0.00657
**CA9**	carbonic anhydrase IX	-2.77	0.00402	-3.29	0.03444
**RAET1E**	retinoic acid early transcript 1E	-2.72	0.02536	-3.26	3.00E-05
**SPRY1**	sprouty homolog 1, antagonist of FGF signaling (Drosophila)	-2.7	0.01542	-2.73	0.00472
**ULBP2**	UL16 binding protein 2	-2.67	0.00021	-2.79	0.00095
**SLC2A3**	solute carrier family 2 (facilitated glucose transporter), member 3	-2.65	0.00144	-4.22	0.00034
**KYNU**	kynureninase (L-kynurenine hydrolase)	-2.63	0.00098	-6.23	0
**BF590850**	---	-2.6	4.00E-05	-3.5	0.00171
**GPR133**	G protein-coupled receptor 133	-2.52	0.00279	3.55	0.00646
**ANO3**	anoctamin 3	-2.5	0.00317	-5.59	0
**PIGH**	phosphatidylinositol glycan anchor biosynthesis, class H	-2.5	0.00017	-2.63	0.00153
**NID2**	nidogen 2 (osteonidogen)	-2.49	0.00011	-3.68	0.00591
**TGFB2**	transforming growth factor, beta 2	-2.48	0.00244	-4.76	0.00034
**PSD3**	pleckstrin and Sec7 domain containing 3	-2.47	0.03327	-2.05	0.0173
**RASSF10**	Ras association (RalGDS/AF-6) domain family (N-terminal) member 10	-2.45	0.00684	-2.16	0.01003
**LOC100128653**	hypothetical LOC100128653	-2.44	0.00019	-2.09	0.00161
**SSH1**	slingshot homolog 1 (Drosophila)	-2.42	0.00079	-3.47	0.00743
**FEZ1**	fasciculation and elongation protein zeta 1 (zygin I)	-2.42	9.00E-05	-3.39	0.00021
**KLF9**	Kruppel-like factor 9	-2.4	0.00169	-6.3	0.00242
**CENPV**	centromere protein V	-2.4	0.00211	-3.39	0.00011
**RNF157-AS1**	RNF157 antisense RNA 1	-2.4	0.01288	-2.21	0.03193
**AI761207**	---	-2.39	0.00236	-3.14	0.00697
**BMP8B**	bone morphogenetic protein 8b	-2.38	0.00438	-2.79	7.00E-05
**WDR76**	WD repeat domain 76	-2.35	0.00499	-2.98	0.00247

**BE271180**	---	-2.34	0.00459	-2.72	0.01322
**CASP9**	caspase 9, apoptosis-related cysteine peptidase	-2.33	0.00016	-2.17	4.00E-05
**C11orf86**	chromosome 11 open reading frame 86	-2.31	0.00125	-4.34	0.00013
**ZNF367**	zinc finger protein 367	-2.31	0.00111	-2.37	0.03245
**LOC728431**	hypothetical LOC728431	-2.3	0.00174	-2.36	0.01613
**SLC25A45**	solute carrier family 25, member 45	-2.3	0.01443	-2.07	0.01745
**PER1**	period homolog 1 (Drosophila)	-2.28	0.00018	-2.37	0.02402
**IL24**	interleukin 24	-2.27	0.04771	-3.43	0.04046
**AW611560**	---	-2.26	0.00519	-2.13	0.00268
**AI686793**	---	-2.25	0.00066	-2.61	0.00132
**AREG**	amphiregulin	-2.23	0.04495	-18.34	0
**EDNRB**	endothelin receptor type B	-2.23	0.00037	-2.86	0.02402
**GYG2**	glycogenin 2	-2.23	0.00261	-2.6	0.00267
**BF445387**	---	-2.23	0.0027	-2.13	0.00411
**KIAA1549**	flavin containing monooxygenase 4	-2.22	0.00013	-3	0
**STAMBPL1**	STAM binding protein-like 1	-2.2	0.0049	-2.82	4.00E-05
**MCM10**	minichromosome maintenance complex component 10	-2.19	0.04848	-3.59	8.00E-05
**ZBTB38**	zinc finger and BTB domain containing 38	-2.19	0.04405	-2.28	0.00617
**DNASE1L3**	deoxyribonuclease I-like 3	-2.18	0.00419	-3.56	0.00101
**ARID5B**	AT rich interactive domain 5B (MRF1-like)	-2.18	0.00052	2.54	0.01386
**PC**	pyruvate carboxylase	-2.17	0.00064	-2.28	0.00514
**FAM216A**	family with sequence similarity 216, member A	-2.16	0.00016	-5.08	0.00011
**PRKCH**	protein kinase C, eta	-2.16	0.00061	-3.73	0.00024
**BRI3BP**	BRI3 binding protein	-2.15	0.0253	-2.67	0.00234
**AI300077**	---	-2.15	0.00412	-2.54	4.00E-05
**LOC100506021**	hypothetical LOC100506021	-2.15	0.01054	-2.25	0.00178
**ITPR1**	inositol 1,4,5-triphosphate receptor, type 1	-2.14	0.00162	-2.45	0.00145
**DLGAP1-AS1**	DLGAP1 antisense RNA 1	-2.14	0.04809	-2.01	0.00014
**DST**	dystonin	-2.11	0.03694	-2.13	0.01481
**LOC158696**	hypothetical LOC158696	-2.11	0.00213	2.4	0.01369
**TYMS**	thymidylate synthetase	-2.1	0.02143	-2.46	0.00819
**MSX2**	msh homeobox 2	-2.1	0.01319	2.22	0.02264
**MCTP2**	multiple C2 domains, transmembrane 2	-2.09	0.00189	-2.65	0.01823
**FNDC3A**	Fibronectin type III domain containing 3A	-2.09	0.04589	-2.55	0.01236
**AW293012**	---	-2.09	0.00093	-2.41	0.00121
**AA088388**	---	-2.08	0.04262	-2.06	0.01107
**CYFIP2**	cytoplasmic FMR1 interacting protein 2	-2.07	0.0021	2.21	0.00134
**AL049245**	---	-2.06	0.00369	-5.75	0
**SSFA2**	sperm specific antigen 2	-2.05	0.00073	-2.05	0.03658
**NCAM1**	Neural cell adhesion molecule 1	-2.04	0.00845	-3.48	0.00082
**SMAD2**	SMAD family member 2	-2.04	0.00215	-2.49	3.00E-05
**RGCC**	regulator of cell cycle	-2.03	0.03419	-3.21	0.02073
**TMEM231**	transmembrane protein 231	-2.03	0.02144	-2.76	0.00393
**C11orf96**	chromosome 11 open reading frame 96	-2.03	0.01409	-2.36	0.00313
**ATOH8**	Atonal homolog 8 (Drosophila)	-2.02	0.03864	2.87	0.00687
**PRKAG2-AS1**	PRKAG2 antisense RNA 1	-2.01	0.01612	-2.66	0.02381
**TRAF3IP3**	TRAF3 interacting protein 3	-2.01	0.00273	-2.31	0.00154
**ERGIC1**	endoplasmic reticulum-golgi intermediate compartment (ERGIC) 1	-2.01	0.00621	-2.27	0.04547
**LINC00313**	long intergenic non-protein coding RNA 313	-2	0.01814	-2.69	0.00567
**HOXA10**	homeobox A10	2.01	0.00161	2.04	0.00012
**NREP**	neuronal regeneration related protein homolog (rat)	2.01	0.04319	2.79	9.00E-05

**PADI3**	peptidyl arginine deiminase, type III	2.03	0.00887	-2.73	2.00E-05
**HS3ST1**	heparan sulfate (glucosamine) 3-O-sulfotransferase 1	2.03	0.02889	-2.03	0.01363
**PRKCA**	protein kinase C, alpha	2.04	0.00066	-2.31	0.00127
**GPR37**	G protein-coupled receptor 37 (endothelin receptor type B-like)	2.05	0.02429	-2.05	0.00053
**RASL11B**	RAS-like, family 11, member B	2.08	0.00127	-3.16	0.00068
**PI3**	peptidase inhibitor 3, skin-derived	2.08	0.03815	-2.98	0.00364
**IRAK2**	interleukin-1 receptor-associated kinase 2	2.1	0.00105	-2.22	0.00285
**WFDC2**	WAP four-disulfide core domain 2	2.14	0.00652	-2.78	0.00024
**CYP1B1**	cytochrome P450, family 1, subfamily B, polypeptide 1	2.14	0.00679	2.49	0.00047
**SERTAD4**	SERTA domain containing 4	2.17	0.00122	-2.66	0.00373
**ETS1**	v-ets erythroblastosis virus E26 oncogene homolog 1 (avian)	2.17	0.00029	-2.09	0.0024
**WNT10A**	Wingless-type MMTV integration site family, member 10A	2.17	0.00265	3.38	0.00059
**LOC728978**	uncharacterized LOC728978	2.18	0.00056	2.18	0.02444
**MARCKS**	myristoylated alanine-rich protein kinase C substrate	2.18	0.00228	2.52	0.0027
**LOC100292680**	hypothetical LOC100292680	2.19	0.00376	3.76	0.0032
**SERPINA1**	serpin peptidase inhibitor, clade A (alpha-1 antiproteinase, antitrypsin), member 1	2.27	0.00614	-4.65	0.00044
**AI936724**	---	2.27	0.00302	-2.9	0.00923
**CYP1A1**	cytochrome P450, family 1, subfamily A, polypeptide 1	2.29	0.0261	5.96	0.00111
**RUNX1**	runt-related transcription factor 1	2.3	0.00957	-3.32	0.00111
**AW189097**	---	2.3	0.01593	2.46	0.03749
**MAF**	v-maf musculoaponeurotic fibrosarcoma oncogene homolog (avian)	2.31	0.01038	2.84	0.00295
**AI223854**	---	2.34	0.00425	2.15	0.00628
**IFITM10**	interferon induced transmembrane protein 10	2.38	0.04561	2.31	0.02374
**UPP1**	uridine phosphorylase 1	2.39	0.02813	-2.75	0.0041
**COL5A1**	collagen, type V, alpha 1	2.41	0.00067	2.09	0.00254
**CD44**	CD44 molecule (Indian blood group)	2.62	0.00099	-2.4	0.00231
**CORO2B**	coronin, actin binding protein, 2B	2.72	0.0018	-2.76	0.00062
**LRRN1**	leucine rich repeat neuronal 1	2.79	8.00E-05	-2.82	0.03339
**KRT6B**	keratin 6B	2.84	0.00038	-2.22	0.0132
**FAM49A**	family with sequence similarity 49, member A	2.84	9.00E-05	-2.04	0.00519
**DUSP5P**	dual specificity phosphatase 5 pseudogene	2.89	5.00E-04	2.35	0.00587
**ALDH1A3**	aldehyde dehydrogenase 1 family, member A3	2.92	0.00026	-4.04	0.00105
**TUBA1A**	tubulin, alpha 1a	2.94	0.00044	-2.31	0.04361
**THBD**	thrombomodulin	2.96	0.00033	-3.29	0.00486
**RASSF6**	Ras association (RalGDS/AF-6) domain family member 6	3.01	0.00019	2.17	0.02682
**KRT16**	keratin 16	3.04	0.00032	2.92	0.00046
**UGT1A1**	UDP glucuronosyltransferase 1 family, polypeptide A1	3.06	0.00336	4.64	4.00E-05
**MYLK**	myosin light chain kinase	3.09	0.00691	2.58	0.00098
**TAGLN**	transgelin	3.1	0.04019	2.34	0.03019
**MAFB**	v-maf musculoaponeurotic fibrosarcoma oncogene homolog B (avian)	3.2	0.01557	4.5	3.00E-05
**TNFSF10**	tumor necrosis factor (ligand) superfamily, member 10	3.34	0.00567	-2.84	0.00748

**PLAU**	plasminogen activator, urokinase	3.55	0.00081	-6.56	0
**AHNAK2**	AHNAK nucleoprotein 2	3.58	0.00281	5.5	2.00E-04
**SLC47A2**	solute carrier family 47, member 2	3.61	0.00311	3.86	2.00E-05
**ERC1**	ELKS/RAB6-interacting/CAST family member 1	4.15	9.00E-05	2.5	0.00103
**MMP28**	matrix metallopeptidase 28	4.23	0.00033	-2.46	0.02048
**EPHA4**	EPH receptor A4	4.3	0.00042	2.53	0.02907
**FABP4**	fatty acid binding protein 4, adipocyte	4.45	0.01837	-2.57	0.02591
**C18orf1**	chromosome 18 open reading frame 1	4.58	0.0013	5.86	0.00027
**GPR39**	G protein-coupled receptor 39	4.64	0.00029	2.04	0.01846
**MTSS1**	metastasis suppressor 1	4.83	0.00047	-2.12	0.02179
**FBXO32**	F-box protein 32	5.68	0.00022	2.44	0.02761
**SERPINA3**	serpin peptidase inhibitor, clade A (alpha-1 antiproteinase, antitrypsin), member 3	5.78	0.00155	-4.85	8.00E-04
**KRT15**	keratin 15	6.65	0.00113	5.94	2.00E-05
**MMP1**	matrix metallopeptidase 1 (interstitial collagenase)	11.48	7.00E-05	-2.13	0.00423
**SERPINE1**	serpin peptidase inhibitor, clade E (nexin, plasminogen activator inhibitor type 1), member 1	15.77	0	-2.95	0.03059
**GPNMB**	glycoprotein (transmembrane) nmb	16	2.00E-05	3.47	0.01109
